# Synthesis and Antiproliferative Activity of Phosphorus Substituted 4-Cyanooxazolines, 2-Aminocyanooxazolines, 2-Iminocyanooxazolidines and 2-Aminocyanothiazolines by Rearrangement of Cyanoaziridines

**DOI:** 10.3390/molecules26144265

**Published:** 2021-07-14

**Authors:** Victor Carramiñana, Ana M. Ochoa de Retana, Francisco Palacios, Jesús M. de los Santos

**Affiliations:** Department of Organic Chemistry I, Faculty of Pharmacy and Lascaray Research Center, University of the Basque Country (UPV/EHU), Paseo de la Universidad 7, 01006 Vitoria, Spain; victor.carraminana@ehu.eus (V.C.); anamaria.ochoaderetana@ehu.eus (A.M.O.d.R.)

**Keywords:** phosphorus substituted cyanoaziridines, 4-cyanooxazolines, 2-aminocyanooxazolines, 2-iminocyanooxazolidines, 2-aminocyanothiazolines, antiproliferative effect

## Abstract

Several phosphorus-substituted *N*-acylated cyanoaziridines **2** and *N*-carbamoylated cyanoziridines **5** were prepared in good to high yields. *N*-Acylated cyanoaziridines **2** were used, after ring expansion, in an efficient synthesis of oxazoline derivative **3a** and in a completely regio-controlled reaction in the presence of NaI. Conversely, *N*-carbamoyl cyanoaziridines **5** reacted with NaI to obtain a regioisomeric mixture of 2-aminocyanooxazolines **7**. Mild acidic conditions can be used for the isomerization of *N*-thiocarbamoyl cyanoaziridine **6a** into a 2-aminocyanothiazoline derivative **8a** by using BF_3_·OEt_2_ as a Lewis acid. Likewise, a one pot reaction of *N*H-cyanoaziridines **1** with isocyanates obtained 2-iminocyanooxazolidines **9** regioselectively. This synthetic methodology involves the addition of isocyanates to starting cyanoaziridines to obtain *N*-carbamoyl cyanoaziridines **5**, which after the ring opening, reacts with a second equivalent of isocyanate to give the final 2-imino cyanooxazolidines **9**. In addition, the cytotoxic effect on the cell lines derived from human lung adenocarcinoma (A549) was also screened. 2-Iminooxazolidines **9** exhibited moderate activity against the A549 cell line in vitro. Furthermore, a selectivity towards cancer cells (A549) over non-malignant cells (MCR-5) was detected.

## 1. Introduction

**Figure 1:**
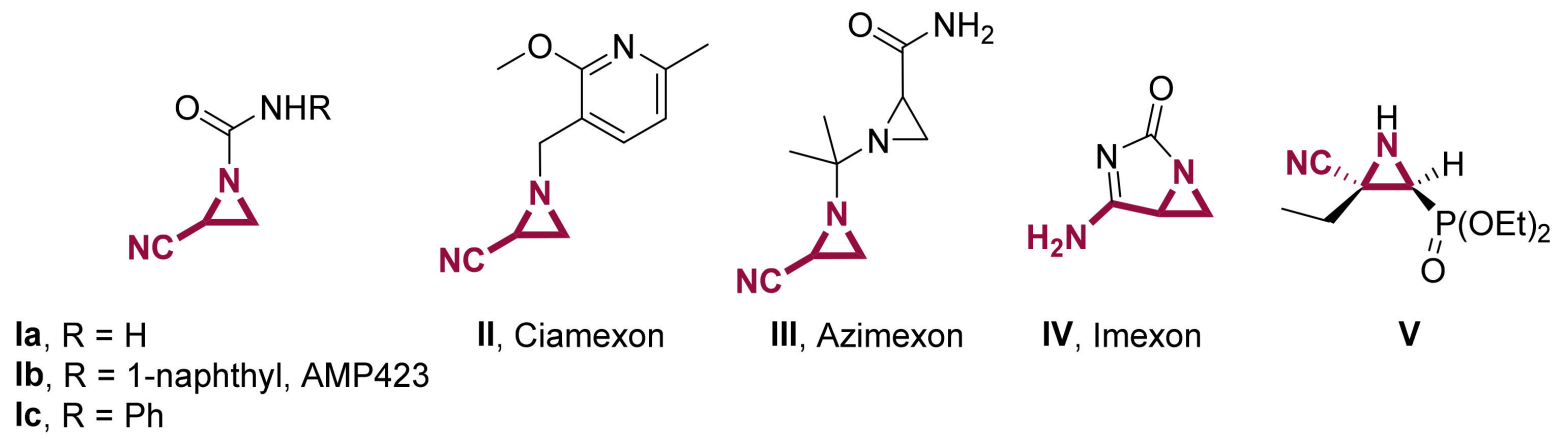
Representative examples of 2-cyanoaziridines as antitumor drugs.

DNA-modifying agents are a significant class of pharmaceuticals used in conventional chemotherapy. Aziridine-based cytostatic compounds, acting as powerful alkylating agents, have an inherent in vivo potency due to their ability to act as DNA cross-linking agents via the ring opening of aziridine [1]. Mitomycin C and many variants of this natural product have been well characterized for their anti-tumor activity based on the nucleophilic ring opening of the three-membered nitrogen heterocycle, leading to the alkylation of DNA [2]. Mitomycin C is a conventional DNA cross-linking agent that uses the reductive activation of the aziridine moiety to form lethal DNA–DNA cross-links, as well as, more often, mono-alkylated DNA products [3]. Founded on this knowledge, during the early 1970s Bicker [4,5] developed a variety of 2-cyanoaziridine derivatives as potential carcinostatic agents. For instance, 2-cyanoaziridine-1-carboxamide (**Ia**, Figure 1) was active against a PIE 2-3 sarcoma in Wistar rats, and it had a low toxicity. However, it showed weak antitumor activity in cell cultures. The fact that it increased rather than decreased the number of leukocytes was especially interesting [5]. However, in contrast to the initial findings, these cyanoaziridines showed no alkylating activity in vitro or in vivo [4]. These results suggest that the cyano group reduces the reactivity required for the alkylation of DNA bases and that they may selectively react with sulfur moieties in biological thiols such as cysteine, depleting the stores of cysteine and glutathione and subsequently allowing the accumulation of cellular reactive oxygen species (ROS) [6,7,8]. Preclinical studies have evaluated the antitumor activity and the mechanism of action of cyanoaziridine AMP423 (**Ib**, Figure 1) [6]. Other cyanoaziridines such as ciamexon (**II**) and azimexon (**III**, Figure 1) [9] were screened for antitumor activity, with brief clinical trials in the late 1980s. Both ciamexon (**II**) and azimexon (**III**) displayed antitumor activity in a variety of animal models including the Lewis lung tumor, the Madison lung carcinoma, Meth A sarcoma, and AKR leukemia, but they had no direct antitumor activity in vitro according to Bicker [5] and they are no longer used. The iminopyrrolidone compound imexon (**IV**), produced by the cyclization of 2-cyanoaziridine-1-carboxamide (**Ia**) in the presence of hydroxide ions [10,11], is an anti-neoplastic agent that increases oxidative stress in the target and it has been extensively used due to its selective growth inhibitory effect against multiple myeloma [12,13,14]. This small-molecule chemotherapeutic agent is widely used to treat advanced cancers of the breast, lung, and prostate. The biological activity of imexon is narrowly associated with cyanoaziridines since imexon solutions in water slowly revert to **Ia**. In 1999, Remers et al. [15] reported the synthesis of a series of 2-cyanoaziridine-1-carboxamides that were active against a variety of solid and hematological tumor cells in culture. The *N*-Phenyl derivative (**Ic**, R = Ph, Figure 1) tested in human tumor cells was found to be related to imexon in activity. More recently, our research group reported the preparation of phosphorus substituted cyanoaziridines and evaluated them by testing their antiproliferative activities against different human cancer cell lines. For instance, the phosphonate-derived cyanoaziridine **V** showed in vitro cytotoxicity against the A549 cell line with an IC_50_ value of 1.5 ± 0.84 µM [16].

Furthermore, the ring-expansion of aziridines with isocyanates has been revealed to be a useful pathway for the synthesis of a variety of five-membered nitrogen-containing compounds. For instance, KI [17], NaI [18,19,20], NiI_2_ [21], Pd-catalyzed [22,23,24] reactions of aziridines with isocyanates, or even in the absence of catalysts [25], have been described. All these reactions yielded imidazolidin-2-ones or oxazolidin-2-imines compounds which generate great interest in medicinal and pharmaceutical chemistry since they represent classes of heterocyclic compounds with a broad variety of biological activities [26,27]. In the last few years we have been involved in the chemistry related to phosphorylated 2*H*-azirines for the preparation of α- or β-aminophosphonic acid derivatives [28,29,30], pyrroles [31,32], oxazoles [33], 1*H*-benzo[*d*]azepines [31], or hybrid molecules such as azirino [2,1-*b*]benzo[*e*][1,3]oxazines [34], among others. Additionally, organophosphorus derivatives are fascinating compounds from a biological point of view, due to the fact that these substituents may regulate relevant biological functions modifying the reactivity of heterocyclic systems [35]. Recently, we have revealed a diastereoselective method for the preparation of phosphorus substituted cyanoaziridines by means of the nucleophilic addition of TMSCN as a cyanide source to the C–N double bond of 2*H*-azirines [16]. Following our earlier studies on the preparation of phosphorylated cyanoaziridines, here we wish to report the synthesis of structurally new phosphorus substituted *N*-(thio)carbamoyl cyanoaziridines by the coupling of unactivated cyanoaziridines with iso(thio)cyanates. Moreover, the ring expansion into 2-aminocyanooxazolines, 2-aminocyanothiazolines, or 2-iminocyanooxazolidines under nucleophilic or acidic conditions is also explored. We also focus on their biological activity and highlight the antiproliferative effect of all these new heterocycles on A549 human lung adenocarcinoma cells.

## 2. Results

### 2.1. Chemistry

**Scheme 1:**
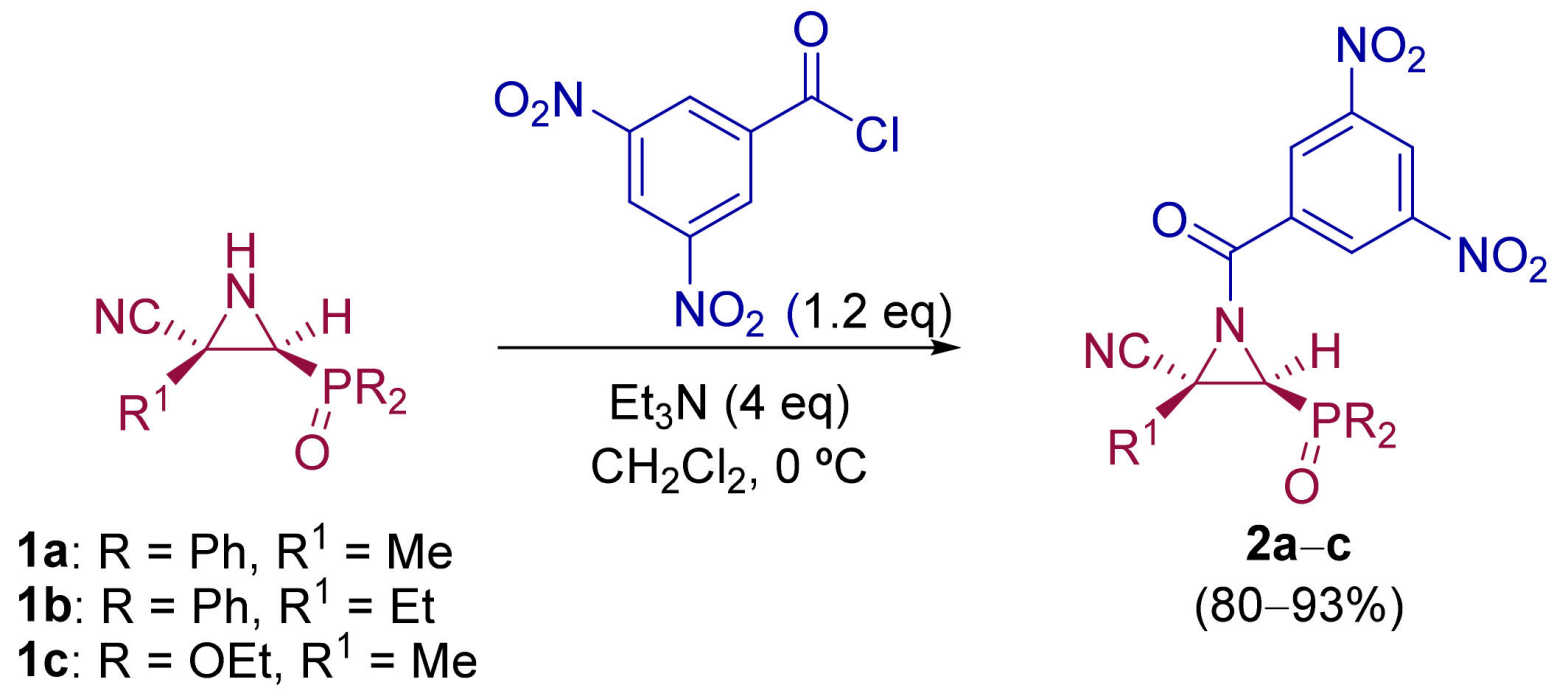
Synthesis of *N*-acylated cyanoaziridines phosphine oxides and phosphonates **2**.

As a continuation of the studies on the synthesis and applications of activated aziridines through the *N*-functionalization of unactivated cyanoaziridines [16], and taking into account that *N*-acylaziridines are very important synthons in the development of new ring opening aziridine reactions, we initially studied the *N*-acylation reaction of cyanoaziridine phosphine oxides **1a** (R = Ph, R^1^ = Me) and **1b** (R = Ph, R^1^ = Et), and phosphonate **1c** (R = OEt, R^1^ = Me). Thus, the *N*-acylation of cyanoaziridines **1a**–**c** using 3,5-dinitrobenzoyl chloride in the presence of a base, such as Et_3_N, and methylene chloride as the solvent, obtained *N*-acylated cyanoziridines **2a**–**c** in good chemical yields (80–93%) (Scheme 1).

**Scheme 2:**
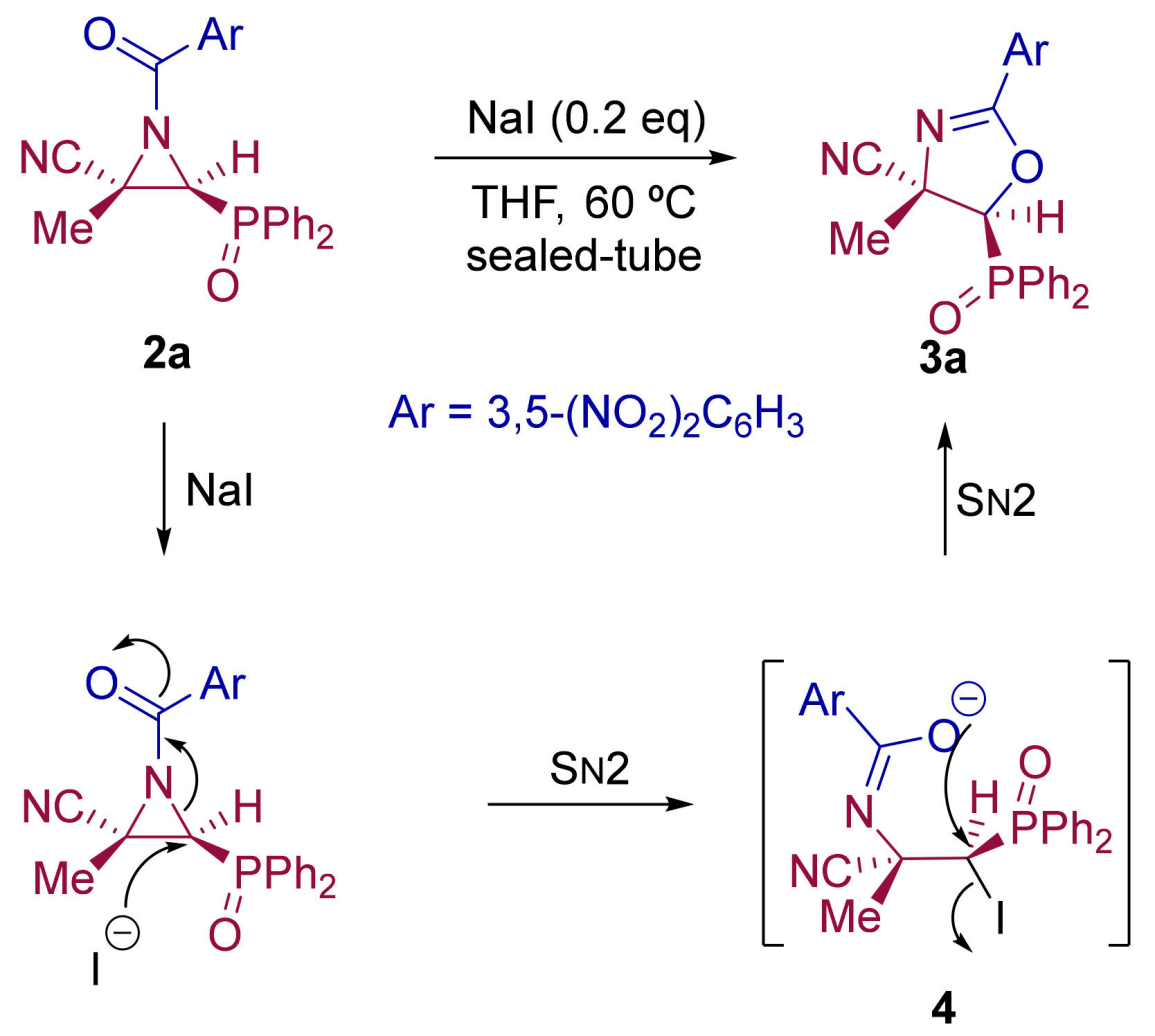
Ring expansion of cyanoaziridine **2a** to oxazoline **3a**.

We next studied the isomerization reaction (Heine reaction) of the corresponding synthesized phosphorus-containing *N*-acylated cyanoaziridines **2**. For this purpose, we chose nucleophilic conditions [36,37,38,39], and the isomerization of benzoyl aziridine **2a** was accomplished by the use of the sodium iodide method. Indeed, when phosphorus substituted *N*-acylated cyanoaziridine **2a** reacted with 0.2 equivalents of NaI in THF at 60 °C in a sealed-tube, 4-cyanooxazoline derived from phosphine oxide **3a** was obtained in a regioselective way in a 65% yield (Scheme 2).

Even though the iodide anion may attack both the aziridine carbon atoms, and the regioselectivity will be determined by the stereoelectronic nature of the substituents, several reports in the literature describe the aziridine ring opening through the less hindered position [40,41,42]. Bear in mind that the most reasonable mechanism may consider a first step where the iodide anion would attack at the C2 aziridine carbon that was substituted less in a regiospecific manner, followed by the aziridine ring opening obtaining intermediate **4**. The iodide displacement in the former intermediate may afford phosphorus substituted 4-cyanooxazoline **3a** (Scheme 2).

Unactivated *N*H-aziridines are very stable compounds in basic conditions and may easily react with electrophiles. Next, we explored the *N*-functionalization of unactivated cyanoaziridines derived from phosphine oxides and phosphonates **1** with aromatic and aliphatic isocyanates for the preparation of functionalized *N*-aryl or *N*-alkylcarbamoyl cyanoaziridines.

Therefore, the reaction of cyanoaziridines **1** with isocyanates was assessed. Thus, as outlined in Table 1, in an initial experiment the addition of phenyl isocyanate to cyanoaziridine phosphine oxide **1a** (R = Ph, R^1^ = Me) was readily achieved in dichloromethane at room temperature. A total of 1.2 equivalents of the isocyanate component were used in order to ensure a full conversion. Under these reaction conditions, the corresponding phosphorus substituted *N*-phenylcarbamoyl cyanoaziridine **5a** (R = R^2^ = Ph, R^1^ = Me) was obtained in a 63% yield (Table 1, entry 1).

Some examples in the literature describe the use of Lewis acids as transition metal catalysts involving reactions of aziridines and isocyanates [22,43,44]. Hence, we explored the reaction of phenyl isocianate with cyanoaziridine **1a** in the presence of a Lewis acid. Better yields and reduced reaction times were observed for the synthesis of **5a** using catalytic ammounts of Sc (OTf)_3_ (20%) (see Table 1, entry 2). In the same way, phosphate-derived cyanoziridine **1c** (R = OEt, R^1^ = Me) reacted with phenyl isocyanate in CH_2_Cl_2_ at room temperature, without a catalyst, to give *N*-functionalized cyanoaziridine **5b** (R = OEt, R^1^ = Me, R^2^ = Ph) in very good chemical yields (Table 1, entry 3).

As illustrated in Table 1, this synthetic methodology is tolerant of a variety of functionalized isocyanates with varying substitutions. For instance, cyanoaziridines **1a** and **1c** reacted with *p*-tolyl isocyanate (R^2^ = *p*-MeC_6_H_4_) to give *N*-*p*-tolylcarbamoyl cyanoaziridines **5c** and **5d**, respectively (Table 1, entries 4 and 5). Likewise, under the same reaction conditions, cyanoaziridine **1a** reacted with *p*-toluenesulfonyl isocyanate. The crude product **5** (R = Ph, R^1^ = Me, R^2^ = *p*-MeC_6_H_4_SO_2_) was observed by ^1^H and ^31^P NMR; however, any further purification step through crystallization or chromatography produced the hydrolyzed starting cyanoaziridine **1a**. Conversely, *N*-*p*-toluenesulfonylcarbamoyl cyanoaziridines **5e** (R = Ph, R^1^ = Et, R^2^ = *p*-MeC_6_H_4_SO_2_, Table 1, entry 6) and **5f** (R = OEt, R^1^ = Me, R^2^ = *p*-MeC_6_H_4_SO_2_, Table 1, entry 7) were obtained in 86 and 79% yields, respectively, by means of the treatment of the corresponding cyanoaziridines **1b** and **1c** with *p*-toluenesulfonyl isocyanate.

This process was extended to the reactivity of phosphorus substituted cyanoziridines **1** with alkyl isocyanates, such as ethyl and *tert*-butyl isocyanate. The same reaction conditions used for the aromatic isocyanates were employed for the reaction of **1a** with ethyl isocyanate. Nevertheless, no progress was observed on the formation of compound **5g**, and the starting cyanoaziridine **1a** was recovered instead. The Lewis acid activation of the aziridine ring in the reaction of **1a** with ethyl isocyanate led to the formation of the expected compound **5g**. Thus, the presence of 20% mol of Sc (OTf)_3_ as a Lewis acid, as described before for the synthesis of **5a**, gave only a 50% conversion of *N*-ethylcarbamoyl cyanoaziridine **5g** (R = Ph, R^1^ = Me, R^2^ = Et) after 24 h of the reaction. However, when two equivalents of ethyl isocyanate reacted with cyanoaziridine **1a** in the presence of 1.25 equivalents of ZnCl_2_ in CH_2_Cl_2_ and at room temperature, *N*-functionalized cyanoaziridine **5g** was obtained with a 86% chemical yield (Table 1, entry 8). Similarly, the addition of ethyl isocyanate to phosphonate-derived cyanoaziridine **1c**, using ZnCl_2_ as a Lewis acid, led to the formation of *N*-functionalized aziridine **5h** (R = OEt, R^1^ = Me, R^2^ = Et; Table 1, entry 9). In addition, the synthesis of *N*-*tert*-butylcarbamoyl cyanoaziridines **5i**–**k** (Table 1, entries 10–12) was achieved in moderate yields, using *tert*-butyl isocyanate as an electrophile and ZnCl_2_ as a Lewis acid.

The synthetic procedure for the preparation of *N*-aryl or *N*-alkylcarbamoyl cyanoaziridines **5** could be widened to the addition of isothiocyanates to cyanoaziridines **1** (Table 2). Under the same reaction conditions used for the preparation of derivatives **5**, phenyl isothiocyanate, *p*-methoxyphenyl isothiocyanate, or *p*-nitrophenyl isothiocyanate did not react with cyanoaziridine **1a** to yield compounds **6**. Moreover, the use of different bases, such as Et_3_N, pyridine, NaH, or Cs_2_CO_3_, as well as Lewis acids such as ZnCl_2_ or Sc (OTf)_3_, gave similar results: the formation of *N*-functionalized cyanoaziridines **6** was not observed and the starting compound **1a** was recovered instead.

In order to achieve the synthesis of new derivatives **6**, we decided to use a more reactive isothiocyanate derivative. Thus, functionalized isothiocyanates with an electron-withdrawing group, such as ethoxycarbonyl isothiocyanate, reacted with cyanoaziridines derived from phosphine oxide **1a** and **1b** in CH_2_Cl_2_ at −30 °C (Method A). Under these reaction conditions, compounds **6a** and **6b** were attained in 71% and 85% chemical yields, respectively (Table 2, entries 1 and 3). Increasing the reaction temperature to 25 °C (Method B) gave better yields and the *N*-functionalized cyanoaziridine **6a** was obtained in an 80% yield (Table 2, entry 2). Similarly, phosphonate-derived cyanoaziridine **1c** reacted with ethoxycarbonyl isothiocyanate in CH_2_Cl_2_ at room temperature to afford *N*-thiocarbamoyl cyanoaziridine **6c** (R = OEt, R^1^ = Me) in good yields (Table 2, entry 4).

Continuing with our interest in the synthesis of new 5-membered nitrogen containing heterocyclic compounds, we then explored the ring expansion of some *N*-carbamoyl cyanoaziridine derivatives **5**. To this end, and using the same reaction conditions as in the case of *N*-acyl cyanoaziridine **2**, *N*-arylcarbamoyl cyanoaziridines derived from phosphine oxide **5a** (R = R^2^ = Ph) and **5c** (R = Ph, R^2^ = *p*-MeC_6_H_4_) reacted with 0.2 equivalents of NaI at 60 °C in THF, allowing the preparation of oxazolines **7a** and **7c**, respectively (Table 3, entries 1 and 3). As evidenced by ^1^H and ^31^P NMR, oxazolines **7** were obtained as a mixture of two regioisomers **7** and **7′**, in a 66:34 ratio for **7a**, while a 65:35 ratio was observed for oxazoline **7c**. Oxazolines **7** were purified by flash-column chromatography, allowing the isolation of a single isomer, corresponding to the minor one in the case of **7a**+**7′a**. However, in the case of regioisomeric oxazolines **7c**+**7′c,** the separation of both regioisomers was not possible, and the same 65:35 ratio was obtained after purification by flash-column chromatography. We also tested the ring expansion of *N*-arylcarbamoyl cyanoaziridines derived from phosphonate **5b** and **5d** under the optimal conditions. For instance, phosphonate-derived oxazolines **7b** (R = OEt, R^2^ = Ph) and **7d** (R = OEt, R^2^ = *p*-MeC_6_H_4_) were obtained as regioisomeric mixtures after treatment with **5b** and **5d**, respectively, with 0.2 equivalents of NaI at 60 °C in THF (Table 3, entries 2 and 4). Conversely, the NaI catalyzed ring expansion of *N*-alkylcarbamoyl cyanoaziridines **5g** (R = Ph, R^1^ = Me, R^2^ = Et) and **5k** (R = Ph, R^1^ = Et, R^2^ = *^t^*Bu) to the corresponding oxazolines was not observed, and *N*-functionalized cyanoaziridines **5g** and **5k** were recovered instead.

A rational mechanism for the formation of oxazoline derivatives **7** can be explained via the initial aziridine ring opening in **5** by an indiscriminate iodide attack to either aziridine carbons C2 or C3. Subsequent ring closure by iodide displacement would afford a mixture of regioisomeric oxazolines **7** and **7′**. It seems reasonable to assume that the role of the stereoelectronic nature of *N*-substituents on the aziridine ring may affect the selectivity of these cyanooxazoline derivatives. Only one regioisomer was formed in the reaction of *N*-acylcyanoaziridine **2a** in the presence of NaI, suggesting the possibility that the *N*-acyl substituent could exert a neighboring group participation effect, although this does not take place in the case of the *N*-arylcarbamoyl group.

Several attempts have been carried out in the synthesis of thiazoline derivatives starting from aziridines. It is known that 2-substituted oxazolines or imidazolines can be prepared by the ring expansion of aziridines or benzoylated imidoyl aziridines, respectively [41,45,46], through the Heine reaction. For instance, aziridines undergo ring expansion reactions into oxazolines with Lewis acids [47] and, recently, based on these results, Tepe et al. [48] have described the isomerization of aziridines to oxazolines using BF_3_·OEt_2_. For this reason, we explored the ring expansion of functionalized *N*-thiocarbamoyl cyanoaziridine **6a**. Initially, we studied the aziridine ring opening under thermal conditions. Thus, *N*-thiocarbamoyl cyanoaziridine derived from phosphine oxide **6a** was heated in refluxing CHCl_3_. Under these conditions, no reaction was observed, and the unreacted starting substrate was recovered. Next, the Heine-type reaction was also studied under nucleophilic conditions by using NaI at 60 °C in THF, and as in the previous case, no satisfactory results were attained.

**Scheme 3:**
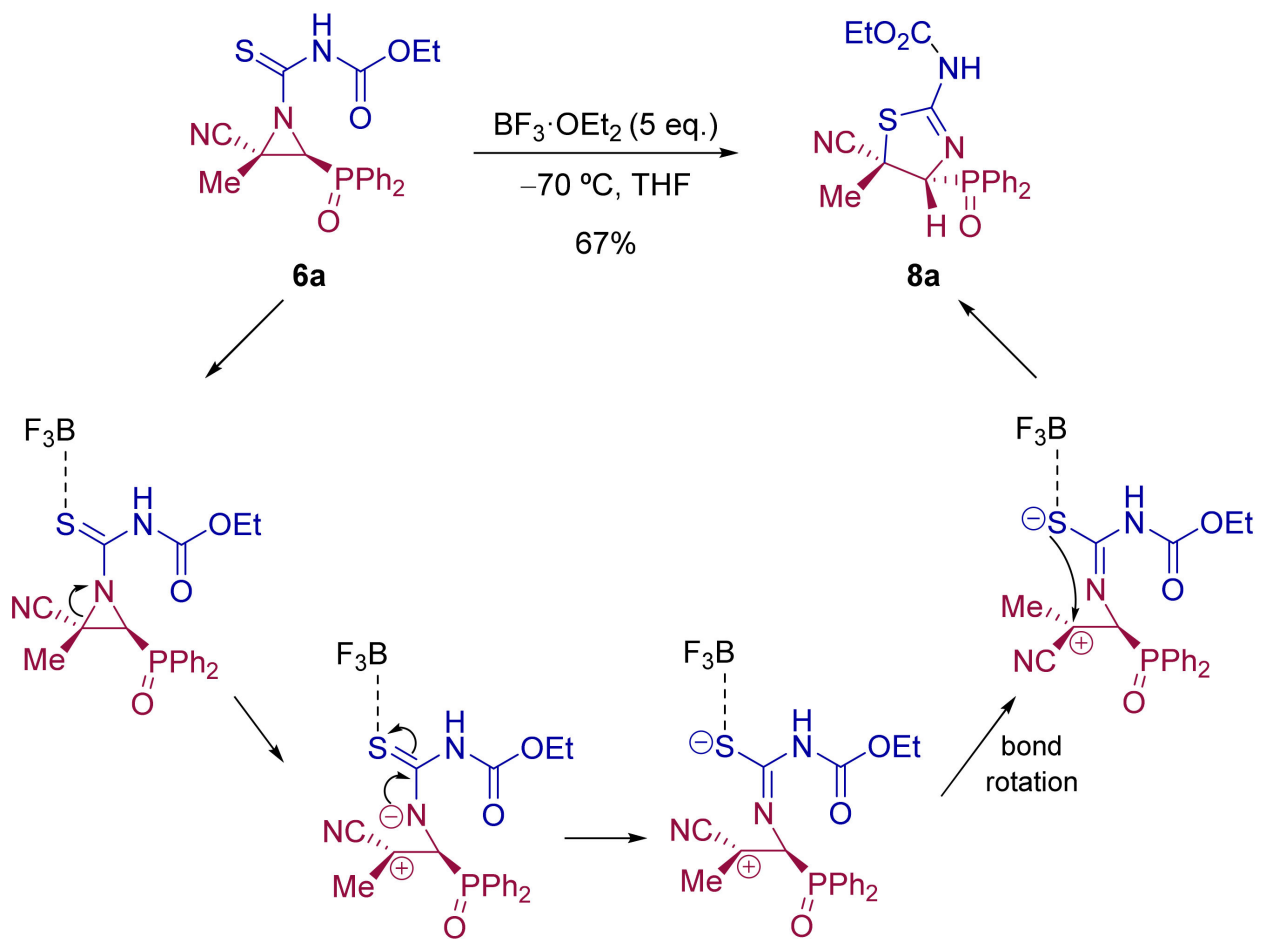
Stereospecific ring expansion of *N*-thiocarbamoyl cyanoaziridine **6a** to 2-aminothiazoline **8a**.

Likewise, the conversion of aziridine to thiazoline under mild acidic conditions was examined. *N*-Functionalized cyanoaziridine **6a** was treated with both Brønsted acids, such as *p*-toluenesulfonic acid (PTSA), and Lewis acids, such as ZnCl_2_ or BF_3_·OEt_2_. Only the use of BF_3_·OEt_2_ gave satisfactory results. Hence, when *N*-thiocarbamoyl cyanoaziridine **6a** reacted in the presence of 5 equivalents of BF_3_·OEt_2_ at −70 °C in THF, the formation of 2-aminothiazoline phosphine oxide **8a** was detected (Scheme 3). Spectroscopic data confirmed the isomerization of aziridine **6a** into 2-aminothiazoline **8a**. While the ^1^H NMR spectrum of **6a** showed a signal for the methyl group at *δ*_H_ = 2.0 ppm and the methine hydrogen resonated at *δ*_H_ = 3.8 ppm as a well-resolved doublet (^2^*J*_PH_ = 20 Hz), in 2-aminothiazolidine **8a** these signals appeared at lower fields: *δ*_H_ = 2.11 and 4.58 ppm as a singlet and a well-resolved doublet (^2^*J*_PH_ = 12.8 Hz), respectively.

**Figure 2:**
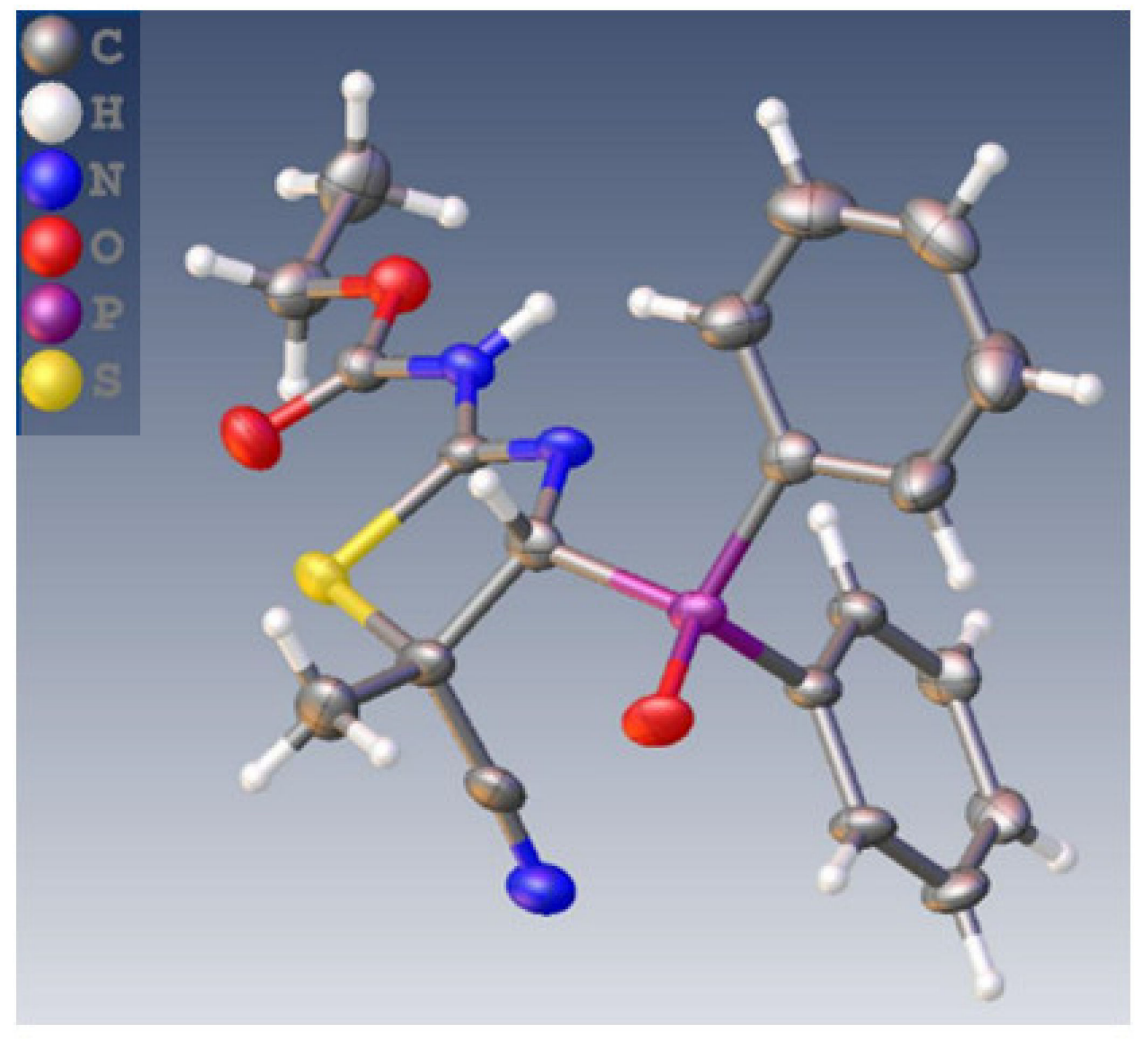
ORTEP diagram of compound **8a**.

Since it was not conclusively irrefutable that ^1^H and ^13^C NMR were assigned to the regio- and stereochemistry of compound **8a**, the X-ray diffraction analysis not only established the regiochemistry of compound **8a**, but also the *syn*-relationship between the cyano group at the C3 position and the phosphorus moiety at the C2 position of **8a** (Figure 2).

A reasonable mechanism that would explain the formation of **8a** is exemplified in Scheme 3. First, BF_3_·OEt_2_ would coordinate with the sulfur atom of cyanoaziridine **6a,** thus assisting the ring opening reaction through the N–C3 bond, with the concomitant generation of the most stable carbocation. The cationic intermediate coming from the aziridine with an *E*-stereochemistry would isomerize, and the ring closure would lead to 2-aminothiazoline **8a** as the only regio- and stereoisomer.

Continuing with our interest in the synthesis of new nitrogen-containing heterocyclic compounds, finally we examined the one pot reaction of cyanoaziridines **1** with isocyanates in order to obtain new oxazoline derivatives.

**Scheme 4:**
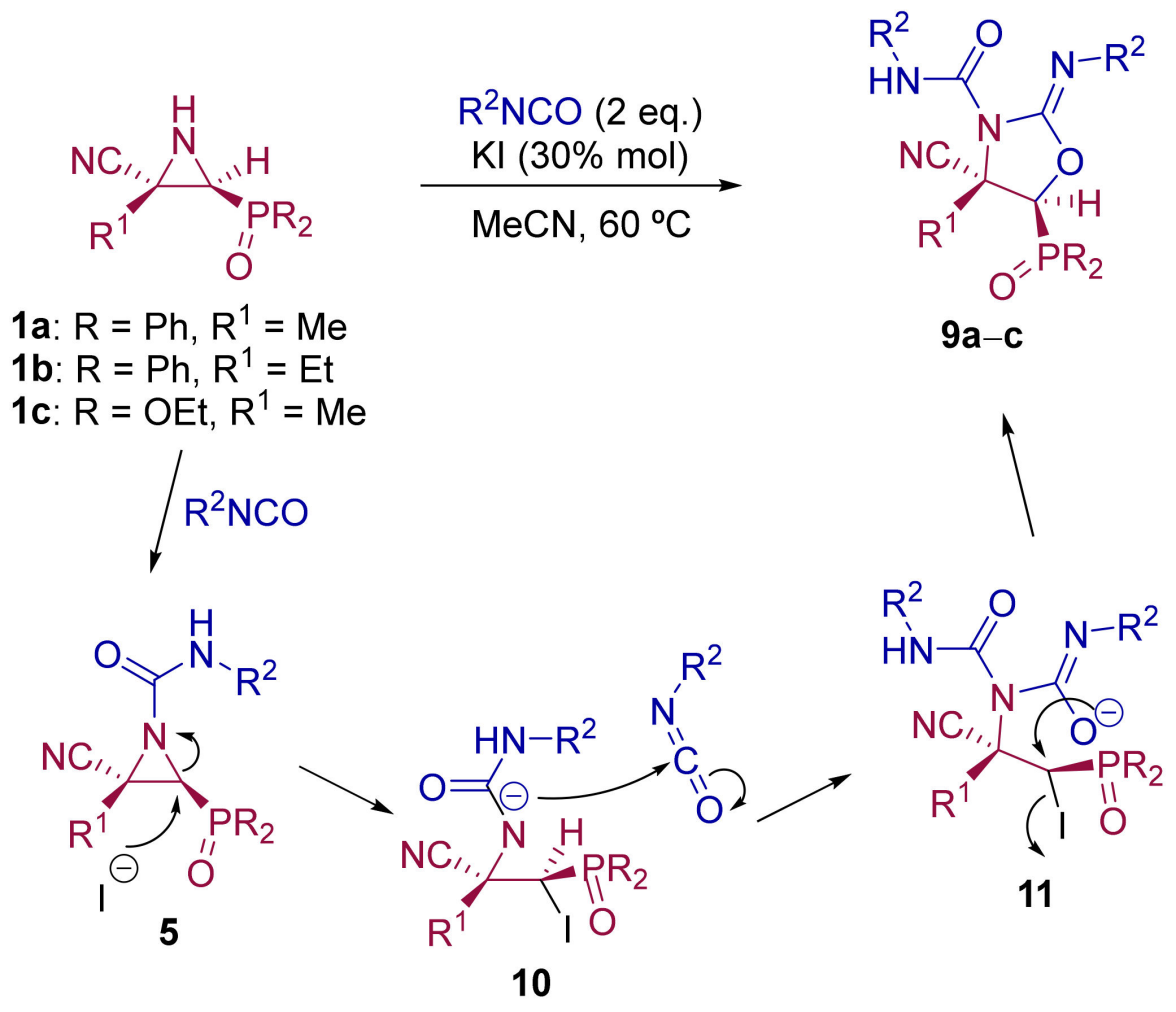
Synthesis of iminooxazolidines **9** through one pot reaction from cyanoaziridines **1**.

For this purpose, phosphorus subtituted cyanoaziridine **1b** (R = Ph, R^1^ = Et) reacted with phenyl isocyanate in acetonitrile at 60 °C, leading to the formation of iminooxazolidine **9a** in low yields (Scheme 4). The addition of 2 equivalents of isocyanate led to **9a** in moderate yields (45%), whereas, when the reaction was examined in the presence of KI (30% mol) using 2 equivalents of phenyl isocyanate in acetonitrile at 60 °C, the corresponding iminooxazolidine **9a** was obtained in a 62% yield (Scheme 4, Table 4, entry 1). Similarly, cyanoaziridine **1a** (R = Ph, R^1^ = Me) reacted with *p*-tolenesulfonyl isocyanate using the same reaction conditions, providing a 55% yield of iminooxazolidine **9b** (Scheme 4, Table 4, entry 2). This synthetic methodology was extended to the use of cyanoaziridines derived from phosphonate. Thus, **1c** (R = OEt, R^1^ = Me) reacted with phenyl isocyanate in the presence of KI in acetonitrile at 60 °C to give iminooxazolidine **9c** (Scheme 4, Table 4, entry 3).

A reliable mechanism for the formation of **9** would indicate the addition of an equivalent of isocyanate to cyanoaziridine **1** to obtain the corresponding *N*-carbamoyl cyanoaziridines **5** (Scheme 4). Then, the regiospecific attack of the iodide ion at the less substitute carbon atom (C2) in aziridines **5** would lead to the ring opening, affording intermediates **10**. The former intermediates would attack the carbon center of a second isocyanate equivalent followed by the ring closure to yield iminooxazolidines **9**.

### 2.2. Biological Results

The in vitro cytotoxicity of our novel *N*-functionalized cyanoaziridines **2**, **5** and **6** derived from phosphine oxide (R = Ph) and phosphonate (R = OEt), as well as the five-membered nitrogen-containing heterocycles **3a**, **7**, **8a** and **9** was evaluated by testing their antiproliferative activities against the human cancer cell line A549 (carcinomic human alveolar basal epithelial cells). In order to evaluate the growth inhibition, a cell counting kit (CCK-8) assay was applied. Cell proliferation inhibitory activities as IC_50_ values for all the synthesized compounds and chemotherapeutic doxorubicin (DOX) are displayed in Table 5. Likewise, healthy lung cells, such as MRC-5 non-malignant lung fibroblasts were tested to study the selective cytotoxicity [49]. We first examined the nitrogen-substitution effect of the corresponding cyanoaziridines into their cytotoxicity against A549 cell lines. The best result was observed for *N*-acylated cyanoaziridine **2a** derived from phosphine oxide with an IC_50_ value of 22.9 ± 1.9 μM (Table 5, entry 2). However, *N*-acylated cyanoaziridines derived from phosphine oxide **2b** and phosphonate **2c** (Table 5, entries 3 and 4), as well as *N*-carbamoyl cyanoaziridines **5a**–**k** (Table 5, entries 6–16) and *N*-thiocarbamoyl cyanoaziridines **6a**–**c** (Table 5, entries 17–19) did not exhibit any toxicity toward the A549 cell line.

Concerning the new 5-membered nitrogen-containing heterocycles derived from the ring expansion of *N*-functionalized cyanoaziridines against the A549 cell line in vitro, oxazoline derivative **3a** showed a IC_50_ value of 19.7 ± 2.8 μM (Table 5, entry 5). Conversely, neither the regioisomeric oxazolines **7**+**7′** (Table 5, entries 20–23) nor the 2-aminothiazoline derivative **8a** (Table 5, entry 24) displayed any cytotoxicity against the same cell line. Finally, we studied the cytotoxicity effect of iminooxazolidines **9a**–**c** against A549 cell lines. For instance, IC_50_ values between 6.2 ± 0.7 and 16.4 ± 1.5 μM were observed, with iminooxazolidine **9c** (Table 5, entry 27) as the most effective compound with an IC_50_ value of 6.2 ± 0.7 μM. It appears rational to presume that the observed cytotoxic activity in imonooxazolidines **9**, which was not observed in oxazolines **7**, could be due to the presence of an amide group at the N–3 of the oxazoline ring.

Furthermore, MRC-5 non-malignant lung fibroblasts were tested to study the selective toxicity [49], and none of the synthesized phosphorus substituted *N*-functionalized cyanoaziridines, 5-membered nitrogen-containing heterocycles, or doxorubicin exhibited any toxicity toward the MRC-5 cell line (see Table 5).

## 3. Materials and Methods

### 3.1. Chemistry

#### 3.1.1. General Experimental Information

Solvents for extraction and chromatography were of a technical grade. All solvents used in reactions were freshly distilled and dried over molecular sieves 4 Å before use. All other solvents and reagents were obtained from commercial sources (Sigma-Aldrich, Spain) and recrystallized or distilled as necessary or were used without further purification. All reactions were performed under an atmosphere of dry nitrogen. Melting points were determined using the Büchi Melting Point B-540 apparatus and were uncorrected. IR spectra were measured on a Nicolet iS10 Thermo Fisher Scientific spectrometer (Thermo Scientific Inc., Waltham, MA, USA) as neat solids. Absorbance frequencies are given at maximum of intensity in cm^−1^. High-resolution mass spectra (HRMS) were measured on an Agilent 6530 Accurate-Mass QTOF LC/MS (Santa Clara, CA, USA) by a positive-ion electrospray ionization (ESI) method with a time-of-flight Q-TOF system. Data are reported in the form *m*/*z* (intensity relative to base = 100). ^1^H (300, 400 MHz), ^13^C (75, 100 MHz), and ^31^P NMR (120, 160 MHz) spectra were recorded on Varian Unity Plus (Varian Inc., NMR Systems, Palo Alto, CA, USA) or on Bruker Avance 400 (Bruker BioSpin GmbH, Rheinstetten, Germany) spectrometers, respectively, in CDCl_3_ at 25 °C. Chemical shifts (*δ*_H_) are reported in parts per million (ppm) with the internal chloroform signal at 7.24 ppm as the standard for ^1^H NMR. Chemical shifts (*δ*_C_ and *δ*_P_) are reported in parts per million (ppm) with the internal chloroform signal at 77.0 ppm as the standard for ^13^C NMR, or the external H_3_PO_4_ (50%) signal at 0.0 ppm as the standard for ^31^P NMR. All coupling constants (*J*) values are given in Hz. ^13^C NMR spectra were recorded in a broadband decoupled mode from hydrogen nuclei. Distortionless Enhanced Polarization Transfer (DEPT) supported peak assignments for ^13^C NMR. The data are reported as s *=* singlet, d *=* doublet, t *=* triplet, q *=* quartet, m *=* multiplet, dd = double doublet, bs *=* broad singlet. Chromatographic purification was performed as flash chromatography using commercial grades of silica gel finer than 230 mesh with pressure. Analytical thin layer chromatography (TLC) was performed on precoated Merck silica gel 60 F_254_ TLC aluminium plates, and spot visualized with UV light or permanganate stain. Cyanoaziridines **1** were prepared according to procedures in the literature [16].

#### 3.1.2. Experimental Procedure and Characterization Data for Compounds **2**–**9**

##### General Procedure and Spectral Data for The Addition of 3,5-Dinitrobenzoyl Chloride to Functionalized Cyanoaziridines

3,5-Dinitrobenzoyl chloride (1.4 g, 6 mmol, 1.2 eq) and Et_3_N (2.8 mL, 20 mmol, 4 eq) were added to a 0 °C solution of cyanoaziridine (5 mmol, 1 eq) in CH_2_Cl_2_ (25 mL). The reaction mixture was stirred at 0 °C until TLC showed the disappearance of the starting cyanoaziridine. The crude product was washed three times with a saturated NaCl solution (15 mL) and water (15 mL) and extracted with CH_2_Cl_2_ (15 mL). The organic layers were dried over anhydrous MgSO_4_, filtered and concentrated to dryness in vacuum conditions, and the resulting residue was purified by crystallization from Et_2_O/pentane or washed with pentane.

(*E*)-(2S*,3S*)-1-(3,5-Dinitrobenzoyl)-3-(diphenylphosphoryl)-2-methylaziridine-2-carbonitrile (**2a**), (1.97 g, 83%) was obtained as a grey solid from cyanoaziridine **1a** (1.41 g, 5 mmol) after 24 h at 0 °C as described in the general procedure. The crude product was purified by crystallization from Et_2_O/pentane (50:50) to give the title compound **2a**; mp 120–122 °C; IR (neat) *v*_max_ 3067, 2948, 2237, 1699, 1546, 1346, 1252, 1210, 1152 cm^−1^; ^1^H NMR (300 MHz, CDCl_3_) *δ* 9.23 (t, ^4^*J*_HH_ = 2.0 Hz, 1H, Ar*H*), 9.08 (d, ^4^*J*_HH_ = 2.0 Hz, 2H, Ar*H*), 7.85–7.48 (m, 10H, Ar*H*), 3.86 (d, ^2^*J*_PH_ = 20.5 Hz, 1H, C*H*-P), 2.17 (s, 3H, C*H_3_*) ppm; ^13^C {^1^H} NMR (75 MHz, CDCl_3_) *δ* 171.8 (d, ^3^*J*_PC_ = 3.0 Hz, C=O), 149.0 (C_quat_), 135.1(C_quat_), 133.5, 133.5, 133.4, 133.3, 131.3, 131.1, 131.1, 131.0, 129.7, 129.5, 129.4, 129.2, 129.0 (C_Ar_),120.4 (C_quat_), 116.6 (CN), 43.4 (d, ^1^*J*_PC_ = 90.7 Hz, CH-P), 37.0 (d, ^2^*J*_PC_ = 2.5 Hz, C_quat_), 16.9 (CH_3_) ppm; ^31^P NMR (120 MHz, CDCl_3_) *δ* 21.9 ppm; ESI-HRMS (CI) *m*/*z* calculated for C_23_H_18_N_4_O_6_P ([M + H]^+^) 477.0964 found 477.0971 (See Supplementary Materials).


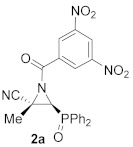


(*E*)-(2S*,3S*)-1-(3,5-Dinitrobenzoyl)-3-(diphenylphosphoryl)-2-ethylaziridine-2-carbonitrile (**2b**), (1.95 g, 80%) was obtained as a grey solid from cyanoaziridine **1b** (1.48 g, 5 mmol) after 24 h at 0 °C as described in the general procedure. The crude product was purified by crystallization from Et_2_O/pentane (50:50) to give the title compound **2b**; mp 201–203 °C; IR (neat) *v*_max_ 3101, 2884, 2237, 1710, 1630, 1544, 1460, 1441, 1344, 1294, 1202 1147, 1122 cm^−1^; ^1^H NMR (300 MHz, CDCl_3_) *δ* 9.24 (t, ^4^*J*_HH_ = 2.1 Hz, 1H, Ar*H*), 9.11 (d, ^4^*J*_HH_ = 2.1 Hz, 2H, Ar*H*), 7.90–7.47 (m, 10H, Ar*H*), 3.90 (d, ^2^*J*_PH_ = 20.4 Hz, 1H, C*H*-P), 2.61–2.46 (m, 2H, C*H*_2_), 1.08 (t, ^3^*J*_HH_ = 7.4 Hz, 3H, C*H_3_*) ppm; ^13^C {^1^H} NMR (75 MHz, CDCl_3_) *δ* 172.4 (d, ^3^*J*_PC_ = 3.3 Hz, C=O), 148.9 (C_quat_), 135.1 (C_quat_), 133.5, 133.4, 133.3, 133.2, 131.2, 131.2, 131.1, 131.0, 129.6, 129.5, 129.3, 129.2, 129.1, 123.4 (C_Ar_), 115.6 (CN), 43.6 (d, ^1^*J*_PC_ = 90.6 Hz, CH-P), 43.2 (d, ^2^*J*_PC_ = 2.6 Hz, C_quat_), 24.2 (CH_2_), 10.5 (CH_3_) ppm; ^31^P NMR (120 MHz, CDCl_3_) *δ* 21.4 ppm; ESI-HRMS (CI) *m*/*z* calculated for C_24_H_20_N_4_O_6_P ([M + H]^+^) 491.1120 found 491.1135.


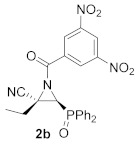


Diethyl (*E*)-[(2S*,3S*)-3-cyano-1-(3,5-dinitrobenzoyl)-3-methylaziridin-2-yl]phosphonate (**2c**), (1.92 g, 93%) was obtained as a brown oil from cyanoaziridine **1c** (1.09 g, 5 mmol) after 24 h at 0 °C as described in the general procedure. The crude product was washed with pentane to give the title compound **2c**; Rf: 0.5 (AcOEt); IR (neat) *v*_max_ 3112, 2984, 2246, 1710, 1627, 1552, 1344, 1294, 1255, 1041, 1022 cm^−1^; ^1^H NMR (300 MHz, CDCl_3_) *δ* 9.25 (t, ^4^*J*_HH_ = 2.1 Hz, 1H, Ar*H*), 9.05 (d, ^4^*J*_HH_ = 2.1 Hz, 2H, Ar*H*), 4.30–4.17 (m, 4H, OC*H_2_*CH_3_), 3.37 (d, ^2^*J*_PH_ = 11.9 Hz, 1H, C*H*-P), 2.09 (s, 3H, C*H_3_*), 1.42–1.34 (m, 6H, OCH_2_C*H*_3_) ppm; ^13^C {^1^H} NMR (75 MHz, CDCl_3_) *δ* 171.4 (d, ^3^*J*_PC_ = 5.0 Hz, C=O), 149.1 (C_quat_), 134.8 (C_quat_), 128.9, 123.4 (C_Ar_), 116.3 (^3^*J*_PC_ = 2.1 Hz, CN), 64.2 (d, ^2^*J*_PC_ = 6.1 Hz, OCH_2_), 63.7 (d, ^2^*J*_PC_ = 6.5 Hz, OCH_2_) 40.4 (d, ^1^*J*_PC_ = 202.3 Hz, CH-P), 35.8 (d, ^2^*J*_PC_ = 2.7 Hz, C_quat_), 17.6 (CH_3_), 16.5 (d, ^3^*J*_PC_ = 5.9 Hz, OCH_2_*C*H_3_), 16.4 (d, ^3^*J*_PC_ = 5.9 Hz, OCH_2_*C*H_3_) ppm; ^31^P NMR (120 MHz, CDCl_3_) *δ* 12.3 ppm; ESI-HRMS (CI) *m*/*z* calculated for C_15_H_18_N_4_O_8_P ([M + H]^+^) 413.0862 found 413.0857.


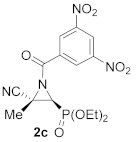


##### General Procedure and Spectral Data for Compound **3a**

To a stirred solution of *N*-functionalized cyanoaziridine **2a** (5 mmol, 1 eq) in THF (15 mL), NaI (0.02 g, 1 mmol, 0.2 eq) was added dropwise. The mixture was heated at 60 °C for 24 h until TLC showed the disappearance of the starting cyanoaziridine. The reaction mixture was concentrated to dryness in vacuum conditions to remove THF. The crude product was washed three times with water (15 mL) and extracted with CH_2_Cl_2_ (15 mL). The organic layer was dried over anhydrous MgSO_4_, filtered, and concentrated to dryness in vacuum conditions. The crude product was purified by flash-column chromatography.

(*E*)-(4S*,5S*)-2-(3,5-Dinitrophenyl)-5-(diphenylphosphoryl)-4-methyl-4,5-dihydrooxazole-4-carbonitrile (**3a**), (1.56 g, 65%) was obtained as a yellow solid from cyanoaziridine **2a** (2.38 g, 5 mmol) after 24 h of heating in THF as described in the general procedure. The crude product was purified by flash-column chromatography (SiO_2_, AcOEt/hexane 25:75) to give the title compound **3a**; mp 129–131 °C; IR (neat) *v*_max_ 3103, 2934, 2243, 1655, 1546, 1438, 1352, 1197, 1119 cm^−1^; ^1^H NMR (300 MHz, CDCl_3_) *δ* 9.19 (t, ^4^*J*_HH_ = 2.1 Hz, 1H, Ar*H*), 8.99 (d, ^4^*J*_HH_ = 2.1 Hz, 2H, Ar*H*), 7.98–7.52 (m, 10H, Ar*H*), 5.60 (d, ^2^*J*_PH_ = 6.7 Hz, 1H, C*H*-P), 1.80 (s, 3H, C*H_3_*) ppm; ^13^C {^1^H} NMR (75 MHz, CDCl_3_) *δ* 161.7 (d, ^3^*J*_PC_ = 5.6 Hz, C_quat_), 148.8, 133.8, 133.8, 133.4, 133.3, 131.4, 131.3, 131.2, 131.1, 129.9, 129.7, 129.4, 129.3, 128.8, 122.2 (C_Ar_),119.8 (d, ^3^*J*_PC_ = 8.9 Hz, CN), 108.1 (C_quat_), 83.2 (d, ^1^*J*_PC_ = 76.7 Hz, CH-P), 68.3 (d, ^2^*J*_PC_ = 2.0 Hz, C_quat_), 23.0 (d, ^3^*J*_PC_ = 5.2 Hz, CH_3_) ppm; ^31^P NMR (120 MHz, CDCl_3_) *δ* 22.5 ppm; ESI-HRMS (CI) *m*/*z* calculated for C_23_H_18_N_4_O_6_P ([M + H]^+^) 477.0964 found 477.0965.


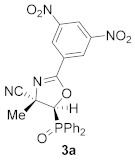


##### General Procedures and Spectral Data for The Addition of Isocyanates to Functionalized Cyanoaziridines **1**

Method A. To a 0 °C solution of cyanoaziridine (5 mmol, 1 eq) in CH_2_Cl_2_ (25 mL) the corresponding isocyanate (6 mmol, 1.2 eq) was added dropwise. The reaction mixture was allowed to reach room temperature and stirred for 6–24 h. The crude products were concentrated to dryness in vacuum conditions and were purified by crystallization. Method B. To a 0 °C solution of cyanoaziridine (5 mmol, 1 eq) in CH_2_Cl_2_ (25 mL) phenyl isocyanate (15 mmol, 3 eq) and Sc (OTf)_3_ (0.49 g, 1 mmol, 0.2 eq) were added dropwise. The reaction mixture was stirred at 0 °C for 5 h until TLC showed the disappearance of the starting cyanoaziridine. The reaction mixture was washed with water (3 × 15 mL) and extracted with CH_2_Cl_2_ (15 mL). The organic layer was dried over anhydrous MgSO_4_, filtered, and concentrated to dryness in vacuum conditions. The crude product was purified by crystallization from Et_2_O. Method C. To a solution of cyanoaziridine (5 mmol, 1 eq) in CH_2_Cl_2_ (25 mL) the corresponding aliphatic isocyanate (10 mmol, 2 eq) and ZnCl_2_ (0.85 g, 6.25 mmol, 1.25 eq) were added dropwise. The reaction mixture was stirred at room temperature for 5–48 h until TLC showed the disappearance of the starting cyanoaziridine. The reaction mixture was washed with saturated NH_4_Cl (1 × 15 mL) and water (3 × 15 mL) and extracted with CH_2_Cl_2_ (15 mL). The organic layers were dried over anhydrous MgSO_4_, filtered, and concentrated to dryness in vacuum conditions. The crude product was purified by crystallization.

(*E*)-(2S*,3S*)-2-Cyano-3-(diphenylphosphoryl)-2-methyl-*N*-phenylaziridine-1-carboxamide (**5a**), (1.27 g, 63%) was obtained as a white solid from cyanoaziridine **1a** (1.41 g, 5 mmol) and phenylisocyanate (0.65 mL, 6 mmol, 1.2 eq) as described in the general procedure (method A). The crude product was purified by crystallization from Et_2_O to give the title compound **5a**. (1.42 g, 71%) obtained as an orange pale solid from cyanoaziridine **1a** (1.41 g, 5 mmol), phenylisocyanate (0.65 mL, 6 mmol, 1.2 eq) and Sc(OTf)_3_ (1 mmol, 0.49 g) as described in the general procedure (method B). The crude product was purified by crystallization from Et_2_O to give the title compound **5a**; mp 179–181 °C; IR (neat) *v*_max_ 3220, 3053, 2926, 2256, 1710, 1596, 1544, 1435, 1252, 1202, 1127 cm^−1^; ^1^H NMR (400 MHz, CDCl_3_) *δ* 9.11 (bs, 1H, N*H*), 7.88–7.07 (m, 15H, Ar*H*), 3.76 (d, ^2^*J*_PH_ = 23.1 Hz, 1H, C*H*-P), 1.92 (s, 3H, C*H_3_*) ppm; ^13^C {^1^H} NMR (75 MHz, CDCl_3_) *δ* 157.2 (C=O), 137.8 (C_quat_), 133.1, 133.1, 133.1, 133.0, 131.6, 131.4, 131.2, 131.2, 131.0, 130.1, 130.0, 129.5, 129.4, 129.3, 129.2, 129.1, 124.6, 120.1 (C_Ar_), 117.1 (CN), 41.3 (d, ^1^*J*_PC_ = 102.1 Hz, CH-P), 37.4 (C_quat_), 17.9 (CH_3_) ppm; ^31^P NMR (120 MHz, CDCl_3_) *δ* 23.4 ppm; ESI-HRMS (CI) *m*/*z* calculated for C_23_H_21_N_3_O_2_P ([M + H]^+^) 402.1371 found 402.1374.


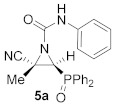


Diethyl (*E*)-[(2S^*^,3S^*^)-3-cyano-3-methyl-1-(phenylcarbamoyl)aziridin-2-yl]phosphonate (**5b**), (1.65 g, 98%) was obtained as a white solid from cyanoaziridine **1c** (1.09 g, 5 mmol) and phenylisocyanate (0.65 mL, 6 mmol, 1.2 eq) as described in the general procedure (method A). The crude product was purified by crystallization from CH_2_Cl_2_/pentane to give the title compound **5b**; mp 137–139 °C; IR (neat) *v*_max_ 3253, 3065, 2984, 2240, 1716, 1607, 1544, 1499, 1444, 1249, 1044, 1024 cm^−1^; ^1^H NMR (300 MHz, CDCl_3_) *δ* 8.30 (bs, 1H, N*H*), 7.48–7.09 (m, 5H, Ar*H*), 4.25–4.15 (m, 4H, OC*H_2_*CH_3_), 3.29 (d, ^2^*J*_PH_ = 13.6 Hz, 1H, C*H*-P), 1.92 (s, 3H, C*H_3_*), 1.40–1.32 (m, 6H, OCH_2_C*H*_3_) ppm; ^13^C {^1^H} NMR (75 MHz, CDCl_3_) *δ* 156.6 (C=O), 137.3 (C_quat_), 129.2, 124.8, 120.0 (C_Ar_), 116.9 (CN), 63.9 (d, ^2^*J*_PC_ = 5.4 Hz, OCH_2_), 63.3 (d, ^2^*J*_PC_ = 6.0 Hz, OCH_2_) 38.7 (d, ^1^*J*_PC_ = 207.4 Hz, CH-P), 36.0 (C_quat_), 18.2 (CH_3_), 16.6 (d, ^3^*J*_PC_ = 4.9 Hz, OCH_2_*C*H_3_), 16.5 (d, ^3^*J*_PC_ = 4.7 Hz, OCH_2_*C*H_3_) ppm; ^31^P NMR (120 MHz, CDCl_3_) *δ* 14.6 ppm; ESI-HRMS (CI) *m*/*z* calculated for C_15_H_21_N_3_O_4_P ([M + H]^+^) 338.1270 found 338.1264.


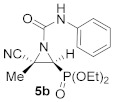


(*E*)-(2S*,3S*)-2-Cyano-3-(diphenylphosphoryl)-2-methyl-*N*-(*p*-tolyl) aziridine-1-carboxamide (**5c**), (1.70 g, 82%) was obtained as a yellow solid from cyanoaziridine **1a** (1.41 g, 5 mmol) and *p*-tolyl isocyanate (0.76 mL, 6 mmol, 1.2 eq) as described in the general procedure (method A). The crude product was purified by crystallization from Et_2_O to give the title compound **5c**; mp 193–195 °C; IR (neat) *v*_max_ 3237, 3056, 2917, 2254, 1710, 1599, 1546, 1539, 1408, 1249, 1197 cm^−1^; ^1^H NMR (400 MHz, CDCl_3_) *δ* 9.17 (bs, 1H, N*H*), 7.86–7.06 (m, 14H, Ar*H*), 3.76 (d, ^2^*J*_PH_ = 23.0 Hz, 1H, C*H*-P), 2.28 (s, 3H, C*H_3_*), 1.92 (s, 3H, C*H_3_*) ppm; ^13^C {^1^H} NMR (75 MHz, CDCl_3_) *δ* 157.1 (d, ^3^*J*_PC_ = 4.2 Hz, C=O), 135.2 (C_quat_), 134.2 (C_quat_), 133.0, 133.0, 131.3, 131.2, 131.1, 131.0, 129.5, 129.4, 129.3, 129.1, 120.2, 120.0, (C_Ar_), 117.1 (CN), 41.2 (d, ^1^*J*_PC_ = 100.5 Hz, CH-P), 37.3 (d, ^2^*J*_PC_ = 3.1 Hz, C_quat_), 21.0 (CH_3_), 17.9 (CH_3_) ppm; ^31^P NMR (120 MHz, CDCl_3_) *δ* 23.6 ppm; ESI-HRMS (CI) *m*/*z* calculated for C_24_H_23_N_3_O_2_P ([M + H]^+^) 416.1528 found 416.1532.


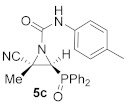


Diethyl (*E*)-[(2S*,3S*)-3-cyano-3-methyl-1-(*p*-tolylcarbamoyl)aziridin-2-yl]phosphonate (**5d**), (1.40 g, 80%) was obtained as a pale yellow solid from cyanoaziridine **1c** (1.09 g, 5 mmol) and *p*-tolyl isocyanate (0.76 mL, 6 mmol, 1.2 eq) as described in the general procedure (method A). The crude product was purified by crystallization from CH_2_Cl_2_/pentane to give the title compound **5d**; mp 140–142 °C; IR (neat) *v*_max_ 3256, 3040, 2987, 2240, 1710, 1607, 1538, 1444, 1516, 1321, 1247, 1039 cm^−1^; ^1^H NMR (300 MHz, CDCl_3_) *δ* 8.15 (bs, 1H, N*H*), 7.33 (d, ^3^*J*_HH_ = 7.9 Hz, 2H, Ar*H*), 7.09 (d, ^3^*J*_HH_ = 7.9 Hz, 2H, Ar*H*), 4.24–4.14 (m, 4H, OC*H_2_*CH_3_), 3.28 (d, ^2^*J*_PH_ = 13.5 Hz, 1H, C*H*-P), 2.28 (s, 3H, C*H_3_*), 1.91 (s, 3H, C*H_3_*), 1.39–1.32 (m, 6H, OCH_2_C*H*_3_) ppm; ^13^C {^1^H} NMR (75 MHz, CDCl_3_) *δ* 156.6 (d, ^3^*J*_PC_ = 5.9 Hz, C=O), 134.9 (C_quat_), 134.3 (C_quat_), 129.5, 120.1 (*C*_Ar_), 116.9 (CN), 63.9 (d, ^2^*J*_PC_ = 6.2 Hz, OCH_2_), 63.2 (d, ^2^*J*_PC_ = 6.5 Hz, OCH_2_), 38.4 (d, ^1^*J*_PC_ = 206.8 Hz, CH-P), 36.0 (d, ^2^*J*_PC_ = 2.2 Hz, C_quat_), 20.9 (CH_3_), 18.1 (CH_3_), 16.5 (d, ^3^*J*_PC_ = 6.0 Hz, OCH_2_*C*H_3_), 16.4 (d, ^3^*J*_PC_ = 6.0 Hz, OCH_2_*C*H_3_) ppm; ^31^P NMR (120 MHz, CDCl_3_) *δ* 14.8 ppm; ESI-HRMS (CI) *m*/*z* calculated for C_16_H_23_N_3_O_4_P ([M + H]^+^) 352.1426 found 352.1419.


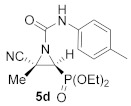


(*E*)-(2S*,3S*)-2-Cyano-3-(diphenylphosphoryl)-2-ethyl-*N*-tosylaziridine-1-carboxamide (**5e**), (2.12 g, 86%) was obtained as a white solid from cyanoaziridine **1b** (1.48 g, 5 mmol) and *p*-toluenesulfonyl isocyanate (0.92 mL, 6 mmol, 1.2 eq) as described in the general procedure (method A). The crude product was purified by crystallization from Et_2_O to give the title compound **5e**; mp 201–203 °C; IR (neat) *v*_max_ 3257, 2931, 2245, 1743, 1605, 1444, 1360, 1242, 1124, 1094 cm^−1^; ^1^H NMR (400 MHz, MeOD) *δ* 7.92–7.38 (m, 14H, Ar*H*), 3.39 (d, ^2^*J*_PH_ = 23.4 Hz, 1H, C*H*-P), 1.97 (m, 2H, C*H*_2_), 2.44 (s, 3H, C*H_3_*), 1.02 (t, ^3^*J*_HH_ = 7.4 Hz, 3H, C*H_3_*) ppm; ^13^C {^1^H} NMR (75 MHz, MeOD) *δ* 153.5 (C=O), 146.0 (C_quat_), 137.8 (C_quat_), 134.2, 134.2, 133.9, 133.9, 132.2, 132.1, 132.1, 132.0, 130.5, 130.4, 130.3, 130.2, 130.0, 129.0 (C_Ar_), 120.4 (CN), 39.5 (d, ^1^*J*_PC_ = 101.6 Hz, CH-P), 36.6 (C_quat_), 25.2 (CH_2_), 21.5 (CH_3_), 11.1 (CH_3_) ppm; ^31^P NMR (120 MHz, MeOD) *δ* 27.2 ppm; ESI-HRMS (CI) *m*/*z* calculated for C_25_H_25_N_3_O_4_PS ([M + H]^+^) 494.1303 found 494.1292.


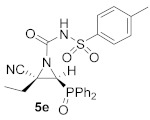


Diethyl (*E*)-[(2S*,3S*)-3-cyano-3-methyl-1-(tosylcarbamoyl)aziridin-2-yl]phosphonate (**5f**), (1.64 g, 79%) was obtained as a waxy white solid from cyanoaziridine **1c** (1.09 g, 5 mmol) and *p*-toluenesulfonyl isocyanate (0.92 mL, 6 mmol, 1.2 eq) as described in the general procedure (method A). The crude product was purified by crystallization from CH_2_Cl_2_/pentane to give the title compound **5f**; Rf: 0.4 (AcOEt). IR (neat) *v*_max_ 3248, 3092, 2992, 2237, 1738, 1649, 1596, 1446, 1335, 1247, 1160, 1047, 1027 cm^−1^; ^1^H NMR (300 MHz, CDCl_3_) *δ* 10.50 (bs, 1H, N*H*), 7.89–7.78 (m, 4H, Ar*H*), 4.24–4.10 (m, 4H, OC*H*_2_CH_3_), 3.04 (d, ^2^*J*_PH_ = 13.2 Hz, 1H, C*H*-P), 2.38 (s, 3H, C*H*_3_), 1.83 (s, 3H, C*H*_3_), 1.37–1.28 (m, 6H, OCH_2_C*H_3_*) ppm; ^13^C {^1^H} NMR (75 MHz, CDCl_3_) *δ* 155.3 (d, ^3^*J*_PC_ = 6.6 Hz, C=O), 145.3 (C_quat_), 135.1 (C_quat_), 129.8, 129.7, 128.6, 128.1, 126.5 (C_Ar_), 116.2 (CN), 64.3 (d, ^2^*J*_PC_ = 6.0 Hz, OCH_2_), 63.8 (d, ^2^*J*_PC_ = 6.4 Hz, OCH_2_), 38.9 (d, ^1^*J*_PC_ = 206.6 Hz, CH-P), 36.3 (d, ^2^*J*_PC_ = 3.0 Hz, C_quat_), 21.8 (CH_3_), 17.7 (CH_3_), 16.5 (d, ^3^*J*_PC_ = 6.3 Hz, OCH_2_*C*H_3_), 16.4 (d, ^3^*J*_PC_ = 6.2 Hz, OCH_2_*C*H_3_) ppm; ^31^P NMR (120 MHz) *δ* 13.3 ppm; ESI-HRMS (CI) *m*/*z* calculated for C_16_H_23_N_3_O_6_PS ([M + H]^+^) 416.1045 found 416.1038.


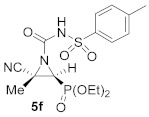


(*E*)-(2S*,3S*)-2-Cyano-3-(diphenylphosphoryl)-*N*-ethyl-2-methylaziridine-1-carboxamide (**5g**), (1.51 g, 86%) was obtained as a pale pink solid from cyanoaziridine **1a** (1.41 g, 5 mmol) and ethyl isocyanate (0.79 mL, 10 mmol, 2 eq) as described in the general procedure (method C). The crude product was purified by crystallization from Et_2_O/pentane 50:50 to give the title compound **5g**; mp 182–184 °C; IR (neat) *v*_max_ 3253, 3053, 2976, 2240, 1702, 1544, 1438, 1283, 1258, 1191, 1124 cm^−1^; ^1^H NMR (400 MHz, CDCl_3_) *δ* 7.84–7.46 (m, 10H, Ar*H*), 5.98 (t, ^3^*J*_HH_ = 5.9 Hz, 1H, N*H*), 3.65 (d, ^2^*J*_PH_ = 22.1 Hz, 1H, C*H*-P), 3.37–3.18 (m, 2H, NHC*H_2_*CH_3_), 1.85 (s, 3H, CH_3_)_,_ 1.13 (t, ^3^*J*_HH_ = 7.3 Hz, NHCH_2_C*H*_3_) ppm; ^13^C {^1^H} NMR (100 MHz, CDCl_3_) *δ* 159.1 (d, ^3^*J*_PC_ = 4.6 Hz, C=O), 133.0, 132.9, 132.8, 132.8, 131.6, 131.4, 131.3, 131.1, 131.1, 130.6, 130.3, 129.3, 129.2, 129.1, 129.0 (C_Ar_), 117.1 (CN), 41.6 (d, ^1^*J*_PC_ = 100.1 Hz, CH-P), 36.7 (d, ^2^*J*_PC_ = 3.4 Hz, C_quat_), 36.4 (NH*C*H_2_CH_3_), 18.1 (CH_3_), 15.1 (NHCH_2_*C*H_3_) ppm; ^31^P NMR (120 MHz, CDCl_3_) *δ* 23.1 ppm; ESI-HRMS (CI) *m*/*z* calculated for C_19_H_21_N_3_O_2_P ([M + H]^+^) 354.1371 found 354.1372.


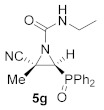


Diethyl (*E*)-[(2S*,3S*)-3-cyano-1-(ethylcarbamoyl)-3-methylaziridin-2-yl]phosphonate (**5h**), (0.85 g, 59%) was obtained as a waxy solid from cyanoaziridine **1c** (1.09 g, 5 mmol) and ethyl isocyanate (0.79 mL, 10 mmol, 2 eq) as described in the general procedure (method C). The crude product was purified by crystallization from Et_2_O/pentane 50:50 to give the title compound **5h**; Rf: 0.3 (AcOEt); IR (neat) *v*_max_ 3281, 3062, 2987, 2243, 1699, 1541, 1455, 1385, 1371, 1252, 1160, 1044, 1039 cm^−1^; ^1^H NMR (300 MHz, CDCl_3_) *δ* 6.03 (bs, 1H, N*H*), 4.19–4.09 (m, 4H, OC*H_2_*CH_3_), 3.36–3.18 (m, 2H, NHC*H_2_*CH_3_), 3.12 (d, ^2^*J*_PH_ = 13.8 Hz, 1H, C*H*-P), 1.79 (s, 3H, C*H*_3_)_,_ 1.34–1.28 (m, 6H, OCH_2_C*H*_3_) 1.13 (t, ^3^*J*_HH_ = 7.2 Hz, NHCH_2_C*H*_3_) ppm; ^13^C {^1^H} NMR (75 MHz, CDCl_3_) *δ* 158.8 (d, ^3^*J*_PC_ = 6.4 Hz, C=O), 116.9 (CN), 63.7 (d, ^2^*J*_PC_ = 5.7 Hz, OCH_2_), 63.0 (d, ^2^*J*_PC_ = 6.2 Hz, OCH_2_), 38.6 (d, ^1^*J*_PC_ = 207.0 Hz, CH-P), 36.2 (NH*C*H_2_CH_3_), 35.3 (d, ^2^*J*_PC_ = 3.2 Hz, C_quat_), 18.2 (CH_3_), 16.4 (d, ^3^*J*_PC_ = 5.2 Hz, OCH_2_*C*H_3_), 16.4 (d, ^3^*J*_PC_ = 5.3 Hz, OCH_2_*C*H_3_), 15.0 (NHCH_2_*C*H_3_) ppm; ^31^P NMR (120 MHz, CDCl_3_) *δ* 15.1 ppm; ESI-HRMS (CI) *m*/*z* calculated for C_11_H_21_N_3_O_4_P ([M + H]^+^) 290.1270 found 290.1275.


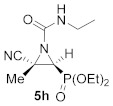


(*E*)-(2S*,3S*)-*N*-(*tert*-Butyl)-2-cyano-3-(diphenylphosphoryl)-2-methylaziridine-1-carboxamide (**5i**), (1.39 g, 73%) was obtained as a white solid from cyanoaziridine **1a** (1.41 g, 5 mmol) and *tert*-butyl isocyanate (1.14 mL, 10 mmol, 2 eq) as described in the general procedure (method C). The crude product was purified by crystallization from Et_2_O/pentane 50:50 to give the title compound **5i**; mp 166–168 °C; IR (neat) *v*_max_ 3259, 3056, 2976, 2237, 1707, 1541, 1452, 1441, 1369, 1285, 1208, 1127 cm^−1^; ^1^H NMR (400 MHz, CDCl_3_) *δ* 7.82–7.48 (m, 10H, Ar*H*), 5.53 (s, 1H, N*H*), 3.62 (d, ^2^*J*_PH_ = 22.1 Hz, 1H, C*H*-P), 1.85 (s, 3H, C*H_3_*), 1.33 (s, 9H, C(C*H*_3_)_3_) ppm; ^13^C {^1^H} NMR (75 MHz, CDCl_3_) *δ* 157.4 (d, ^3^*J*_PC_ = 4.6 Hz, C=O), 132.9, 132.9, 132.8, 132.8, 131.9, 131.6, 131.4, 131.3, 131.1, 131.0, 130.5, 130.2, 129.3, 129.2, 129.1, 129.0 (C_Ar_), 117.0 (CN), 52.1 (*C*(CH)_3_)_3_), 41.2 (d, ^1^*J*_PC_ = 99.9 Hz, CH-P), 36.5 (d, ^2^*J*_PC_ = 3.5 Hz, C_quat_), 28.7 (C(*C*H)_3_), 18.1 (CH_3_) ppm; ^31^P NMR (120 MHz, CDCl_3_) *δ* 23.1 ppm; ESI-HRMS (CI) *m*/*z* calculated for C_21_H_25_N_3_O_2_P ([M + H]^+^) 382.1684 found 382.1687.


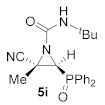


Diethyl (*E*)-[(2S*,3S*)-1-(*tert*-butylcarbamoyl)-3-cyano-3-methylaziridin-2-yl]phosphonate (**5j**), (1.19 g, 75%) was obtained as a white solid from cyanoaziridine **1c** (1.09 g, 5 mmol) and *tert*-butyl isocyanate (1.14 mL, 10 mmol, 2 eq) as described in the general procedure (method C). The crude product was purified by flash-column chromatography (SiO_2_, AcOEt/hexane 20:80) to give the title compound **5j**; mp 96–98 °C; IR (neat) *v*_max_ 3284, 3051, 2979, 2246, 1705, 1538, 1477, 1457, 1394, 1369, 1260, 1160, 1038 cm^−1^; ^1^H NMR (300 MHz, CDCl_3_) *δ* 5.45 (bs, 1H, N*H*), 4.21–4.11 (m, 4H, OC*H_2_*CH_3_), 3.13 (d, ^2^*J*_PH_ = 13.6 Hz, 1H, C*H*-P), 1.81 (s, 3H, C*H_3_*), 1.36–1.30 (m, 15H, C(C*H*_3_)_3_ + OCH_2_C*H*_3_) ppm; ^13^C {^1^H} NMR (75 MHz, CDCl_3_) *δ* 157.1 (d, ^3^*J*_PC_ = 6.7 Hz, C=O), 116.9 (d, ^3^*J*_PC_ = 2.4 Hz, CN), 63.7 (d, ^2^*J*_PC_ = 6.1 Hz, OCH_2_), 63.0 (d, ^2^*J*_PC_ = 6.5 Hz, OCH_2_), 52.1 (*C*(CH)_3_)_3_), 38.3 (d, ^1^*J*_PC_ = 207.1 Hz, CH-P), 35.3 (d, ^2^*J*_PC_ = 3.4 Hz, C_quat_), 28.7 (C(*C*H)_3_), 18.4 (CH_3_), 16.5 (d, ^3^*J*_PC_ = 5.9 Hz, OCH_2_*C*H_3_), 16.4 (d, ^3^*J*_PC_ = 5.6 Hz, OCH_2_*C*H_3_) ppm; ^31^P NMR (120 MHz, CDCl_3_) *δ* 15.5 ppm; ESI-HRMS (CI) *m*/*z* calculated for C_13_H_24_N_3_NaO_4_P ([M + Na]^+^) 340.1402 found 340.1400.


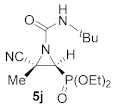


(*E*)-(2S*,3S*)-*N*-(*tert*-Butyl)-2-cyano-3-(diphenylphosphoryl)-2-ethylaziridine-1-carboxamide (**5k**), (1.27 g, 64%) was obtained as a white solid from cyanoaziridine **1b** (1.48 g, 5 mmol) and *tert*-butyl isocyanate (1.14 mL, 10 mmol, 2 eq) as described in the general procedure (method C). The crude product was purified by flash-column chromatography (SiO_2_, AcOEt/hexane 50:50) to give the title compound **5k**; mp 197–199 °C; IR (neat) *v*_max_ 3262, 2976, 2240, 1718, 1499, 1457, 1438, 1369, 1274, 1199, 1122 cm^−1^; ^1^H NMR (400 MHz, CDCl_3_) *δ* 7.83–7.45 (m, 10H, Ar*H*), 5.43 (s, 1H, N*H*), 3.67 (d, ^2^*J*_PH_ = 22.2 Hz, 1H, C*H*-P), 2.34–2.24 (m, 1H, C*H*_2_CH_3_), 2.01–1.99 (m, 1H, C*H*_2_CH_3_), 1.33 (s, 9H, C(C*H*_3_)_3_, 1.09 (t, ^3^*J*_HH_ = 1.9 Hz, 3H, CH_2_C*H*_3_) ppm; ^13^C {^1^H} NMR (100 MHz, CDCl_3_) *δ* 157.5 (C=O), 132.9, 132.8, 131.3, 131.2, 131.1, 131.0, 129.3, 129.2, 129.1, 129.0 (C_Ar_), 116.1 (CN), 52.0 (*C*(CH_3_)_3_), 42.3 (d, ^2^*J*_PC_ = 1.7 Hz, C_quat_), 41.9 (d, ^1^*J*_PC_ = 117.2 Hz, CH-P), 28.7 (C(*C*H)_3_), 24.6 (*C*H_2_CH_3_), 10.8 (CH_2_*C*H_3_) ppm; ^31^P NMR (120 MHz, CDCl_3_) *δ* 22.8 ppm; ESI-HRMS (CI) *m*/*z* calculated for C_22_H_27_N_3_O_2_P ([M + H]^+^) 396.1841 found 396.1847.


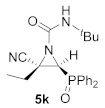


##### General Procedures and Spectral Data for The Addition of Ethoxycarbonyl Isothiocyanate to Functionalized Cyanoaziridines **1**

Method A. To a −30 °C solution of cyanoaziridine **1** (5 mmol, 1 eq) in CH_2_Cl_2_ (25 mL) ethoxycarbonyl isothiocyanate (6 mmol, 1.2 eq) was added dropwise. The reaction mixture was stirred at −30 °C for 6–8 h until TLC showed the disappearance of the starting cyanoaziridine. The crude products were concentrated to dryness in vacuum conditions and were purified by crystallization. Method B. To a 0 °C solution of cyanoaziridine **1** (5 mmol, 1 eq) in CH_2_Cl_2_ (25 mL) ethoxycarbonyl isothiocyanate (6 mmol, 1.2 eq) was added dropwise. The reaction mixture was allowed to reach room temperature and stirred for 6–24 h. The crude product was concentrated to dryness in vacuum conditions and was purified by crystallization.

Ethyl (*E*)-[(2S*,3S*)-2-cyano-3-(diphenylphosphoryl)-2-methylaziridine-1-carbonothioyl]carbamate (**6a**), (1.47 g, 71%) was obtained as an orange solid from cyanoaziridine **1a** (1.41 g, 5 mmol) and ethoxycarbonyl isothiocyanate (0.71 mL, 6 mmol, 1.2 eq) as described in the general procedure (method A). The crude product was purified by crystallization from Et_2_O to give the title compound **6a**. (1.66 g, 80%) which was obtained as an orange solid from cyanoaziridine **1a** (1.41 g, 5 mmol) and ethoxycarbonyl isothiocyanate (0.71 mL, 6 mmol, 1.2 eq) as described in the general procedure (method B). The crude product was purified by crystallization from Et_2_O to give the title compound **6a**; mp 156–158 °C; IR (neat) v_max_ 3406, 3147, 2984, 2254, 1771, 1593, 1491, 1438, 1383, 1233, 1152, 1122, 1041 cm^−1^; ^1^H NMR (400 MHz, CDCl_3_) δ 9.43 (bs, 1H, N*H*), 7.94–7.42 (m, 10H, Ar*H*), 4.17 (q, ^3^*J*_HH_ = 7.1 Hz, 2H, OC*H_2_*), 3.92 (d, ^2^*J*_PH_ = 20.0 Hz, 1H, C*H*-P), 1.97 (s, 3H, C*H_3_*). A value of 1.23 (t, ^3^*J*_HH_ = 7.1 Hz, 3H, C*H_3_*) ppm; ^13^C {^1^H} NMR (75 MHz, CDCl_3_) δ 190.5 (d, ^3^*J*_PC_ = 4.6 Hz, C=S), 149.0 (C=O), 133.0, 133.0, 132.8, 132.8, 131.8, 131.7, 131.5, 131.2, 131.1, 130.9, 130.1, 129.4, 129.3, 129.1, 128.8, 128.6 (C_Ar_), 116.5 (CN), 63.0 (CH_2_), 48.8 (d, ^1^*J*_PC_ = 94.5 Hz, CH-P), 42.4 (d, ^2^*J*_PC_ = 3.2 Hz, C_quat_), 18.6 (CH_3_), 14.2 (CH_3_) ppm; ^31^P NMR (120 MHz, CDCl_3_) δ 22.7 ppm; ESI-HRMS (CI) *m*/*z* calculated for C_20_H_21_N_3_O_3_PS ([M + H]^+^) 414.1041, found 414.1041.


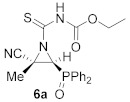


Ethyl (*E*)-[(2S*,3S*)-2-cyano-3-(diphenylphosphoryl)-2-ethylaziridine-1-carbonothioyl]carbamate (**6b**), (1.82 g, 85%) was obtained as a pale yellow solid from cyanoaziridine **1b** (1.48 g, 5 mmol) and ethoxycarbonyl isothiocyanate (0.71 mL, 6 mmol, 1.2 eq) as described in the general procedure (method A). The crude product was purified by crystallization from Et_2_O to give the title compound **6b**; mp 179–181 °C; IR (neat) v_max_ 3409, 3062, 2981, 2254, 2237, 1752, 1541, 1438, 1230, 1197, 1163, 1044 cm^−1^; ^1^H NMR (400 MHz, CDCl_3_) δ 8.96 (bs, 1H, N*H*), 7.97–7.44 (m, 10H, Ar*H*), 4.20 (q, ^3^*J*_HH_ = 7.2 Hz, 2H, OC*H_2_*), 3.96 (d, ^2^*J*_PH_ = 20.2 Hz, 1H, C*H*-P), 2.59–2.50 (m, 1H, C*H_2_*), 2.37–2.28 (m, 1H, C*H_2_*). A value of 1.27 (t, ^3^*J*_HH_ = 7.2 Hz, 3H, C*H_3_*), 0.99 (t, ^3^*J*_HH_ = 7.5 Hz, C*H_3_*) ppm; ^13^C {^1^H} NMR (75 MHz, CDCl_3_) δ 190.1 (d, ^3^*J*_PC_ = 4.4 Hz, C=S), 149.0 (C=O), 133.1, 133.0, 132.8, 132.8, 131.8, 131.6, 131.5, 131.4, 131.3, 131.2, 130.1, 129.9, 129.3, 129.2, 128.9, 128.7 (C_Ar_), 115.4 (CN), 63.2 (OCH_2_), 48.8 (d, ^1^*J*_PC_ = 94.4 Hz, CH-P), 48.0 (d, ^2^*J*_PC_ = 3.1 Hz, C_quat_), 24.6 (CH_2_), 14.3 (CH_3_), 10.4 (CH_3_) ppm; ^31^P NMR (120 MHz, CDCl_3_) δ 21.8 ppm; ESI-HRMS (CI) *m*/*z* calculated for C_21_H_23_N_3_O_3_PS ([M + H]^+^) 428.1198, found 428.1204.


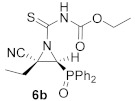


Ethyl (*E*)-[(2S*,3S*)-2-cyano-3-(diethoxyphosphoryl)-2-methylaziridine-1-carbonothioyl]carbamate (**6c**), (1.50 g, 86%) was obtained as an orange solid from cyanoaziridine **1c** (1.09 g, 5 mmol) and ethoxycarbonyl isothiocyanate (0.71 mL, 6 mmol, 1.2 eq) as described in the general procedure (method B). The crude product was purified by crystallization from Et_2_O/pentane to give the title compound **6c**; mp 116–118 °C; IR (neat) v_max_ 3395, 3162, 2987, 2251, 1774, 1491, 1385, 1241, 1158, 1041 cm^−1^; ^1^H NMR (300 MHz, CDCl_3_) δ 9.03 (bs, 1H, N*H*), 4.30–4.14 (m, 6H, OC*H_2_*CH_3_ + C*H_2_*CH_3_), 3.41 (d, ^2^*J*_PH_ = 12.3 Hz, 1H, C*H*-P), 1.98 (s, 3H, C*H_3_*), 1.38–1.27 (m, 9H, OCH_2_C*H_3_* + CH_2_C*H_3_*) ppm; ^13^C {^1^H} NMR (75 MHz, CDCl_3_) δ 190.3 (d, ^3^*J*_PC_ = 6.7 Hz, C=S), 148.7 (C=O), 116.4 (d, ^3^*J*_PC_ = 2.3 Hz, CN), 64.2 (d, ^2^*J*_PC_ = 5.8 Hz, O*C*H_2_CH_3_), 63.3 (d, ^2^*J*_PC_ = 6.9 Hz, O*C*H_2_CH_3_), 63.2 (CH_2_), 45.8 (d, ^1^*J*_PC_ = 201.7 Hz, CH-P), 41.8 (d, ^2^*J*_PC_ = 3.2 Hz, C_quat_), 18.6 (CH_3_), 16.5 (d, ^3^*J*_PC_ = 4.0 Hz, OCH_2_*C*H_3_), 16.4 (d, ^3^*J*_PC_ = 6.0 Hz, OCH_2_*C*H_3_), 14.2 (CH_2_*C*H_3_) ppm; ^31^P NMR (120 MHz, CDCl_3_) δ 13.3 ppm; ESI-HRMS (CI) *m*/*z* calculated for C_12_H_21_N_3_O_5_PS ([M + H]^+^) 350.0940 found 350.0932.


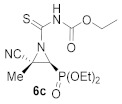


##### General Procedure and Spectral Data for The Reaction of NaI with *N*-Carbamoyl Cyanoaziridines **7**

To a stirred solution of *N*-functionalized cyanoaziridine **5** (5 mmol, 1 eq) in THF (15 mL), NaI (0.02 g, 1 mmol, 0.2 eq) was added dropwise. The mixture was heated at 60 °C for 24 h until TLC showed the disappearance of the starting cyanoaziridine. NaI was filtered through a sintered glass vacuum filtration funnel with celite and washed with THF. The filtrate was concentrated to dryness in vacuum conditions and the resulting residue was purified by flash-column chromatography.

(4S*,5S*)-5-(Diphenylphosphoryl)-4-methyl-2-(phenylamino)-4,5-dihydrooxazole-4-carbonitrile (**7a**) and (4S*,5S*)-4-(diphenylphosphoryl)-5-methyl-2-(phenylamino)-4,5-dihydrooxazole-5-carbonitrile (**7′a**), (0.90 g, 45%) were obtained as yellow solids from *N*-functionalized cyanoaziridine **5a** (2.00 g, 5 mmol) as described in the general procedure. The crude product was purified by flash-column chromatography (SiO_2_, AcOEt/hexane 50:50) to give the minor regioisomer; mp 117–119 °C; IR (neat) *v*_max_ 3420, 3057, 2981, 2237, 1674, 1438, 1402, 1199, 1122 cm^−1^; ^1^H NMR (400 MHz, CDCl_3_) *δ* 7.95–7.20 (m, 15H, Ar*H*), 4.23 (bs, 1H, N*H*), 3.00 (d, ^2^*J*_PH_ = 16.2 Hz, 1H, C*H*-P), 1.76 (s, 3H, C*H_3_*) ppm; ^13^C {^1^H} NMR (75 MHz, CDCl_3_) *δ* 164.6 (d, ^3^*J*_PC_ = 5.9 Hz, C=N), 132.9, 132.1, 131.9, 131.2, 131.1, 130.4, 130.0, 129.4, 129.2, 129.1, 128.9, 126.8 (C_Ar_), 127.6 (CN), 51.8 (d, ^1^*J*_PC_ = 95.5 Hz, CH-P), 29.8 (C_quat_), 12.2 (CH_3_) ppm; ^31^P NMR (120 MHz, CDCl_3_) *δ* 21.0 ppm; ESI-HRMS (CI) *m*/*z* calculated for C_23_H_21_N_3_O_2_P ([M + H]^+^) 402.1371 found 402.1368.


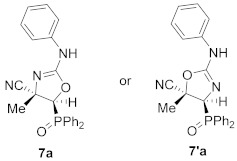


Diethyl (*E*)-[(4S*,5S*)-4-cyano-4-methyl-2-(phenylamino)-4,5-dihydroozazol-5-yl]phosphonate (**7b**) and diethyl (*E*)-[(4S*,5S*)-5-cyano-5-methyl-2-(phenylamino)-4,5-dihydroozazol-4-yl]phosphonate (**7′b**), (1.16 g, 69%) were obtained as waxy white solids from *N*-functionalized cyanoaziridine **5b** (1.68 g, 5 mmol) as described in the general procedure. The crude product was purified by flash-column chromatography (SiO_2_, AcOEt) to give the title compound **7** as a mixture of two regioisomers **7b** + **7′b**; Rf: 0.1 (AcOEt); IR (neat) *v*_max_ 3370, 3061, 2990, 2240, 1666, 1499, 1402, 1255, 1158, 1052, 1019 cm^−1^; ^1^H NMR (300 MHz, CDCl_3_) *δ* 8.10 (bs, 1H, N*H*), 7.52–7.12 (m, 11H, Ar*H* + N*H*), 4.32–4.20 (m, 8H, OC*H*_2_), 2.65 (d, ^2^*J*_PH_ = 11.0 Hz, 1H, C*H*-P)_major_, 2.62 (d, ^2^*J*_PH_ = 11.1 Hz, 1H, C*H*-P)_minor_, 1.89 (s, 3H, C*H_3_*)_major_, 1.84 (s, 3H, C*H_3_*)_minor_, 1.40–1.33 (m, 12H, OCH_2_C*H*_3_) ppm; ^13^C {^1^H} NMR (75 MHz, CDCl_3_) *δ* 166.5 (d, ^3^*J*_PC_ = 3.7 Hz, C=N)_minor_, 161.8 (d, ^3^*J*_PC_ = 4.5 Hz, C=N)_major_, 132.4 (C_quat_), 130.9 (C_quat_), 130.3, 129.7, 129.3, 128.7, 127.7, 127.0, 126.7 (C_Ar_), 121.6 (CN)_minor_, 121.5 (CN)_major_, 64.3 (d, ^2^*J*_PC_ = 6.0 Hz, OCH_2_)_minor_, 64.0 (d, ^2^*J*_PC_ = 6.0 Hz, OCH_2_)_major_, 63.3 (d, ^2^*J*_PC_ = 6.2 Hz, OCH_2_)_major_, 48.4 (d, ^1^*J*_PC_ = 201.8 Hz, CH-P)_major_, 47.8 (d, ^1^*J*_PC_ = 201.3 Hz, CH-P)_minor_, 47.5 (d, ^2^*J*_PC_ = 3.4 Hz, C_quat_)_major_, 46.0 (d, ^2^*J*_PC_ = 3.00 Hz, C_quat_)_minor_, 16.6, 16.5, 16.5, 16.4 (OCH_2_*C*H_3_), 12.5 (CH_3_)_major_, 12.1 (CH_3_)_minor_ ppm; ^31^P NMR (120 MHz, CDCl_3_) *δ* 13.8_major_, 13.3_minor_ ppm; ESI-HRMS (CI) *m*/*z* calculated for C_15_H_21_N_3_O_4_P ([M + H]^+^) 338.1270 found 338.1254.


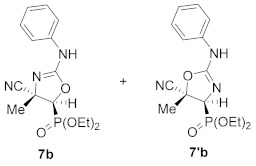


(*E*)-(4S*,5S*)-5-(Diphenylphosphoryl)-4-methyl-2-(*p*-tolylamino)-4,5-dihydrooxazole-4-carbonitrile (**7c**) and (*E*)-(4S*,5S*)-4-(diphenylphosphoryl)-5-methyl-2-(*p*-tolylamino)-4,5-dihydrooxazole-5-carbonitrile (**7′c**), (1.56 g, 75%) were obtained as white solids from *N*-functionalized cyanoaziridine **5c** (2.07 g, 5 mmol) as described in the general procedure. The crude product was purified by flash-column chromatography (AcOEt) to give the title compound **7** as a mixture of two regioisomers **7c** +**7′c**; mp 128–130 °C; IR (neat) *v*_max_ 3425, 3059, 2959, 2235, 1617, 1516, 1438, 1405, 1197, 1119 cm^−1^; ^1^H NMR (400 MHz, CDCl_3_) *δ* 8.01 (bs, 1H, N*H*), 7.96–7.01 (m, 29H, Ar*H* + N*H*), 2.98 (d, ^2^*J*_PH_ = 15.5 Hz, 1H, C*H*-P)_minor_, 2.98 (d, ^2^*J*_PH_ = 16.7 Hz, 1H, C*H*-P)_major_, 2.37 (s, 3H, C*H_3_*)_major_, 2.33 (s, 3H, C*H_3_*)_minor_, 1.80 (s, 3H, C*H_3_*)_major_, 1.68 (s, 3H, C*H_3_*)_minor_ ppm; ^13^C {^1^H} NMR (75 MHz, CDCl_3_) *δ* 166.8 (C=N)_minor_, 162.1 (C=N)_major_, 140.0 (C_quat_), 138.8 (C_quat_), 132.9, 132.0, 131.9, 131.3, 131.2, 131.1, 131.0, 130.4, 130.0, 129.7, 129.3, 129.2, 129.0, 128.9, 128.2, 127.5, 126.8, 126.4 (C_Ar_), 121.5 (d, ^3^*J*_PC_ = 3.8 Hz, CN), 51.5 (d, ^1^*J*_PC_ = 95.9 Hz, CH-P)_major_, 51.6 (d, ^1^*J*_PC_ = 95.2 Hz, CH-P)_minor_, 48.8 (d, ^2^*J*_PC_ = 3.1Hz C_quat_)_major_, 47.1 (C_quat_), 21.3 (CH_3_), 12.3 (CH_3_)_major_, 11.9 (CH_3_)_minor_ ppm; ^31^P NMR (120 MHz, CDCl_3_) *δ* 21.0_major_, 20.8_minor_ ppm; ESI-HRMS (CI) *m*/*z* calculated for C_24_H_23_N_3_O_2_P ([M + H]^+^) 416.1528 found 416.1544.


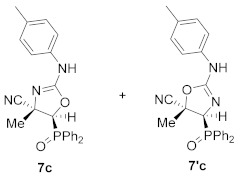


Diethyl (*E*)-[(4S*,5S*)-4-cyano-4-methyl-2-(*p*-tolylamino)-4,5-dihydroozazol-5-yl]phosphonate (**7d**) and diethyl (*E*)-[(4S*,5S*)-5-cyano-5-methyl-2-(*p*-tolylamino)-4,5-dihydroozazol-5-yl]phosphonate (**7′d**), (0.95 g, 54%) were obtained as waxy white solids from *N*-functionalized cyanoaziridine **5d** (1.75 g, 5 mmol) as described in the general procedure. The crude product was purified by flash-column chromatography (SiO_2_, AcOEt) to give the title compound **7d** + **7′d** as a mixture of two regioisomers; Rf: 0.1 (AcOEt); IR (neat) *v*_max_ 3356, 3037, 2987, 2237, 1671, 1516, 1444, 1402, 1321, 1260, 1160, 1127, 1049, 1024 cm^−1^; ^1^H NMR (300 MHz, CDCl_3_) *δ* 8.05 (bs, 1H, N*H*)_minor_, 7.49 (bs, 1H, N*H*)_major_, 7.31–7.00 (m, 8H, Ar*H*), 4.33–4.21 (m, 8H, OC*H*_2_), 2.62 (d, ^2^*J*_PH_ = 11.1 Hz, 1H, C*H*-P)_major_, 2.60 (d, ^2^*J*_PH_ = 11.3 Hz, 1H, C*H*-P)_minor_, 2.37 (s, 3H, C*H_3_*)_major_, 2.33 (s, 3H, C*H_3_*)_minor_, 1.89 (s, 3H, C*H_3_*)_major_, 1.85 (s, 3H, C*H_3_*)_minor_, 1.41–1.33 (m, 12H, OCH_2_C*H*_3_) ppm; ^13^C {^1^H} NMR (75 MHz, CDCl_3_) *δ* 166.7 (d, ^3^*J*_PC_ = 3.0 Hz, C=N)_minor_, 162.1 (d, ^1^*J*_PC_ = 3.7 Hz, C=N)_major_, 140.0 (C_quat_), 138.9 (C_quat_), 131.0, 130.6, 130.1, 129.7, 128.2, 126.9, 126.5 (C_Ar_), 127.6 (CN)_major_, 121.3 (d, ^3^*J*_PC_ = 5.5 Hz, CN)_minor_, 64.3 (d, ^2^*J*_PC_ = 6.0 Hz, OCH_2_)_minor_, 64.0 (d, ^2^*J*_PC_ = 5.9 Hz, OCH_2_)_minor_, 63.3 (d, ^2^*J*_PC_ = 6.2 Hz, OCH_2_)_major_, 48.4 (d, ^1^*J*_PC_ = 201.8 Hz, CH-P)_major_, 47.9 (d, ^1^*J*_PC_ = 201.8 Hz, CH-P)_minor_, 47.5 (d, ^2^*J*_PC_ = 3.2 Hz, C_quat_)_major_, 46.0 (C_quat_)_minor_, 21.3 (CH_3_), 16.6, 16.6, 16.5, 16.5 (OCH_2_*C*H_3_), 12.5 (CH_3_)_major_, 12.2 (CH_3_)_minor_ ppm; ^31^P NMR (120 MHz, CDCl_3_) *δ* 13.9_major_, 13.4_minor_ ppm; ESI-HRMS (CI) *m*/*z* calculated for C_16_H_23_N_3_O_4_P ([M + H]^+^) 352.1426 found 352.1426.


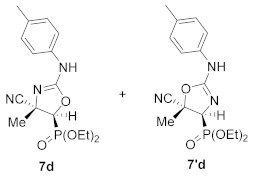


##### General Procedure and Spectral Data for Compound **8a**

To a −70 °C solution of **6a** (5 mmol, 1 eq) in THF (25 mL) boron trifluoride diethyl etherate (25 mmol, 5 eq) was added dropwise. The reaction mixture was stirred at −70 °C for 24 h until TLC showed the disappearance of the starting *N*-functionalized cyanoaziridine. The crude product was washed three times with water (15 mL) and extracted with CH_2_Cl_2_ (15 mL). The organic layers were dried over anhydrous MgSO_4_, filtered, and concentrated to dryness in vacuum conditions, and the resulting residue was purified by flash-column chromatography.

Ethyl (*Z*)-[(4R*,5S*)-5-cyano-4-(diphenylphosphoryl)-5-methyl-4,5-dihydrothiazol-2-yl]carbamate (**8a**), (1.39 g, 67%) was obtained as a pale yellow solid from *N*-functionalized cyanoaziridine **6a** (2.06 g, 5 mmol) and boron trifluoride diethyl etherate (3.1 mL, 25 mmol, 5 eq) as described in the general procedure. The crude product was purified by flash-column chromatography (SiO_2_, AcOEt/hexane 50:50) to give the title compound **8a**; mp 208–210 °C; IR (neat) *v*_max_ 3145, 3065, 2937, 2254, 2232, 1724, 1624, 1507, 1438, 1244, 1174, 1113, 1094 cm^−1^; ^1^H NMR (400 MHz, CDCl_3_) *δ* 8.06–7.40 (m, 11H, Ar*H* + N*H*), 4.58 (d, ^2^*J*_PH_ = 12.8 Hz, 1H, C*H*-P), 4.22–4.16 (m, 2H, C*H*_2_), 2.11 (s, 3H, C*H_3_*), 1.25 (t, ^3^*J*_HH_ = 7.1 Hz, 3H, CH_2_C*H*_3_) ppm; ^13^C {^1^H} NMR (75 MHz, CDCl_3_) *δ* 157.1 (d, ^3^*J*_PC_ = 19.4 Hz, C=N), 152.8 (C=O), 134.0 (C_quat_), 133.3, 133.2, 132.7, 132.6, 132.5, 132.4, 132.4, 131.3, 131.2, 129.2, 128.9, 128.7, 128.2, 128.1, 127.9 (C_Ar_), 118.8 (d, ^3^*J*_PC_ = 7.1 Hz, CN), 76.6 (d, ^1^*J*_PC_ = 82.7 Hz, CH-P), 63.1 (CH_2_), 52.0 (d, ^2^*J*_PC_ = 1.3 Hz, C_quat_), 26.4 (CH_3_), 14.4 (CH_3_) ppm; ^31^P NMR (120 MHz, CDCl_3_) *δ* 26.1 ppm; ESI-HRMS (CI) *m*/*z* calculated for C_20_H_21_N_3_O_3_PS ([M + H]^+^) 414.1041 found 414.1046.


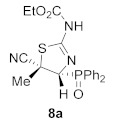


##### General Procedure and Spectral Data for The Reaction of Cyanoaziridines **1** and Isocyanates in The Ppresence of KI

A mixture of the corresponding isocyanate (2 mmol, 2 eq), KI (0.25 g, 0.3 mmol) and cyanoaziridine (1 mmol, 1 eq) in CH_3_CN (15 mL) was stirred at 60 °C until TLC showed the disappearance of the starting cyanoaziridine. After the completion of the reaction, the solvent was evaporated under reduced pressure and the crude product was washed three times with water (15 mL) and extracted with CH_2_Cl_2_ (15 mL). The organic layers were dried over anhydrous MgSO_4_, filtered and concentrated to dryness in vacuum conditions, and the resulting residue was purified by crystallization or by flash-column chromatography.

(*E*)-(4S*,5S*)-4-Cyano-5-(diphenylphosphoryl)-4-ethyl-*N*-phenyl-2-(phenylimino)oxazolidine-3-carboxamide (**9a**), (1.66 g, 62%) was obtained as a white solid from cyanoaziridine **5a** (1.48 g, 5 mmol) and phenyl isocyanate (1.09 mL, 10 mmol, 2 eq) as described in the general procedure. The crude product was purified by flash-column chromatography (SiO_2_, AcOEt/hexane 40:60) to give the title compound **9a**; mp 208–210 °C; IR (neat) *v*_max_ 3267, 3062, 2973, 2246, 1779, 1560, 1502, 1435, 1383, 1316, 1260, 1225, 1119 cm^−1^; ^1^H NMR (300 MHz, CDCl_3_) *δ* 8.01–7.07 (m, 21H, Ar*H* + N*H*), 3.32 (d, ^2^*J*_PH_ = 18.7 Hz, 1H, C*H*-P), 2.46–2.27 (m, 2H, C*H*_2_), 1.05 (t, ^3^*J*_HH_ = 7.4 Hz, 3H, CH_2_C*H_3_*) ppm; ^13^C {^1^H} NMR (75 MHz, CDCl_3_) *δ* 164.6 (d, ^3^*J*_PC_ = 6.0 Hz, C=N), 157.9 (C=O), 138.0 (C_quat_), 133.1, 133.0, 132.2, 132.1, 131.9, 131.8, 131.7, 129.0, 126.9, 124.2, 119.2 (C_Ar_), 121.6 (CN), 55.1 (d, ^1^*J*_PC_ = 96.5 Hz, CH-P), 54.5 (C_quat_), 18.9 (CH_2_), 10.3 (CH_3_) ppm; ^31^P NMR (120 MHz, CDCl_3_) *δ* 24.1 ppm; ESI-HRMS (CI) *m*/*z* calculated for C_31_H_28_N_4_O_3_P ([M + H]^+^) 535.1899 found 535.1899.


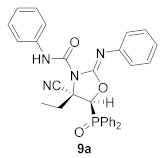


(*E*)-(4S*,5S*)-4-Cyano-5-(diphenylphosphoryl)-4-methyl-*N*-(*p*-tolyl)-2-(*p*-tolylimino)oxazolidine-3-carboxamide (**9b**), (1.51 g, 55%) was obtained as a white solid from cyanoaziridine **5c** (1.41 g, 5 mmol) and *p*-tolyl isocyanate (1.26 mL, 10 mmol, 2 eq) as described in the general procedure. The crude product was purified by flash-column chromatography (SiO_2_, AcOEt/hexane 50:50) to give the title compound **9b**; mp 220–222 °C; IR (neat) *v*_max_ 3259, 3040, 2926, 2246, 1777, 1613, 1596, 1513, 1438, 1391, 1241, 1172, 1191, 1155 cm^−1^; ^1^H NMR (300 MHz, CDCl_3_) *δ* 8.03–7.01(m, 19H, Ar*H* + N*H*), 3.21 (d, ^2^*J*_PH_ = 18.2 Hz, 1H, C*H*-P), 2.33 (s, 3H, C*H*_3_), 2.29 (s, 3H, C*H*_3_), 1.91 (s, 3H, C*H_3_*) ppm; ^13^C {^1^H} NMR (75 MHz, CDCl_3_) *δ* 164.6 (d, ^3^*J*_PC_ = 6.0 Hz, C=N), 158.1 (C=O), 139.4 (C_quat_), 135.4 (C_quat_), 133.8 (C_quat_), 133.1, 132.9, 132.9, 132.0, 131.9, 131.8, 130.0, 129.7, 129.5, 129.3, 129.2, 128.9, 126.7, 120.9, 120.3, 119.2, (C_Ar_ + CN), 53.9 (d, ^1^*J*_PC_ = 92.0 Hz, CH-P), 49.1 (d, ^2^*J*_PC_ = 3.8 Hz, C_quat_), 21.3 (CH_3_), 21.0 (CH_3_), 12.6 (CH_3_) ppm; ^31^P NMR (120 MHz, CDCl_3_) *δ* 23.7 ppm; ESI-HRMS (CI) *m*/*z* calculated for C_32_H_30_N_4_O_3_P ([M + H]^+^) 549.2056 found 549.2056.


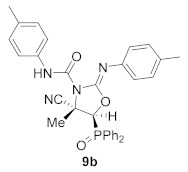


Diethyl (*E*)-[(4S*,5S*)-4-cyano-4-methyl-3-(phenylcarbamoyl)-2-(phenylimino)oxazolidin-5-yl]phosphonate (**9c**), (1.80 g, 79%) was obtained as a pale yellow solid from cyanoaziridine **1c** (1.09 g, 5 mmol) and phenyl isocyanate (1.09 mL, 10 mmol, 2 eq) as described in the general procedure. The crude product was purified by crystallization from Et_2_O to give the title compound **9c**; mp 169–171 °C; IR (neat) *v*_max_ 3231, 3140, 2985, 2249, 1716, 1610, 1580, 1499, 1488, 1313, 1249, 1194, 1052, 1024 cm^−1^; ^1^H NMR (300 MHz, CDCl_3_) *δ* 7.47–7.02 (m, 10H, Ar*H*), 6.58 (d, 1H, N*H*), 4.19–4.02 (m, 4H, OC*H*_2_), 3.98 (d, ^2^*J*_PH_ = 12.4 Hz, 1H, C*H*-P), 1.98 (s, 3H, C*H*_3_), 1.24–1.16 (m, 3H, OCH_2_C*H*_3_) ppm; ^13^C {^1^H} NMR (75 MHz, CDCl_3_) *δ* 177.3 (d, ^2^*J*_PC_ = 15.9 Hz, C=N), 158.5 (C=O) 151.2 (d, ^3^*J*_PC_ = 12.4 Hz, C_quat_), 145.2 (C_quat_), 134.5 (C_quat_), 129.5, 129.3, 129.1, 128.6, 128.5, 127.9, 127.7, 124.4, 123.7, 123.5 (C_Ar_), 120.1 (CN), 80.3 (d, ^2^*J*_PC_ = 6.1 Hz, C_quat_), 64.4 (d, ^2^*J*_PC_ = 6.8 Hz, OCH_2_), 63.7 (d, ^2^*J*_PC_ = 6.7 Hz, OCH_2_), 52.0 (d, ^1^*J*_PC_ = 156.6 Hz, CH-P), 19.2 (CH_3_), 16.3 (d, ^2^*J*_PC_ = 5.8 Hz, OCH_2_*C*H_3_) ppm; ^31^P NMR (120 MHz, CDCl_3_) *δ* 15.1 ppm; ESI-HRMS (CI) *m*/*z* calculated for C_22_H_26_N_4_O_5_P ([M + H]^+^) 457.1641 found 457.1629.


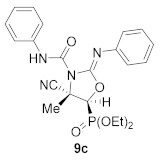


### 3.2. Biology

#### 3.2.1. Materials

Reagents and solvents were used as purchased without further purification. All stock solutions of the investigated compounds were prepared by dissolving the powered materials in appropriate amounts of DMSO. The final concentration of DMSO never exceeded 10% (*v*/*v*) in reactions. The stock solution was stored at 5 °C until it was used.

#### 3.2.2. Cytotoxicity Assays

Cells were cultured according to the supplier’s instructions. Cells were seeded in 96-well plates at a density of 2–4 × 10^3^ cells per well and incubated overnight in 0.1 mL of media supplied with 10% Fetal Bovine Serum (Lonza) in a 5% CO_2_ incubator at 37 °C. On day 2, the compounds were added, and the samples were incubated for 48 h. After treatment, 10 μL of the cell counting kit-8 was added into each well for an additional 2 h incubation at 37 °C. The absorbance of each well was determined by an Automatic Elisa Reader System at a 450 nm wavelength.

## 4. Conclusions

In summary, we herein report the activation of *N*H-cyanoaziridines with phosphorus substituents by *N*-acylation or *N*-carbamoylation reactions. As far as we know, this methodology constitutes the first example of *N*-functionalization of phosphorus-substituted *N*H-cyanoaziridines with iso(thio)cyanates for the preparation of *N*-(thio)carbamoyl cyanoaziridines derived from phosphine oxide and phosphonate. Furthermore, we examined the ring expansion reaction of synthesized cyanoaziridines for the preparation of 5-membered nitrogen-containing heterocycles. For instance, *N*-acylated cyanoaziridine **2a** regioselectively isomerized (Heine-type reaction) to 4-cyanooxazoline **3a** in the presence of NaI. However, when *N*-carbamoyl cyanoaziridines **5** react in the same reaction conditions, 2-aminocyanooxazolines **7** are achieved as a mixture of regioisomers. The Heine-type reaction of *N*-thiocarbamoyl cyanoaziridine **6a** was performed using mild acidic conditions (BF_3_·OEt_2_ as a Lewis acid), since neither thermal nor nucleophilic conditions produced the corresponding 2-aminocyanothiazoline **8a**. We also examined the one pot reaction of cyanoaziridines **1** with isocyanates. The ring expansion reaction of *N*-carbamoyl cyanoaziridines **5** in situ prepared by the reaction of cyanoaziridines **1** with isocyanates, followed by the insertion of a second equivalent of isocyanate, obtained 2-iminocyanooxazolidines **9** in a regioselective way. Additionally, we evaluated the cytotoxic effect of all the synthesized compounds inhibiting the growth of the human tumor cell lines A549 (carcinomic human alveolar basal epithelial cells). Within the *N*-acylated and *N*-(thio)carbamoylated cyanoaziridines, only compound **2a** exhibited a moderate cytotoxic effect with an IC_50_ of 22.9 ± 1.9μM. Concerning the 5-membered nitrogen-containing heterocycles, 4-cyanooxazoline **3a** showed a IC_50_ value of 19.7 ± 2.8 μM, since 2-iminooxazolidines **9** exhibited IC_50_ values between 6.2 ± 0.7 and 16.4 ± 1.5 μM. In addition, the cytotoxic effect of our compounds in healthy lung cells, fibroblast lung cells (MRC-5), seemed not to present any effect.

## Figures and Tables

**Table 1:** Phosphorus substituted *N*-aryl or *N*-alkylcarbamoyl cyanoaziridines **5** obtained.

** 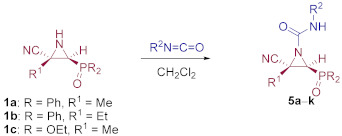 **					
**Entry**	**Compound**	**R**	**R^1^**	**R^2^**	**Yield (%) ^a^**
1	**5a**	Ph	Me	Ph	63
2	**5a**	Ph	Me	Ph	71 ^b^
3	**5b**	OEt	Me	Ph	98
4	**5c**	Ph	Me	*p*-MeC_6_H_4_	82
5	**5d**	OEt	Me	*p*-MeC_6_H_4_	80
6	**5e**	Ph	Et	*p*-MeC_6_H_4_SO_2_	86
7	**5f**	OEt	Me	*p*-MeC_6_H_4_SO_2_	79
8	**5g**	Ph	Me	Et	86 ^c^
9	**5h**	OEt	Me	Et	59 ^c^
10	**5i**	Ph	Me	*^t^*Bu	73 ^c^
11	**5j**	OEt	Me	*^t^*Bu	75 ^c^
11	**5k**	Ph	Et	*^t^*Bu	64 ^c^

^a^ Isolated yield of purified compounds **5**. ^b^ Reaction conditions: isocyanate (3 eq) and Sc (OTf)_3_ (20% mol) in CH_2_Cl_2_ at 0 °C. ^c^ Reaction conditions: isocyanate (2 eq) and ZnCl_2_ (1.25 eq) in CH_2_Cl_2_ at 25 °C.

**Table 2:** Phosphorus substituted *N*-ethoxycarbonylthiocarbamoyl cyanoaziridines **6** obtained.

** 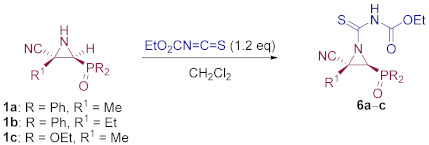 **					
**Entry**	**Compound**	**R**	**R^1^**	**Method ^a^**	**Yield (%) ^b^**
1	**6a**	Ph	Me	A	71
2	**6a**	Ph	Me	B	80
3	**6b**	Ph	Et	A	85
4	**6c**	OEt	Me	B	86

^a^ Reaction conditions. Method A: isothiocyanate (1.2 eq) in CH_2_Cl_2_ at −30 °C. Method B: isothiocyanate (1.2 eq) in CH_2_Cl_2_ at 25 °C. ^b^ Isolated yield of purified compounds **6**.

**Table 3:** Regioisomeric phosphorus substituted oxazolines derivatives **7** obtained.

** 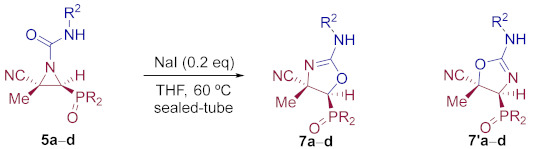 **					
**Entry**	**Compound**	**R**	**R^2^**	**Yield (%) ^a^**	**Ratio ^b^**
1	**7a** + **7′a**	Ph	Ph	45	66:34
2	**7b** + **7′b**	OEt	Ph	69	64:36
3	**7c** + **7′c**	Ph	*p*-MeC_6_H_4_	75	65:35
4	**7d** + **7′d**	OEt	*p*-MeC_6_H_4_	54	66:34

^a^ Isolated yield of purified oxazolines **7**. ^b^ Regioisomeric ratio was determined by crude ^31^P NMR spectra.

**Table 4:** Preparation of iminoxazolidines **9**.

Entry	Compound	R	R^1^	R^2^	Yield (%) ^a^
1	**9a**	Ph	Et	Ph	62
2	**9b**	Ph	Me	*p*-MeC_6_H_4_	55
3	**9d**	OEt	Me	Ph	79

^a^ Isolated yield of purified compounds **9**.

**Table 5:** Antiproliferative activity of synthesized *N*-functionalized cyanoaziridines **2**, **5,** and **6** and 5-membered nitrogen-containing heterocycles **3a**, **7**, **8a** and **9**.

Entry	Comp.	R	R^1^	R^2^	Cytotoxicity IC_50_ (μM) ^a^	
					Lung A549	MRC-5
1	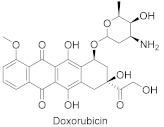				0.48 ± 0.017 [50]	>50 [51]
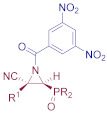						
2	**2a**	Ph	Me	–	22.9 ± 1.9	>50
3	**2b**	Ph	Et	–	>50	>50
4	**2c**	OEt	Me	–	>50	>50
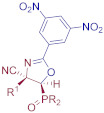						
5	**3a**	Ph	Me	–	19.7± 2.8	>50
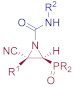						
6	**5a**	Ph	Me	Ph	>50	>50
7	**5b**	OEt	Me	Ph	>50	>50
8	**5c**	Ph	Me	*p*-MeC_6_H_4_	>50	>50
9	**5d**	OEt	Me	*p*-MeC_6_H_4_	>50	>50
10	**5e**	Ph	Et	*p*-MeC_6_H_4_SO_2_	>50	>50
11	**5f**	OEt	Me	*p*-MeC_6_H_4_SO_2_	>50	>50
12	**5g**	Ph	Me	Et	>50	>50
13	**5h**	OEt	Me	Et	>50	>50
14	**5i**	Ph	Me	*^t^*Bu	>50	>50
15	**5j**	OEt	Me	*^t^*Bu	>50	>50
16	**5k**	Ph	Et	*^t^*Bu	>50	>50
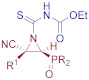						
17	**6a**	Ph	Me	–	>50	>50
18	**6b**	Ph	Et	–	>50	>50
19	**6c**	OEt	Me	–	>50	>50
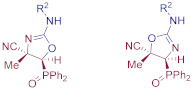						
20	**7a** + **7′a**	Ph	Me	Ph	>50	>50
21	**7b** + **7′b**	OEt	Me	Ph	>50	>50
22	**7c** + **7′c**	Ph	Me	*p*-MeC_6_H_4_	>50	>50
23	**7d** + **7′d**	OEt	Me	*p*-MeC_6_H_4_	>50	>50
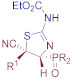						
24	**8a**	Ph	Me	–	>50	>50
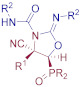						
25	**9a**	Ph	Et	Ph	16.4 ± 1.5	>50
26	**9b**	Ph	Me	*p*-MeC_6_H_4_	14.8 ± 1.2	>50
27	**9c**	OEt	Me	Ph	6.2 ± 0.7	>50

^a^ The cytotoxicity IC_50_ values listed are the concentrations corresponding to 50% growth inhibition.

## Data Availability

The data presented in this study are available in the Supplementary Materials.

## Supplementary Materials

The following are available online: ^1^H and ^13^C NMR spectra of synthesized compounds **2**, **3**, **5**–**9**.

## Sample Availability

Samples of the compounds are not available from the authors.
