# Supplementary material for: Synthesis and Antiproliferative Activity of Phosphorus Substituted 4-Cyanooxazolines, 2-Aminocyanooxazolines, 2-Iminocyanooxazolidines and 2-Aminocyanothiazolines by Rearrangement of Cyanoaziridines
Source: Molecules. 2021 Jul 14;26(14):4265. doi: 10.3390/molecules26144265 (PMC8305992; doi:10.3390/molecules26144265)

Supplementary Information

Synthesis and antiproliferative activity of phosphorus substituted 4-cyanooxazolines, 2-aminocyanooxazolines, 2-iminocyanooxazolidines, and 2-aminocyanothiazolines by rearrangement of cyanoaziridines

Victor Carramiñana, Ana M. Ochoa de Retana, Francisco Palacios*, Jesús M. de los Santos*

Department of Organic Chemistry I, Faculty of Pharmacy and Lascaray Research Center, University of the Basque Country (UPV/EHU), Paseo de la Universidad 7, 01006 Vitoria, Spain.
* Correspondence: francisco.palacios@ehu.eus (F.P.); jesus.delossantos@ehu.eus; (J.M.S.)

Table of contents

^{1}H and ^{13}C NMR spectra of *N*-acylaziridines **2** Page S-2

^{1}H and ^{13}C NMR spectra of 4-cyanooxazoline **3a** Page S-5

^{1}H and ^{13}C NMR spectra of *N*-functionalized cyanoaziridines **5a–k** Page S-6

^{1}H and ^{13}C NMR spectra of *N*-thiocarbamoyl cyanoaziridines **6a–c** Page S-17

^{1}H and ^{13}C NMR spectra of oxazolines**7a–c** Page S-20

^{1}H and ^{13}C NMR spectra of 2-aminothiazoline **8a** Page S-24

^{1}H and ^{13}C NMR spectra of oxazolidines **9a–c** Page S-25

^{1}H NMR (300 MHz, $CDCl_3$) of compound **2a**.

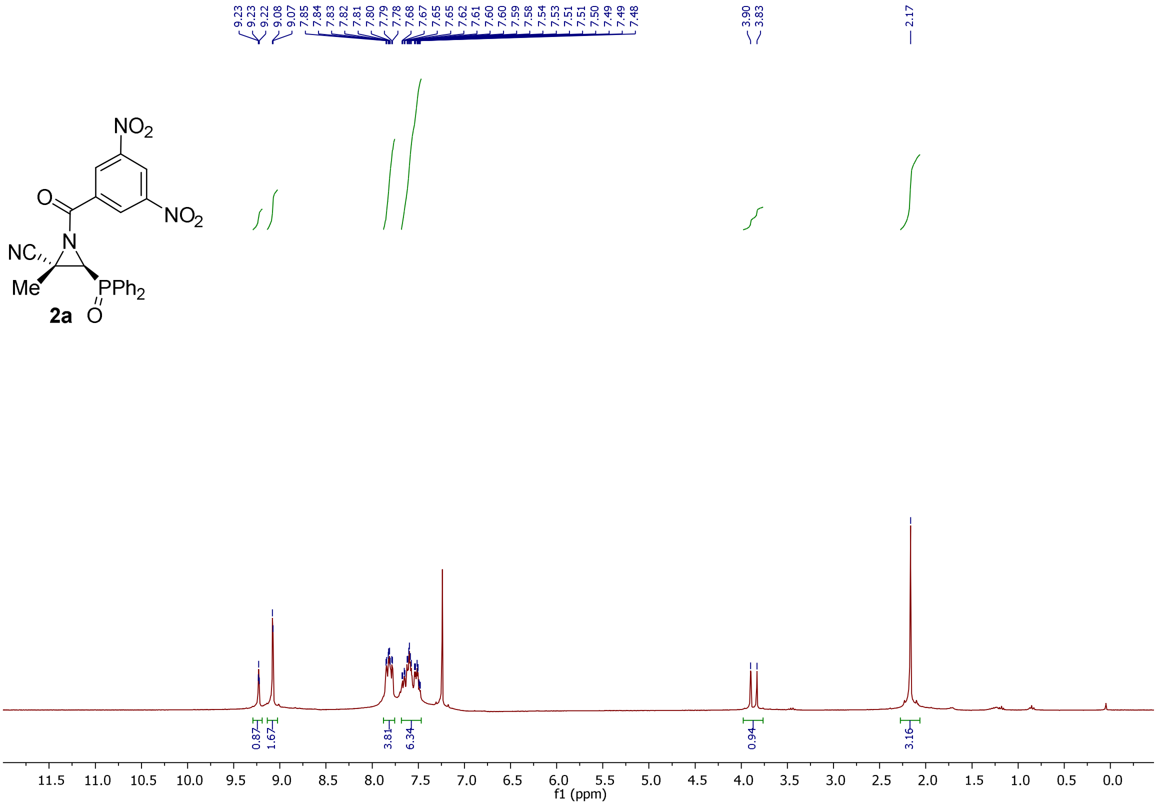

^{13}C {^{1}H} NMR (75 MHz, $CDCl_3$) of compound **2a**.

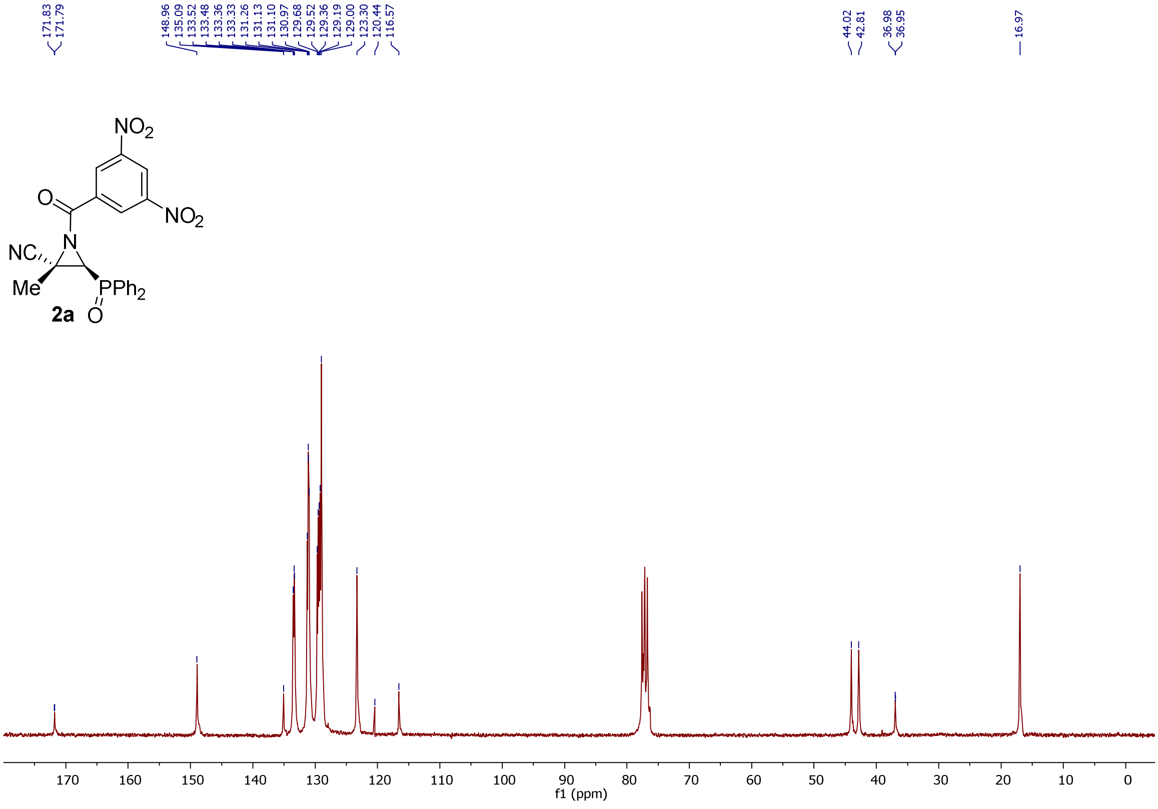

^{1}H NMR (300 MHz, $CDCl_3$) of compound **2b**.

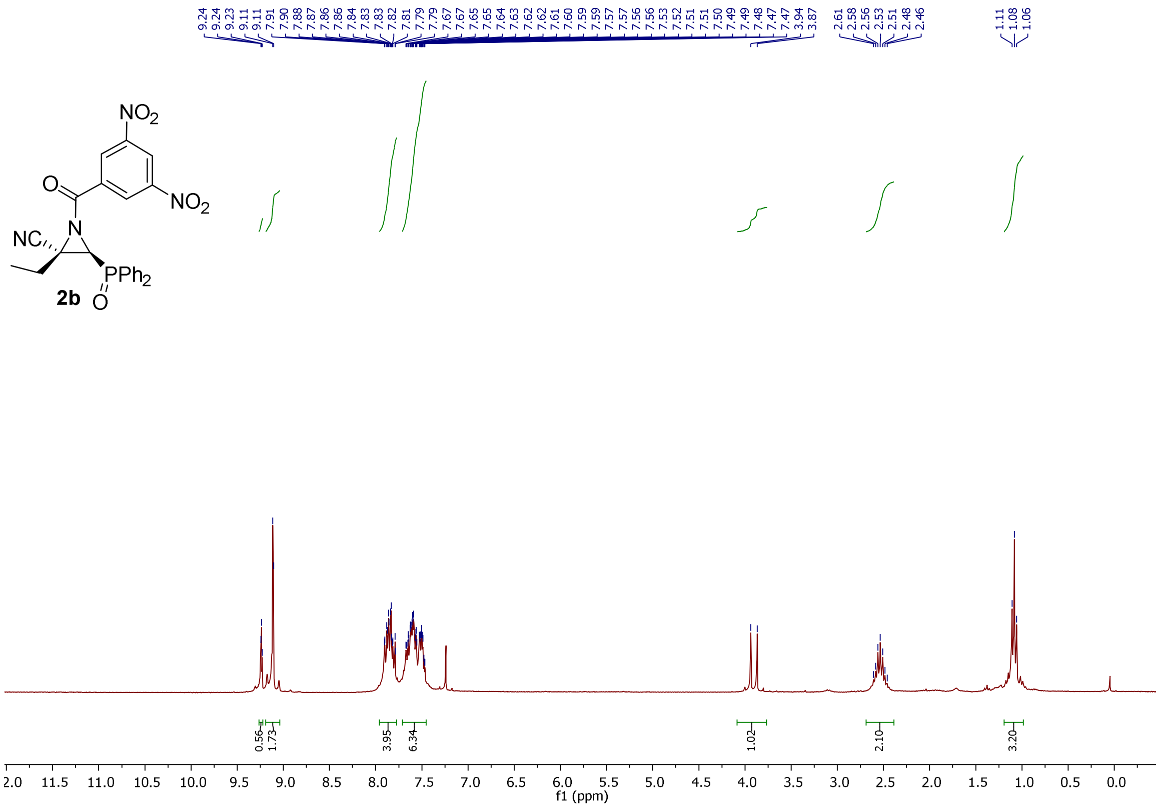

^{13}C {^{1}H} NMR (75 MHz, $CDCl_3$) of compound **2b**.

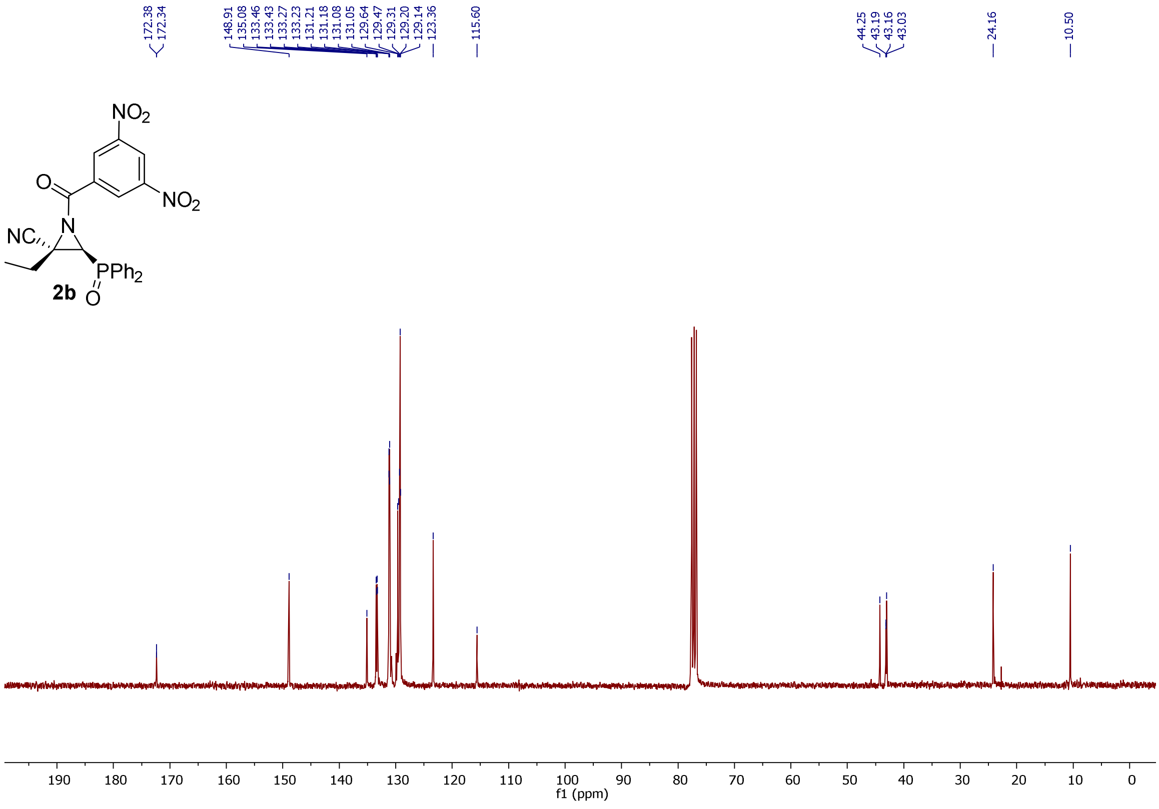

^{1}H NMR (300 MHz, $CDCl_3$) of compound **2c**.

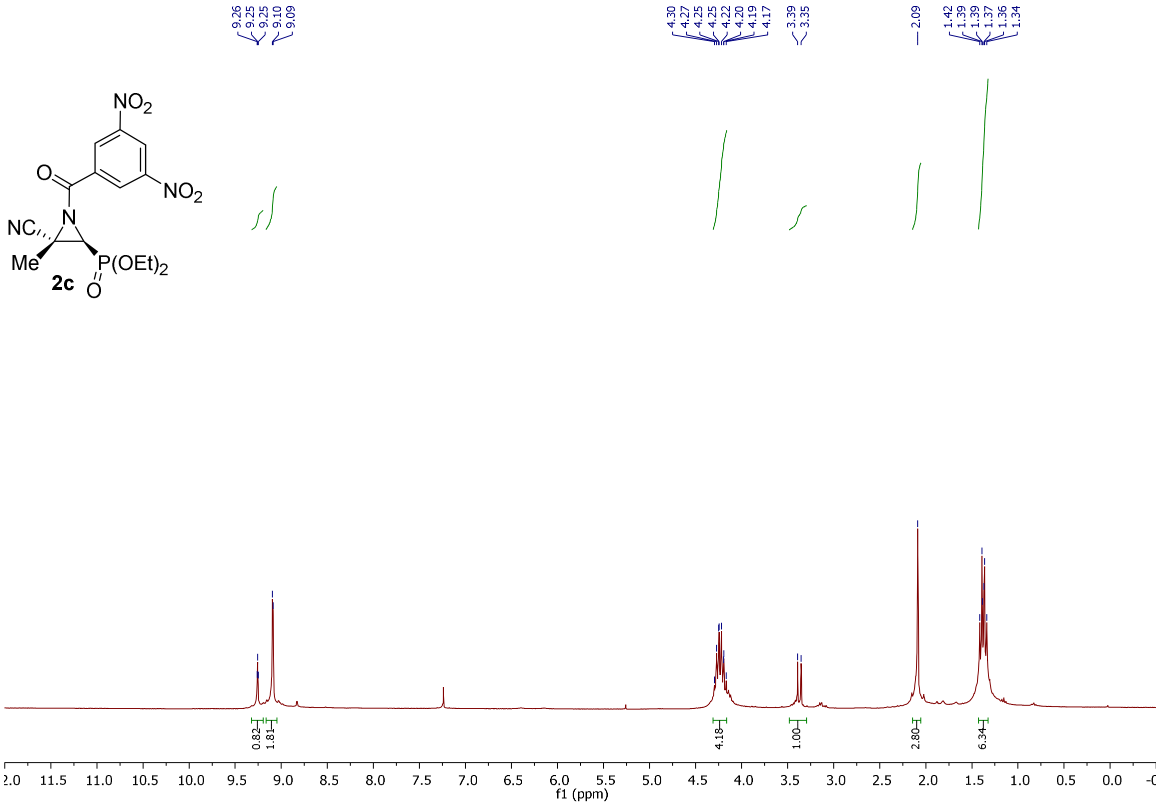

^{13}C {^{1}H} NMR (75 MHz, $CDCl_3$) of compound **2c**.

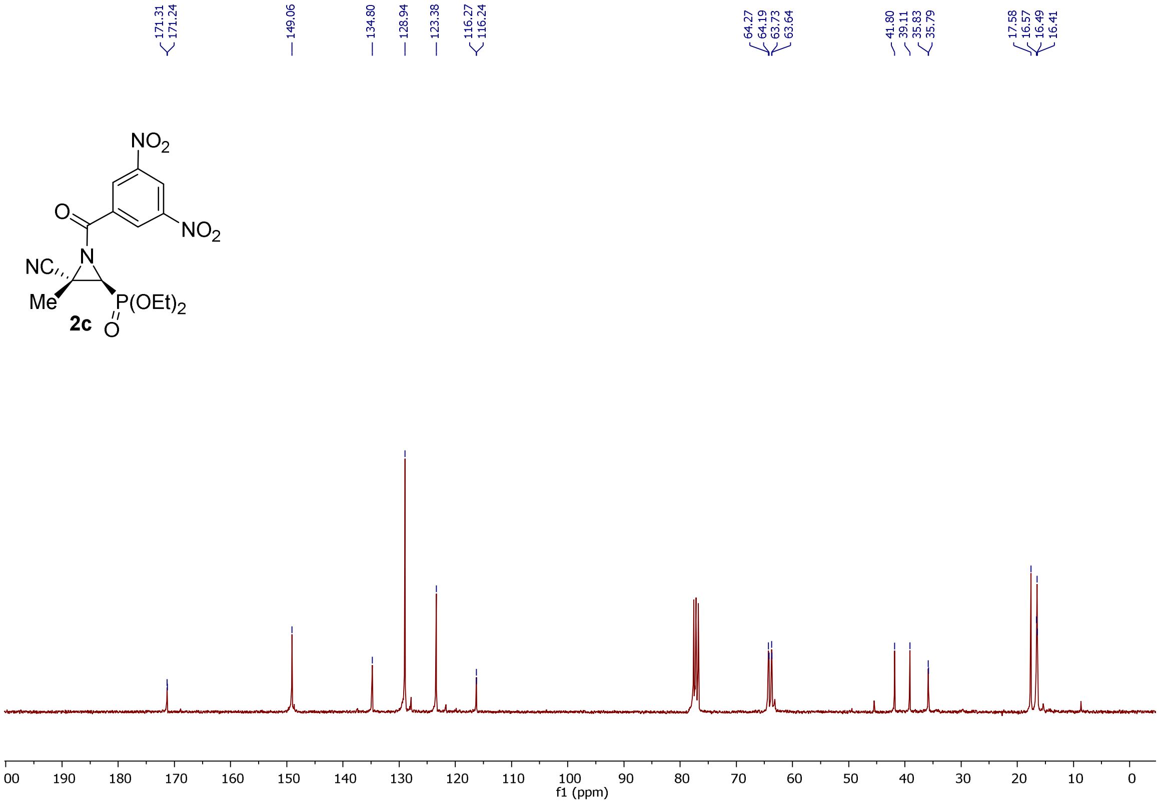

^{1}H NMR (300 MHz, $CDCl_3$) of compound **3a**.

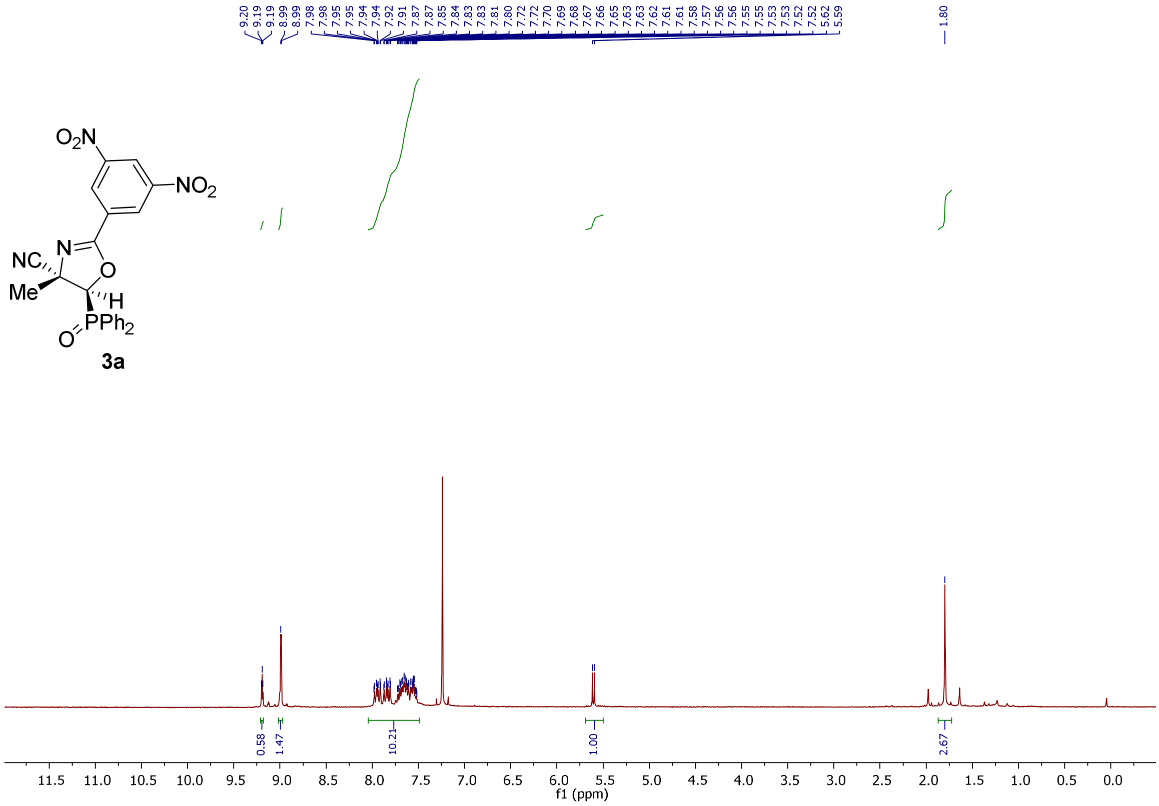

^{13}C {^{1}H} NMR (75 MHz, $CDCl_3$) of compound **3a**.

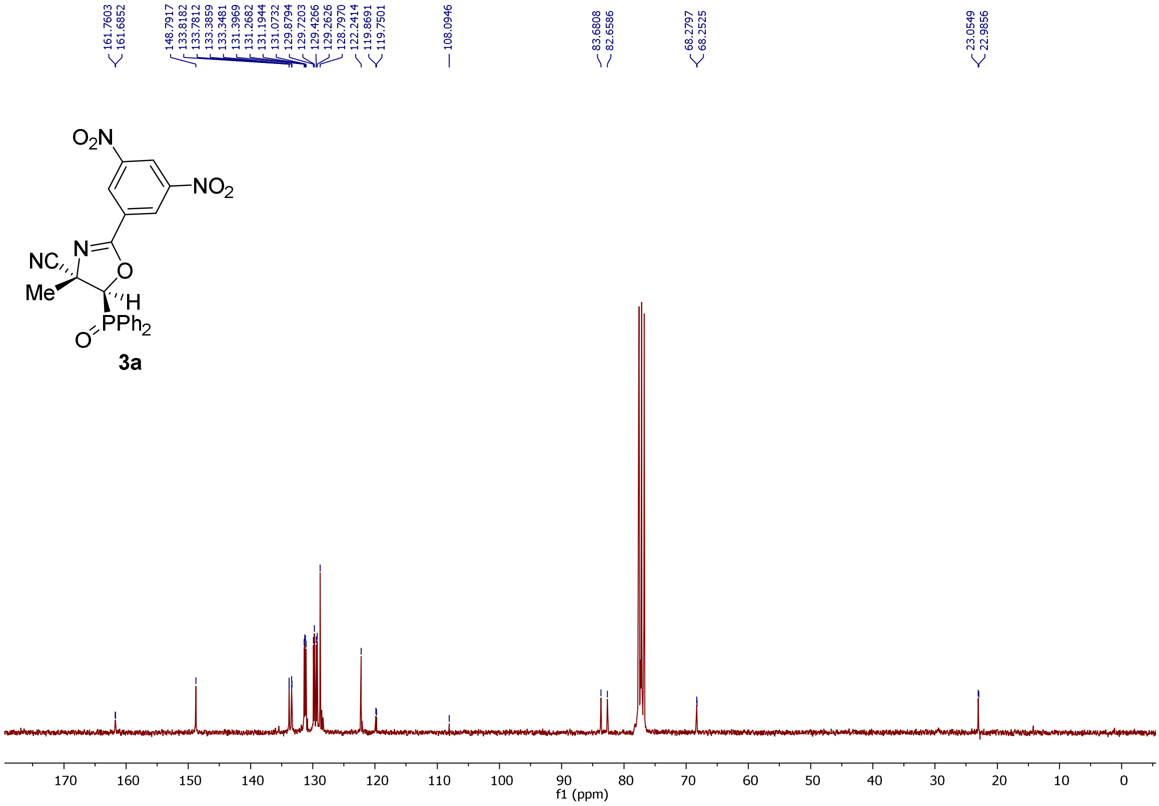

^{1}H NMR (400 MHz, $CDCl_3$) of compound **5a**.

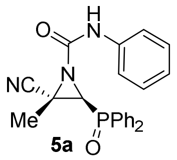

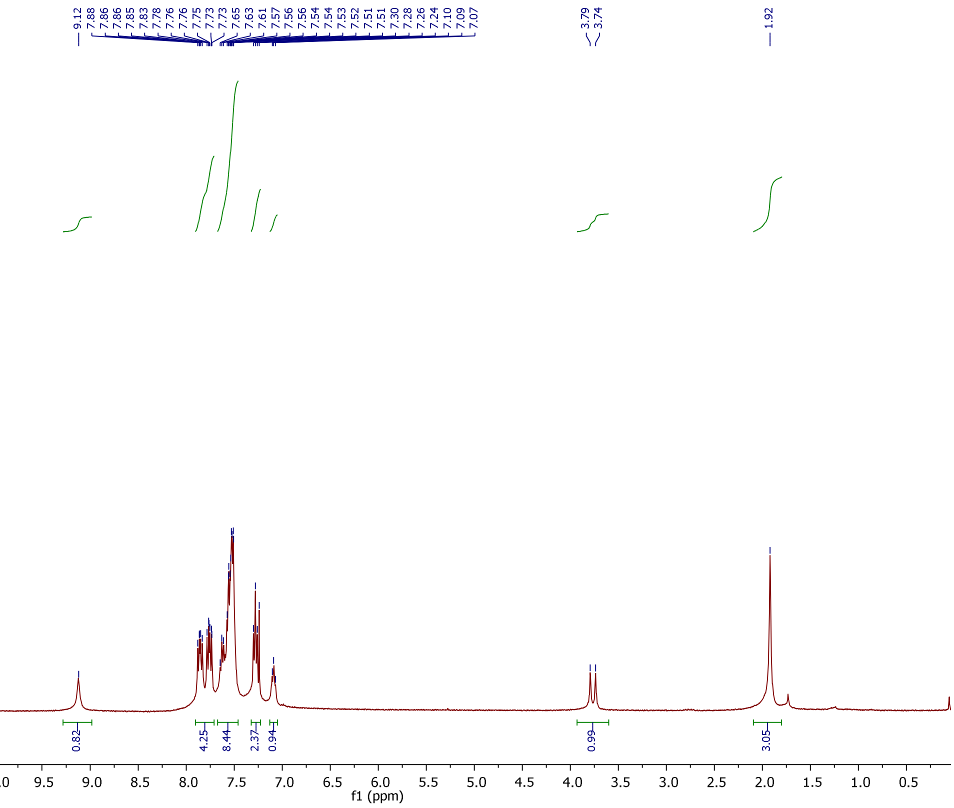

^{13}C NMR (75 MHz, $CDCl_3$) of compound **5a**.

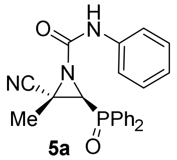

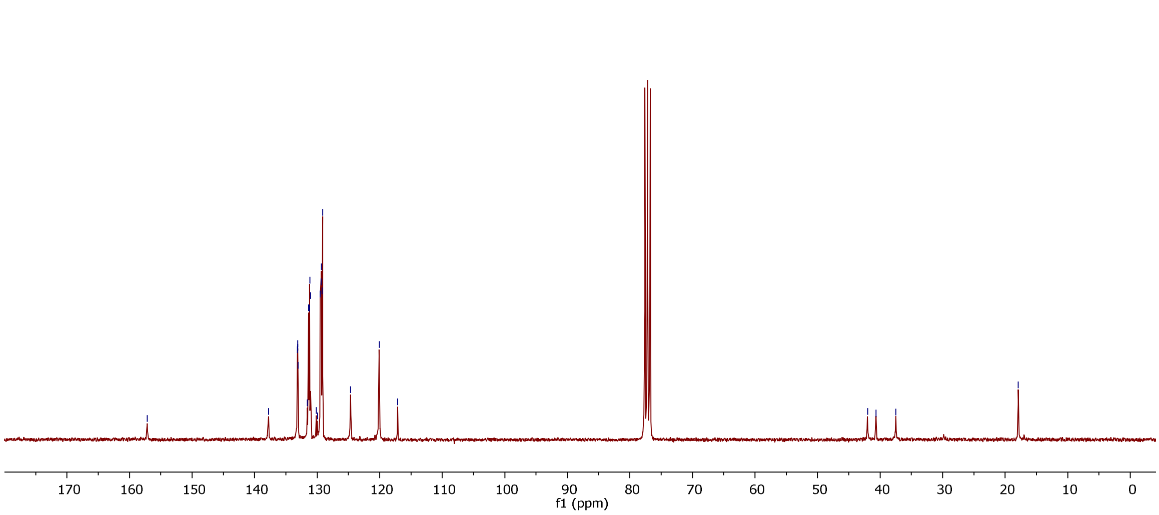

^1H NMR (300 MHz, $CDCl_3$) of compound **5b**.

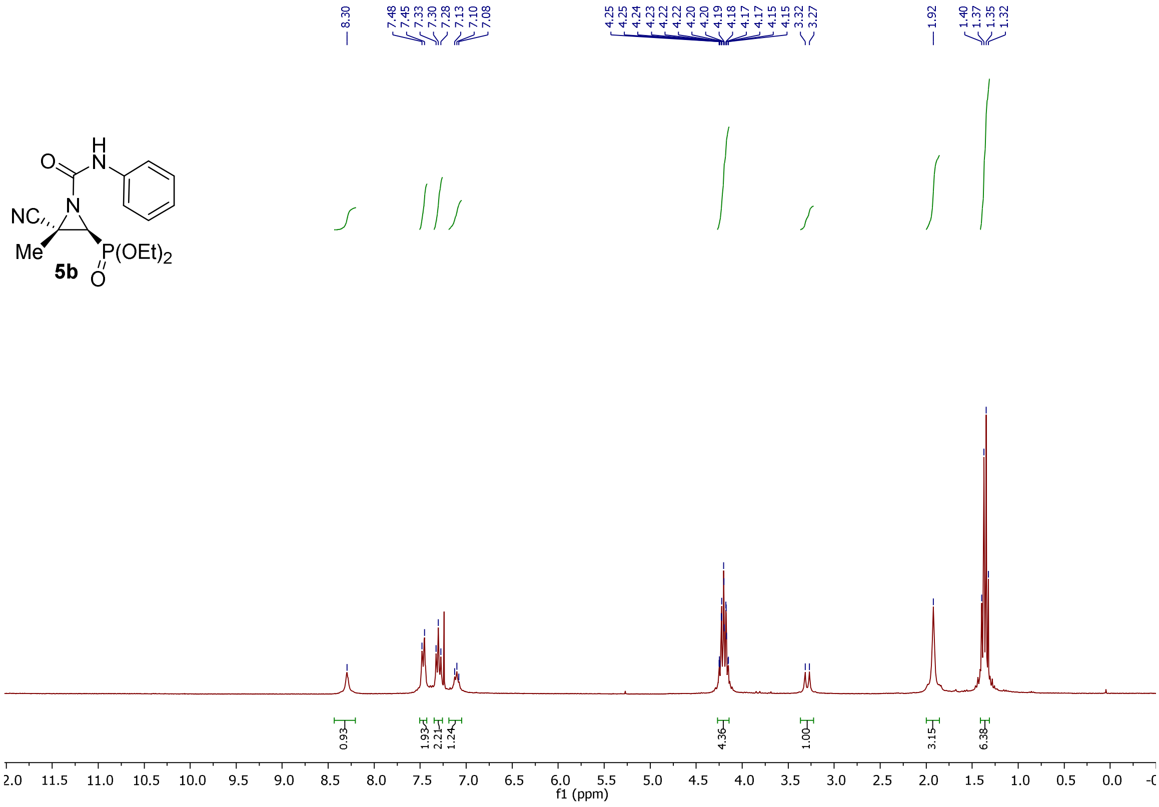

^{13}C {^1H} NMR (75 MHz, $CDCl_3$) of compound **5b**.

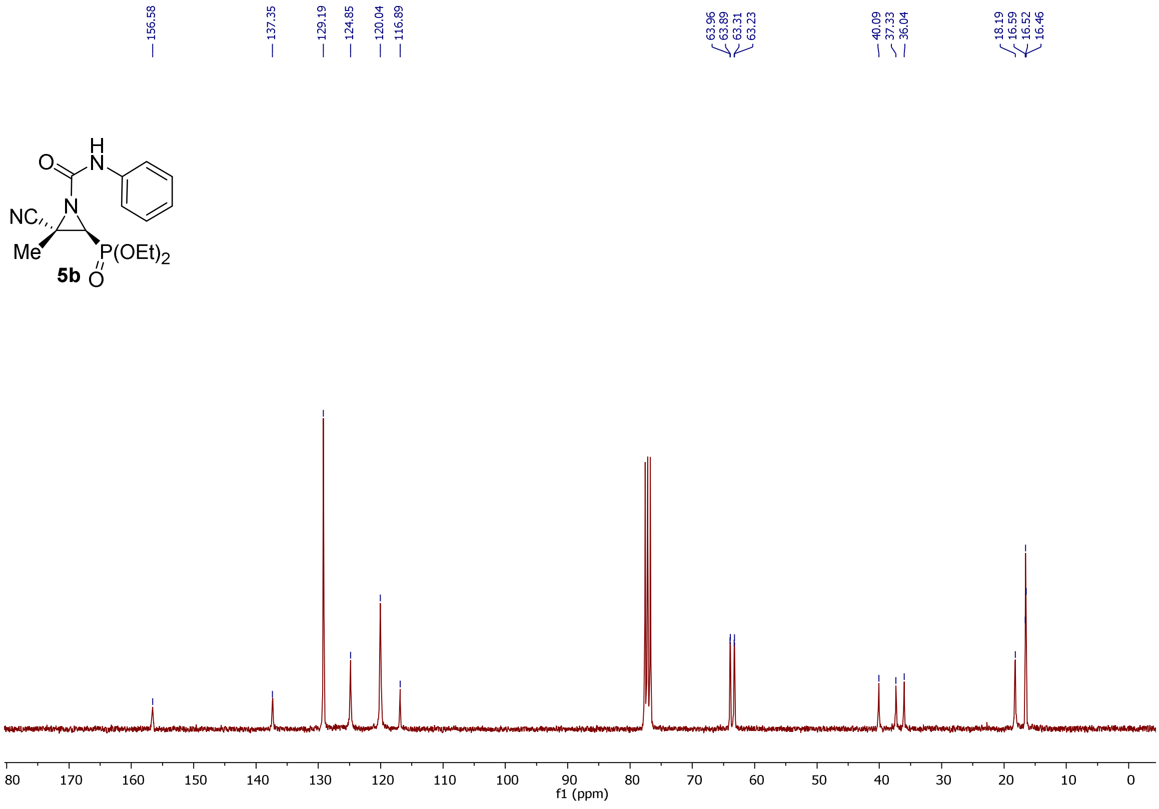

^1H NMR (400 MHz, $CDCl_3$) of compound **5c**.

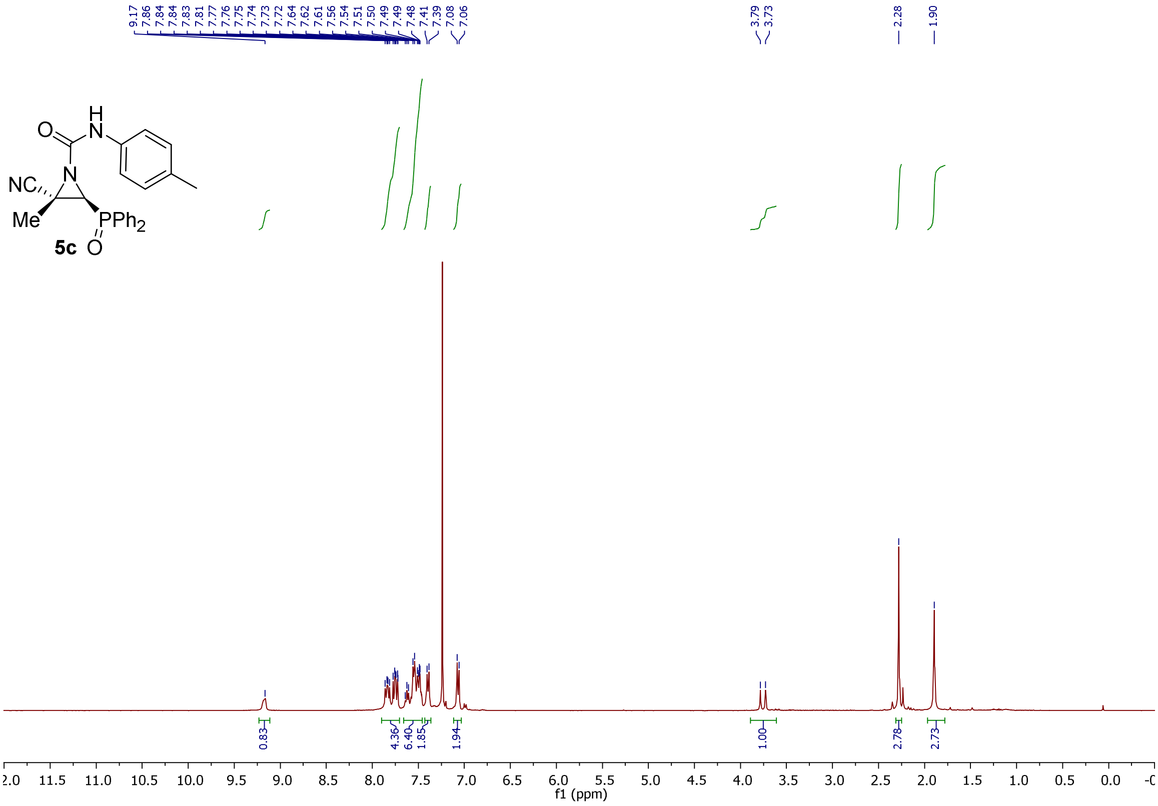

^{13}C {^1H} NMR (75 MHz, $CDCl_3$) of compound **5c**.

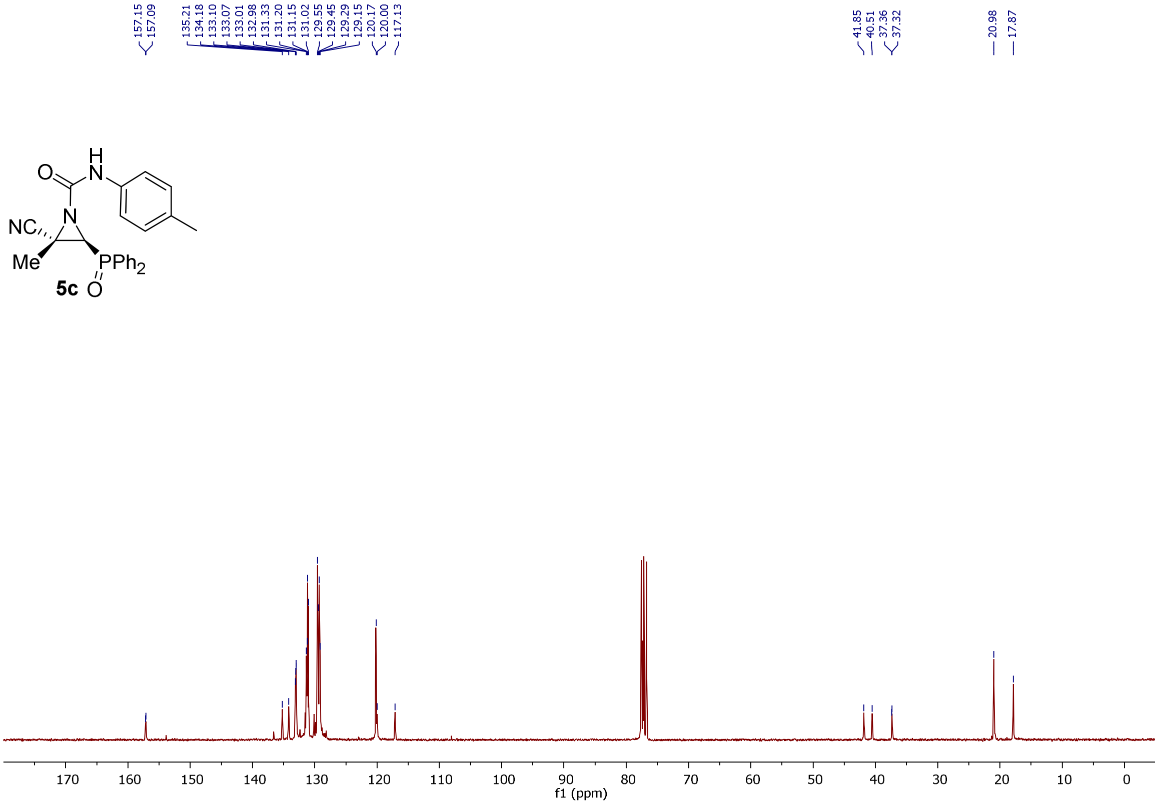

^{1}H NMR (300 MHz, $CDCl_3$) of compound **5d**.

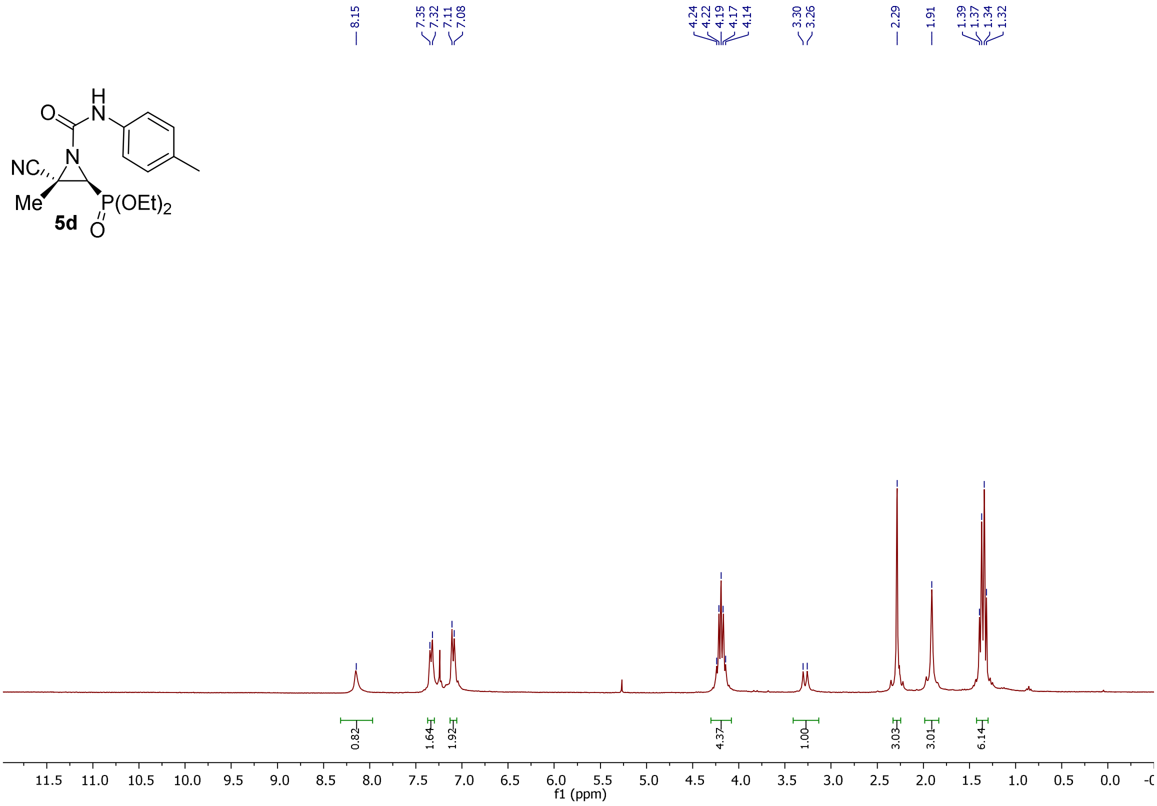

^{13}C {^{1}H} NMR (75 MHz, $CDCl_3$) of compound **5d**.

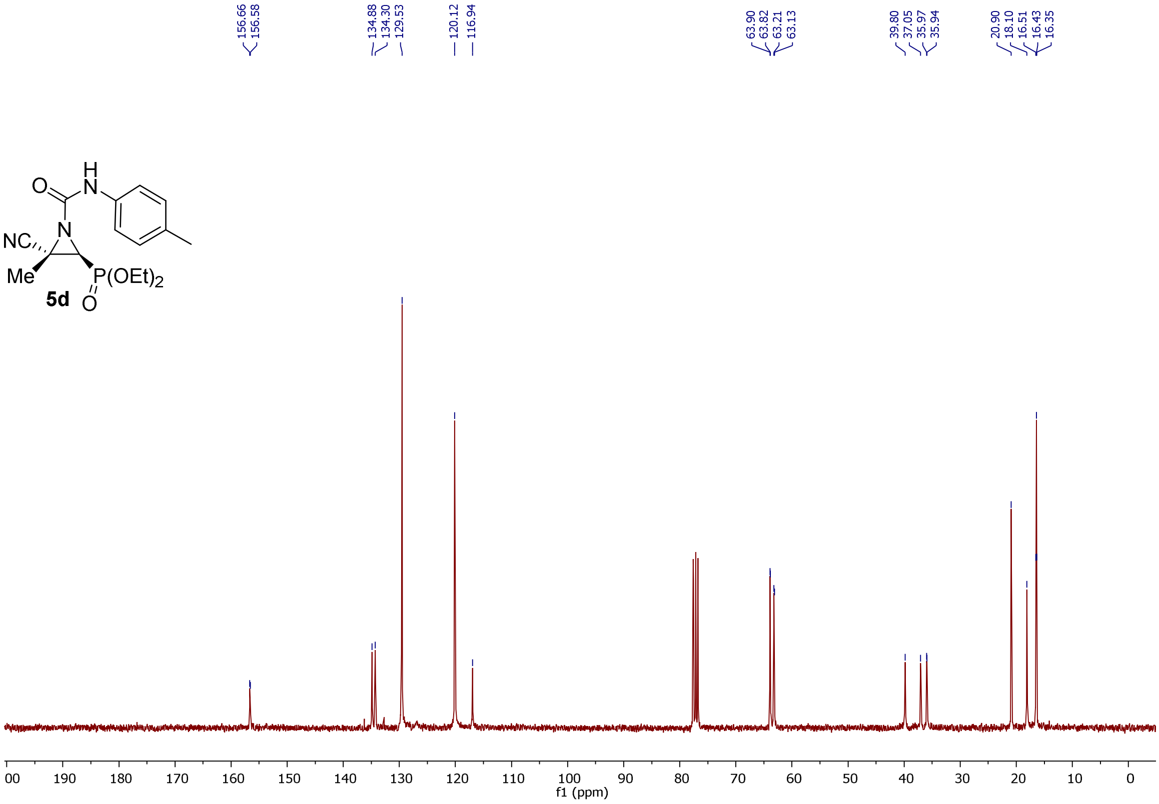

^{1}H NMR (400 MHz, MeOD) of compound **5e**.

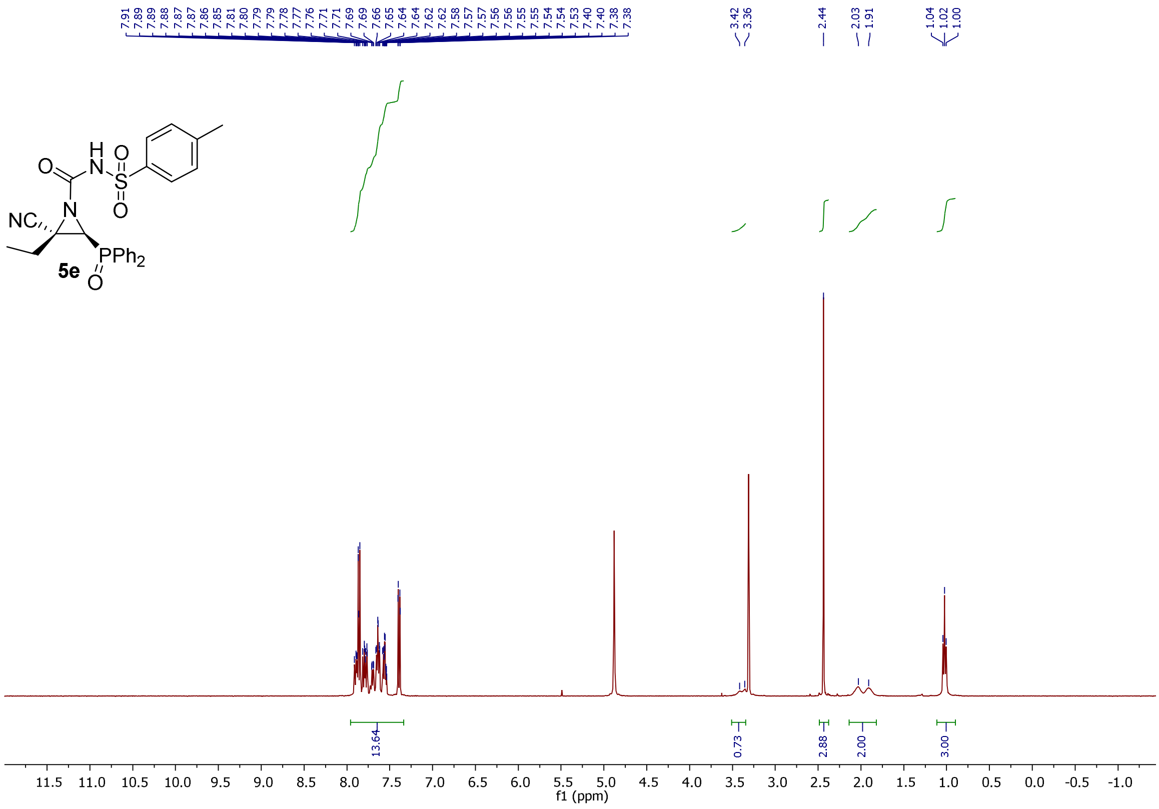

^{13}C {^{1}H} NMR (75 MHz, MeOD) of compound **5e**.

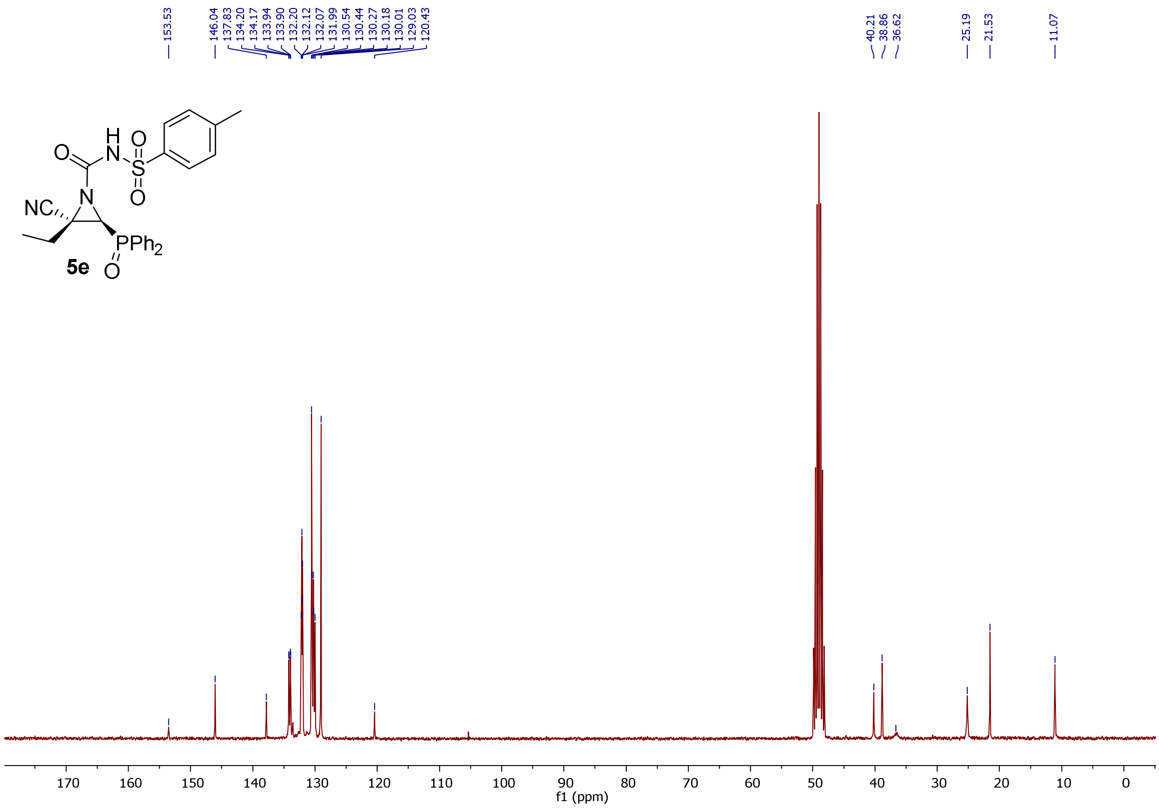

^{1}H NMR (300 MHz, $CDCl_3$) of compound **5f**.

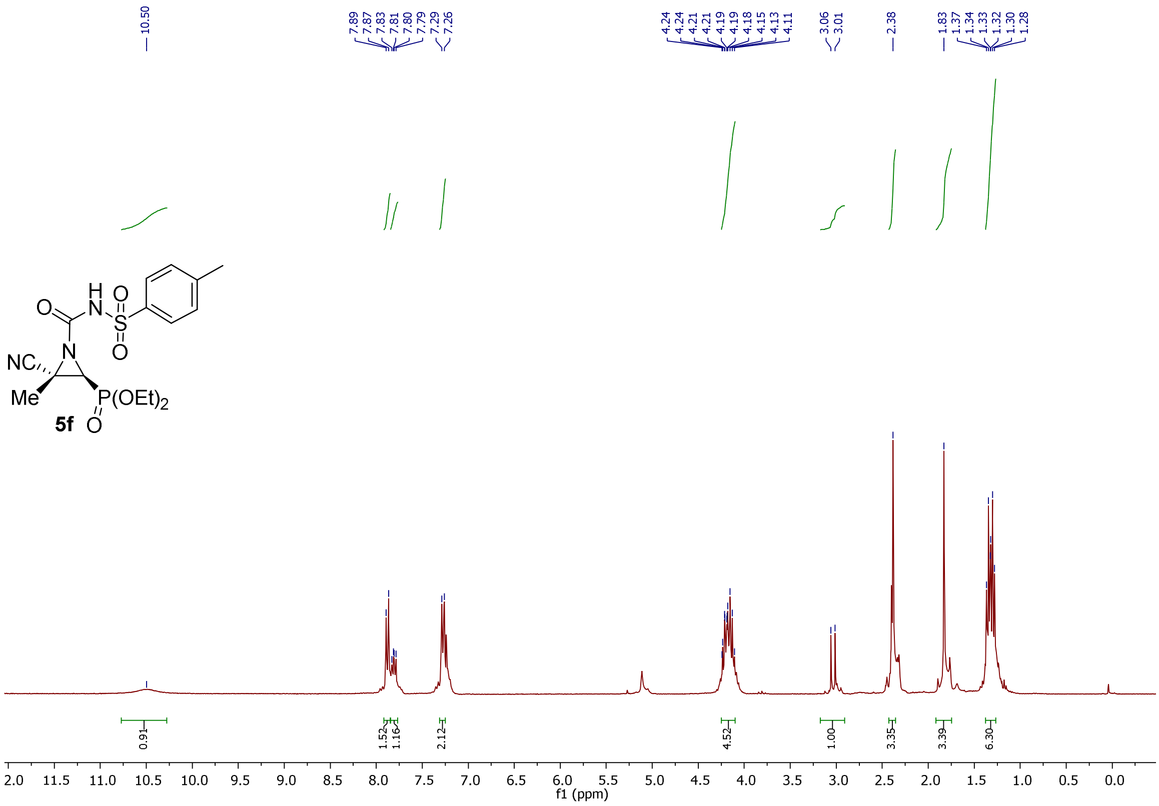

^{13}C {^{1}H} NMR (75 MHz, $CDCl_3$) of compound **5f**.

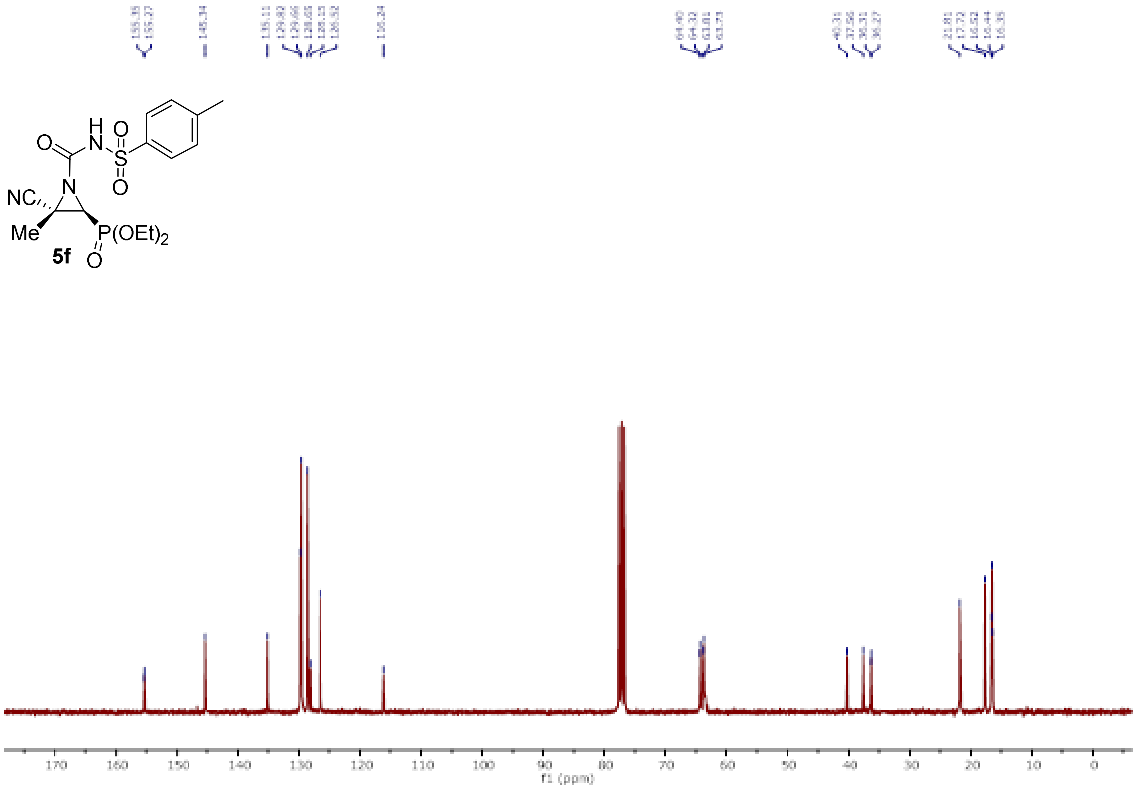

^{1}H NMR (400 MHz, $CDCl_3$) of compound **5g**.

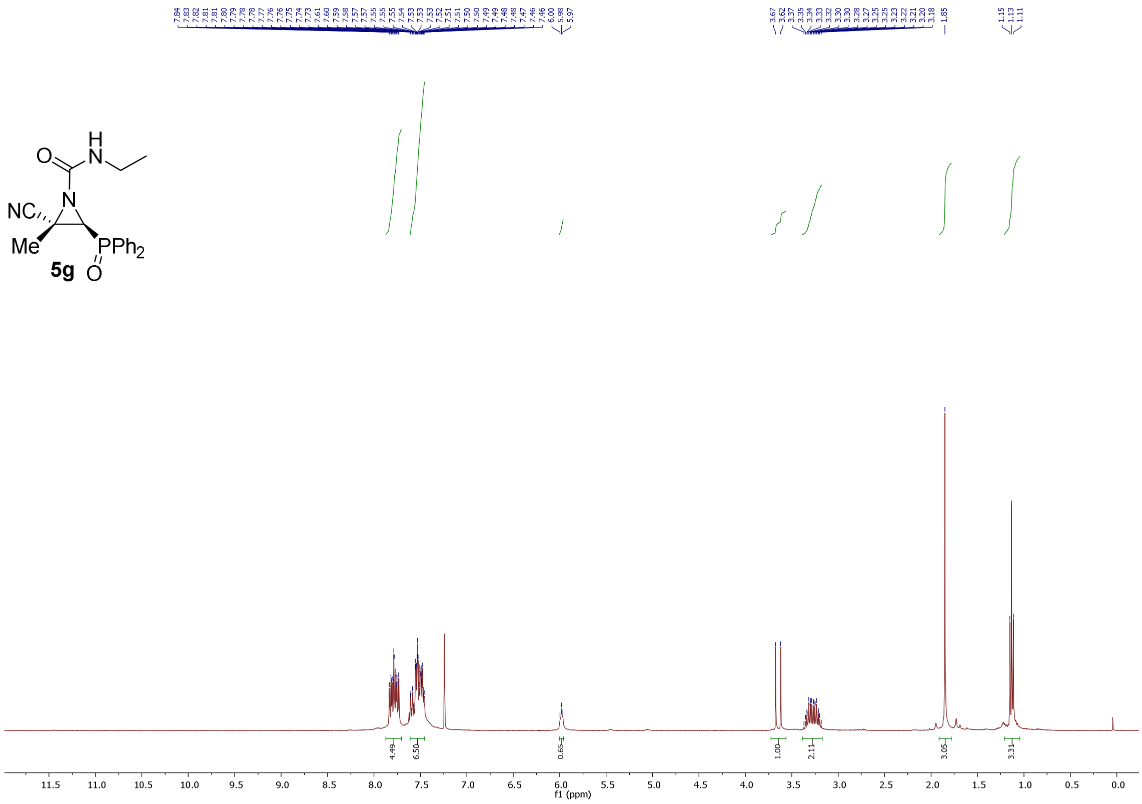

^{13}C {^{1}H} NMR (100 MHz, $CDCl_3$) of compound **5g**.

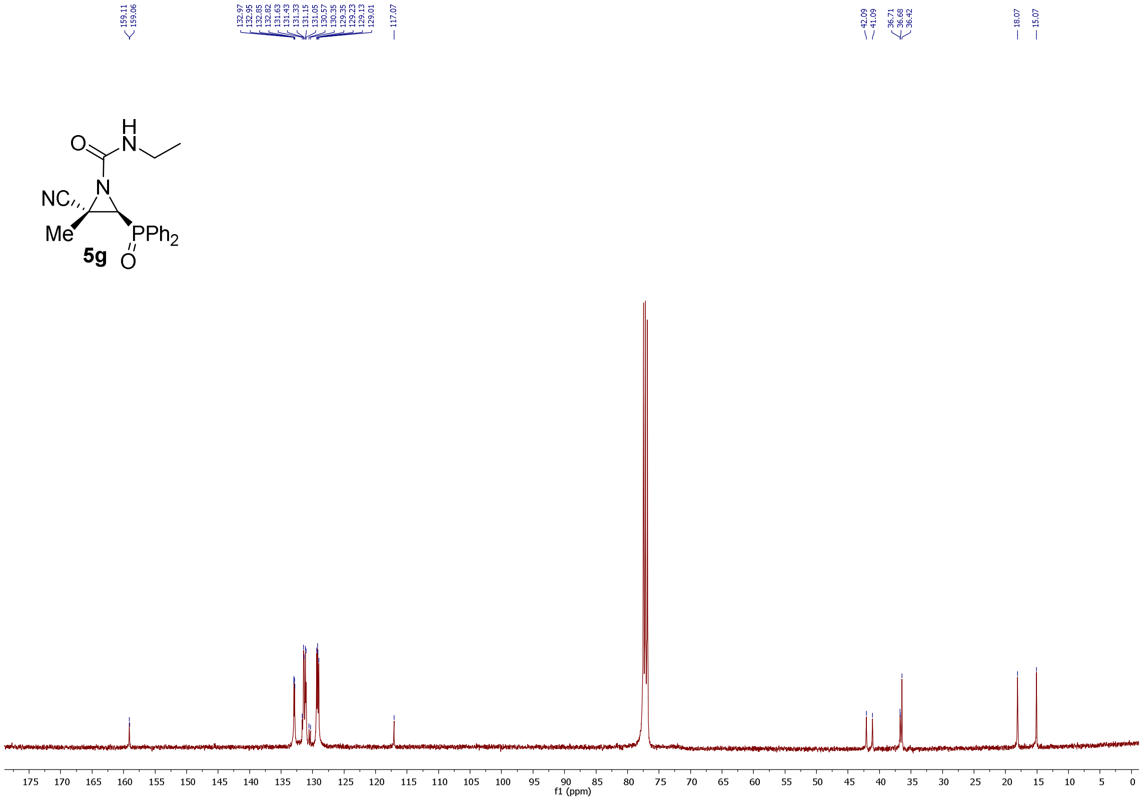

^{1}H NMR (300 MHz, $CDCl_3$) of compound **5h**.

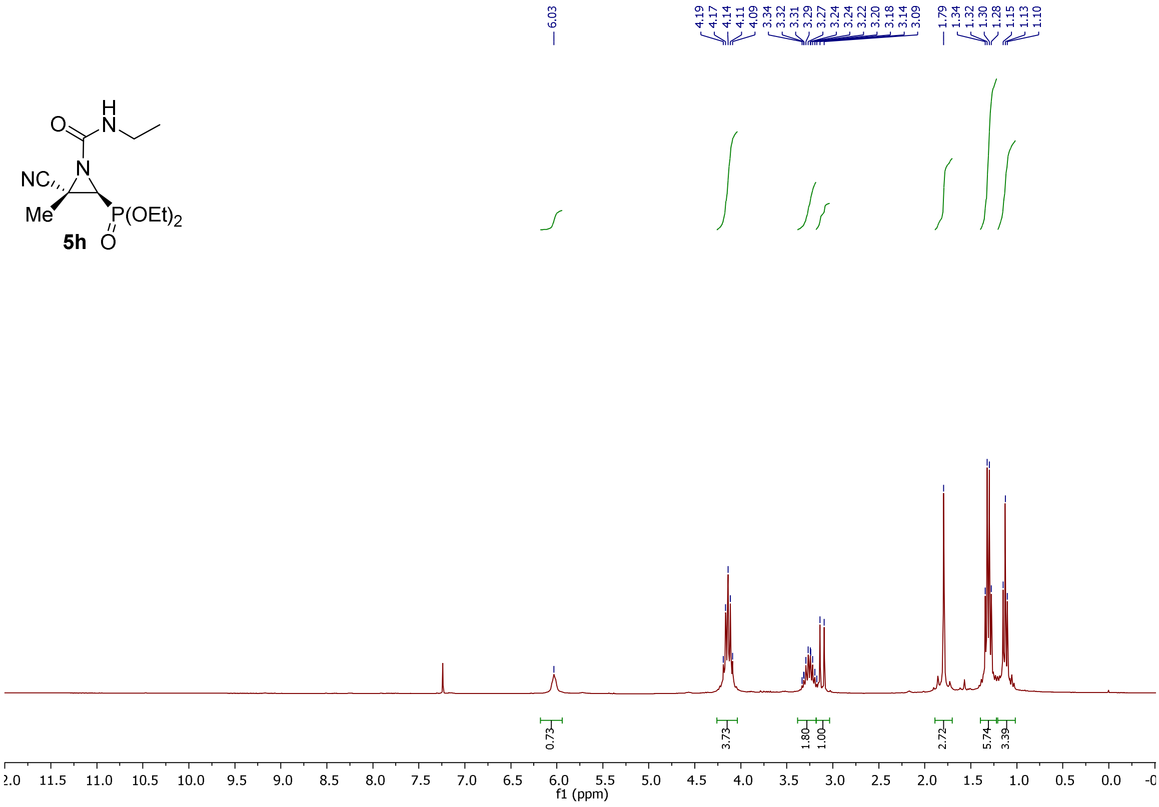

^{13}C {^{1}H} NMR (75 MHz, $CDCl_3$) of compound **5h**.

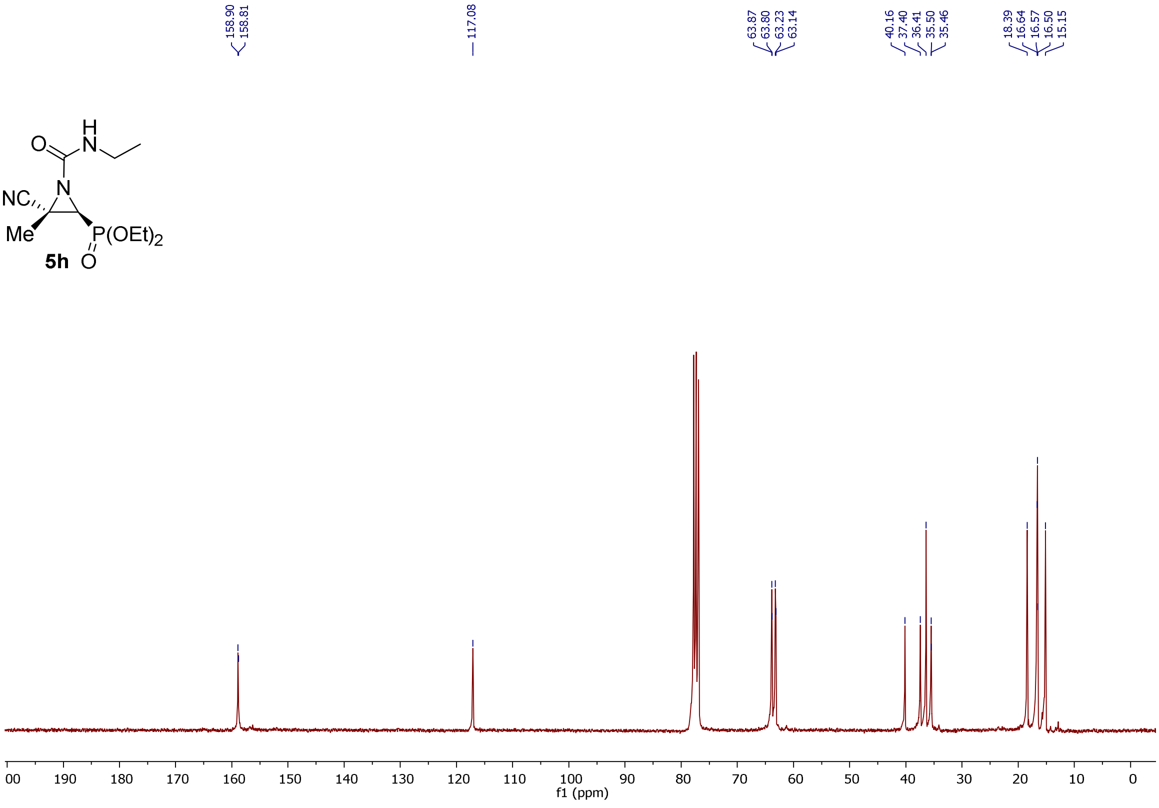

^{1}H NMR (400 MHz, $CDCl_3$) of compound **5i**.

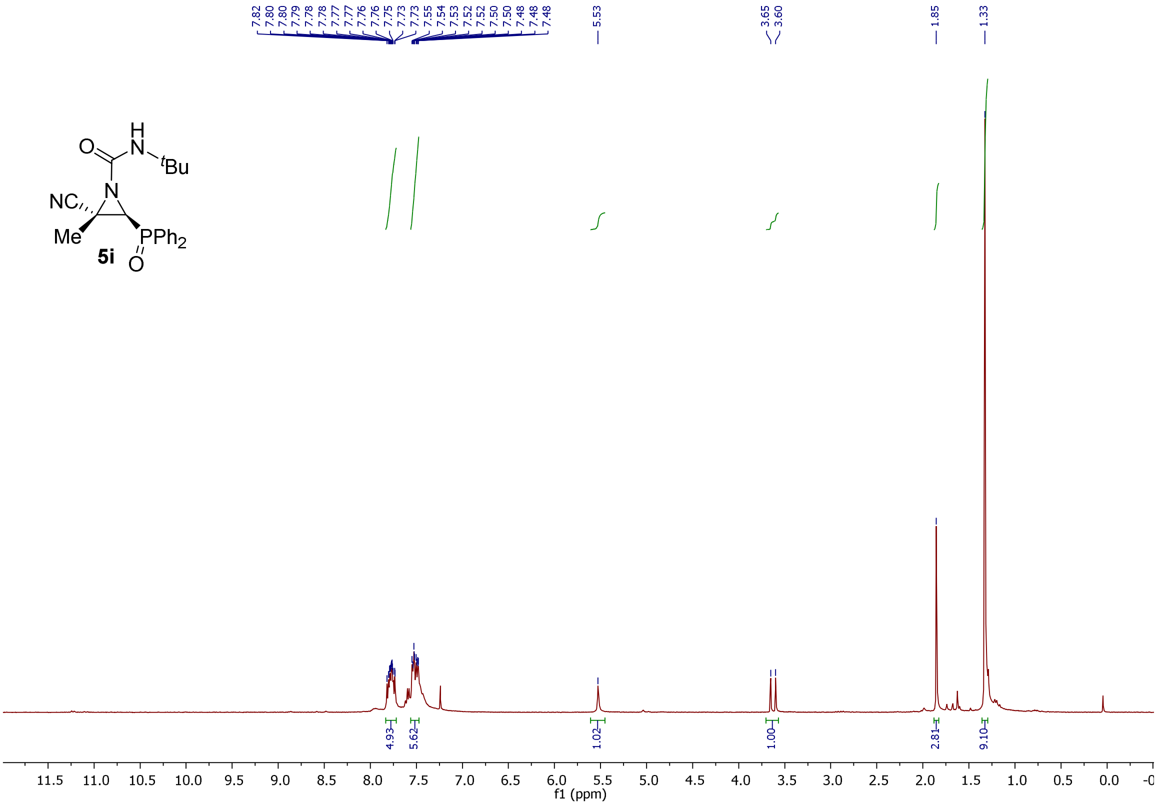

^{13}C {^{1}H} NMR (75 MHz, $CDCl_3$) of compound **5i**.

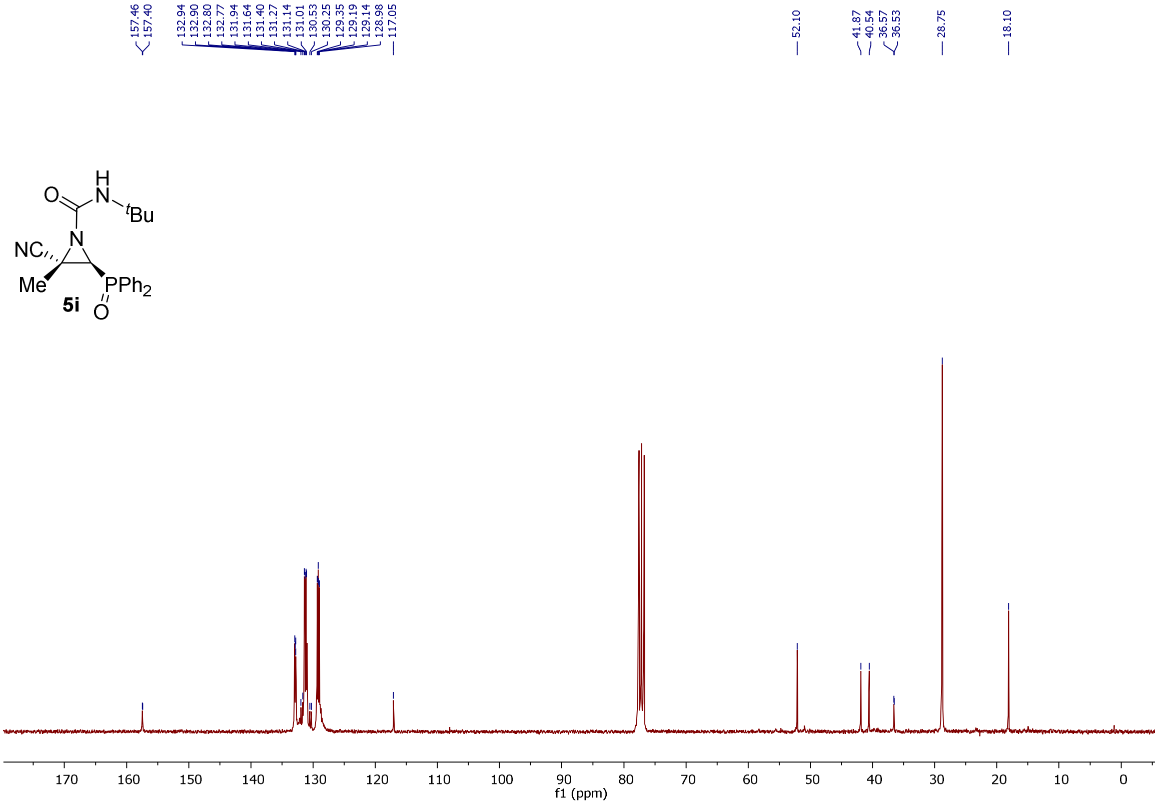

^{1}H NMR (300 MHz, $CDCl_3$) of compound **5j**.

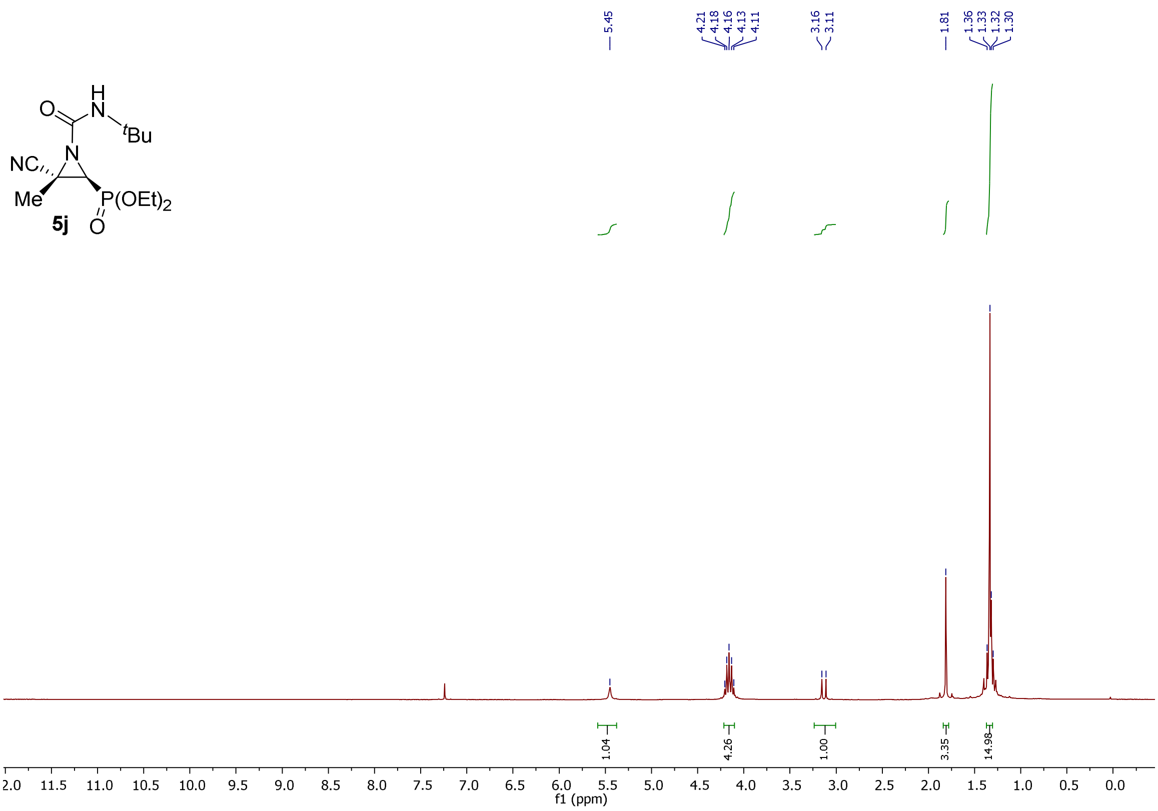

^{13}C {^{1}H} NMR (75 MHz, $CDCl_3$) of compound **5j**.

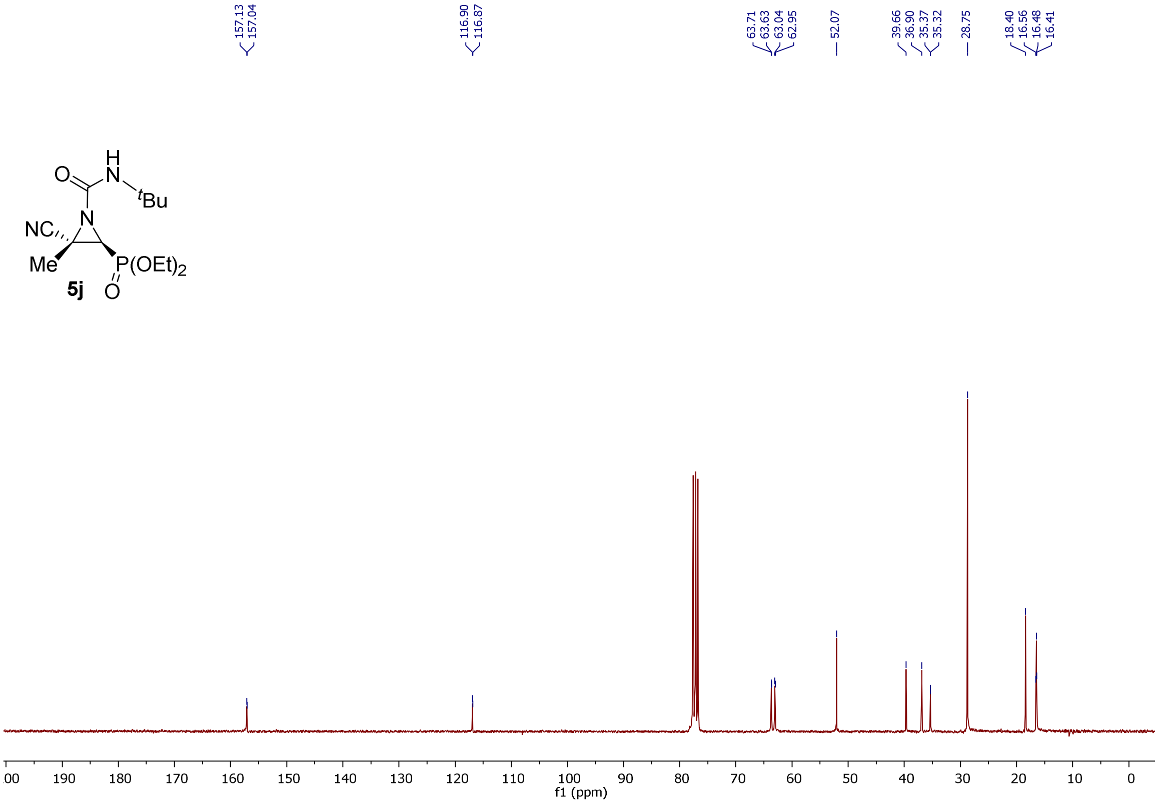

^{1}H NMR (400 MHz, $CDCl_3$) of compound **5k**.

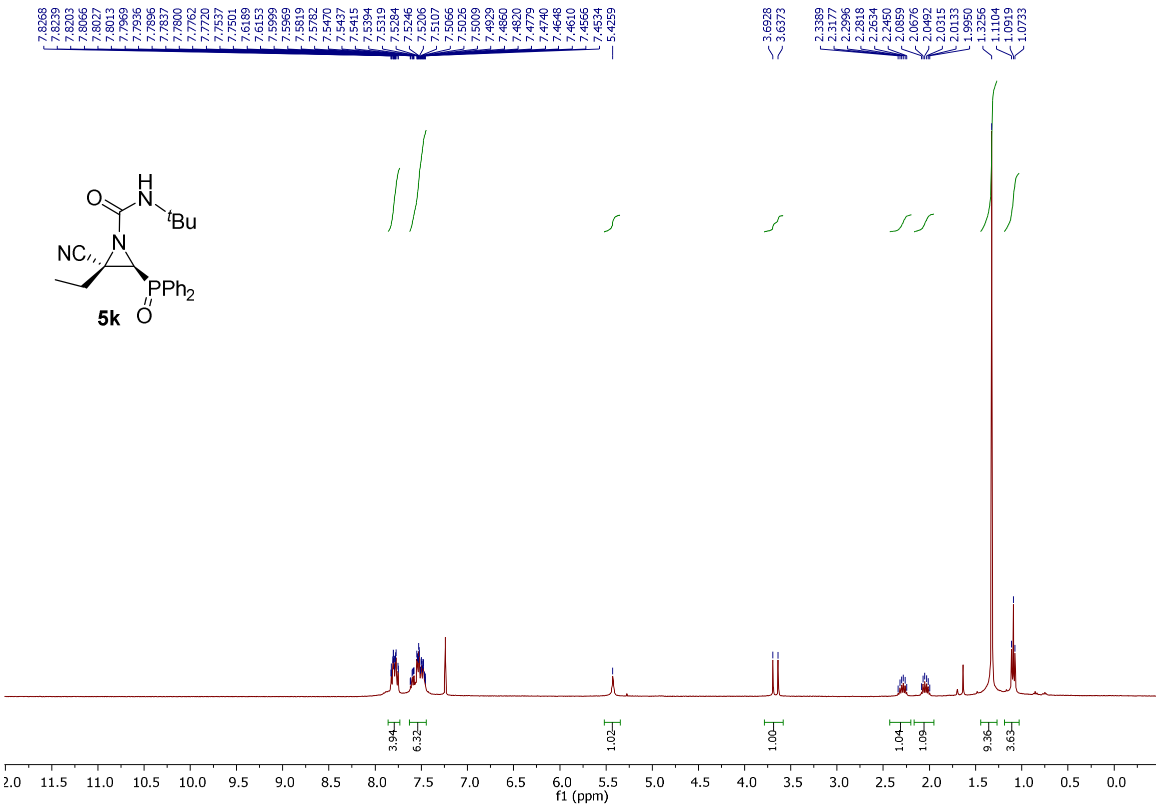

^{13}C {^{1}H} NMR (100 MHz, $CDCl_3$) of compound **5k**.

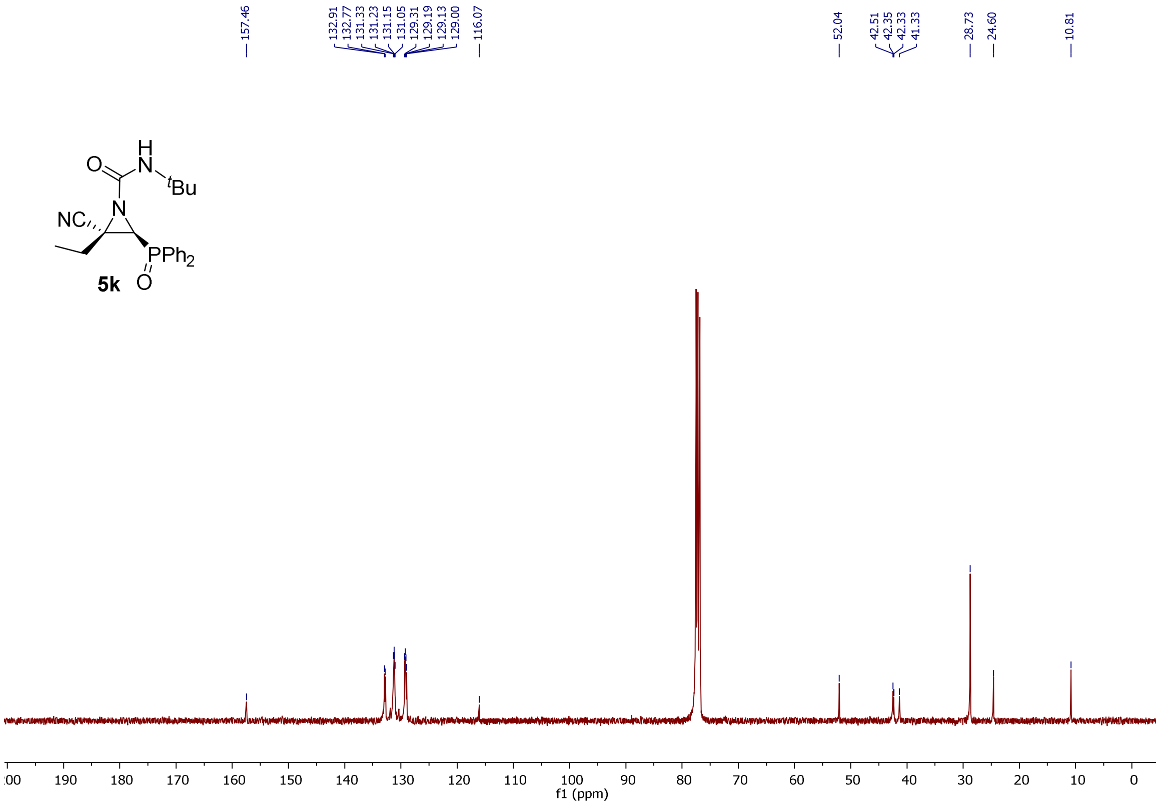

^{1}H NMR (400 MHz, $CDCl_3$) of compound **6a**.

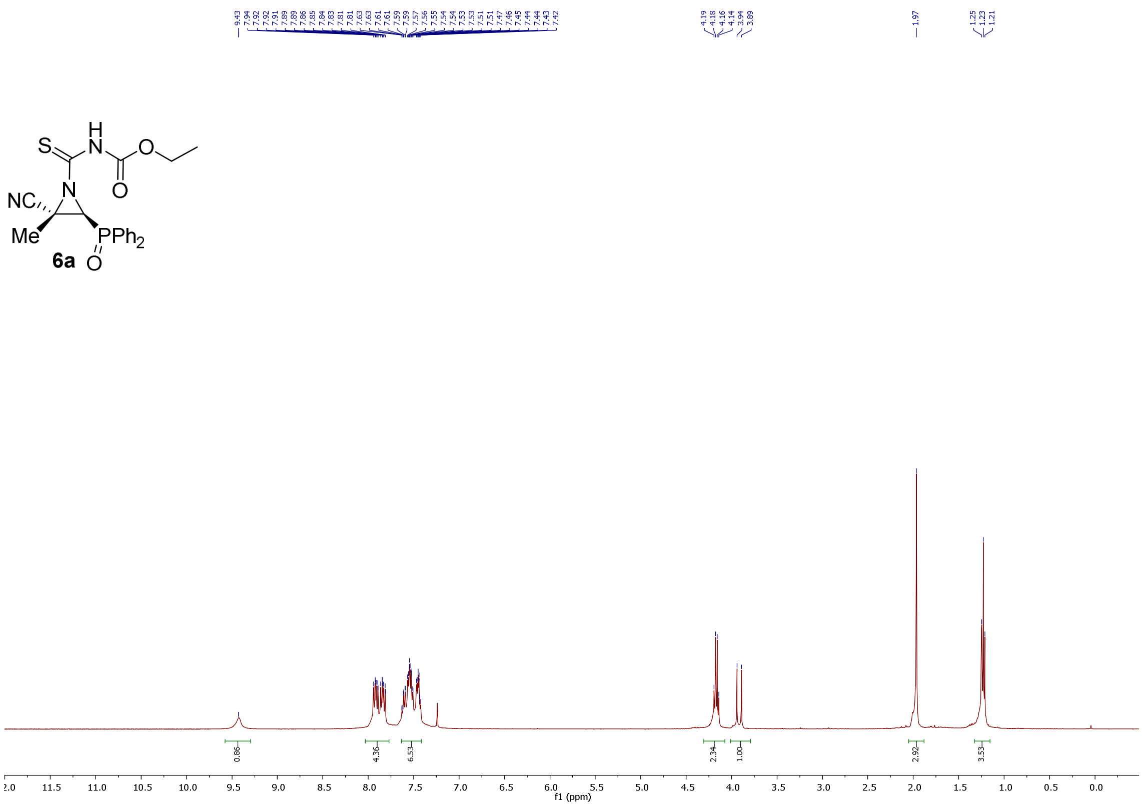

^{13}C {^{1}H} NMR (75 MHz, $CDCl_3$) of compound **6a**.

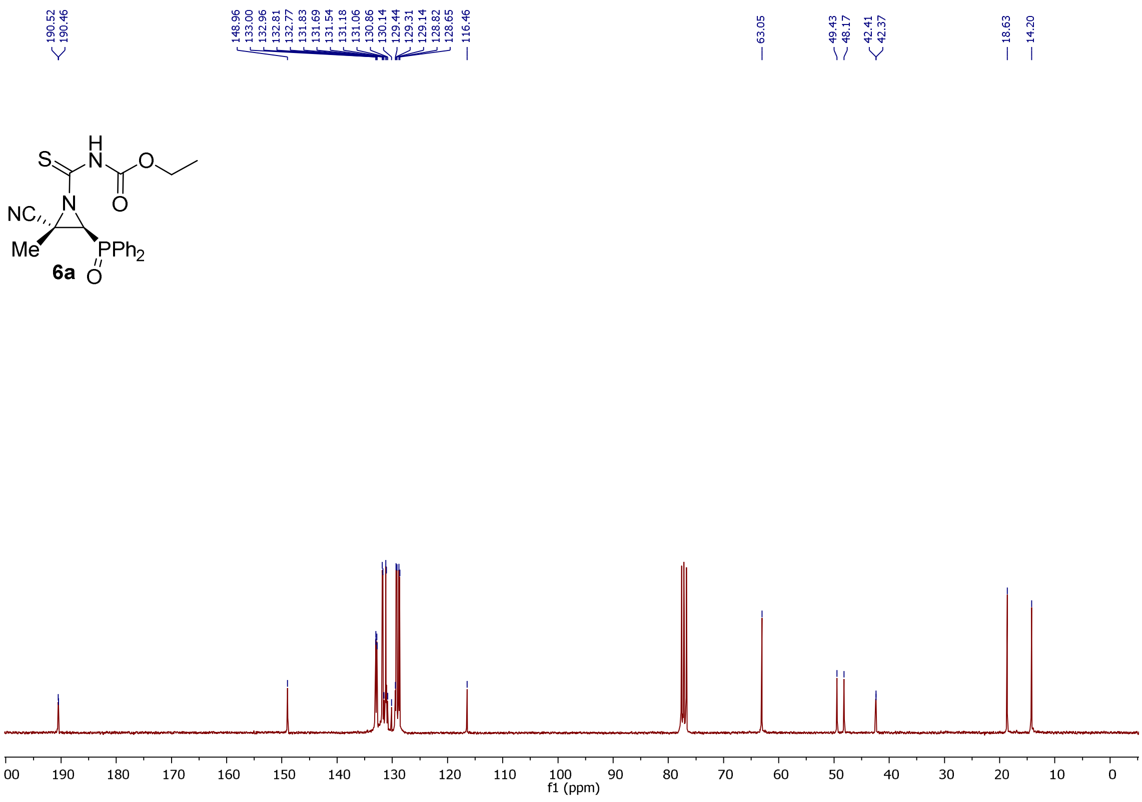

¹H NMR (400 MHz, CDCl₃) of compound **6b**.

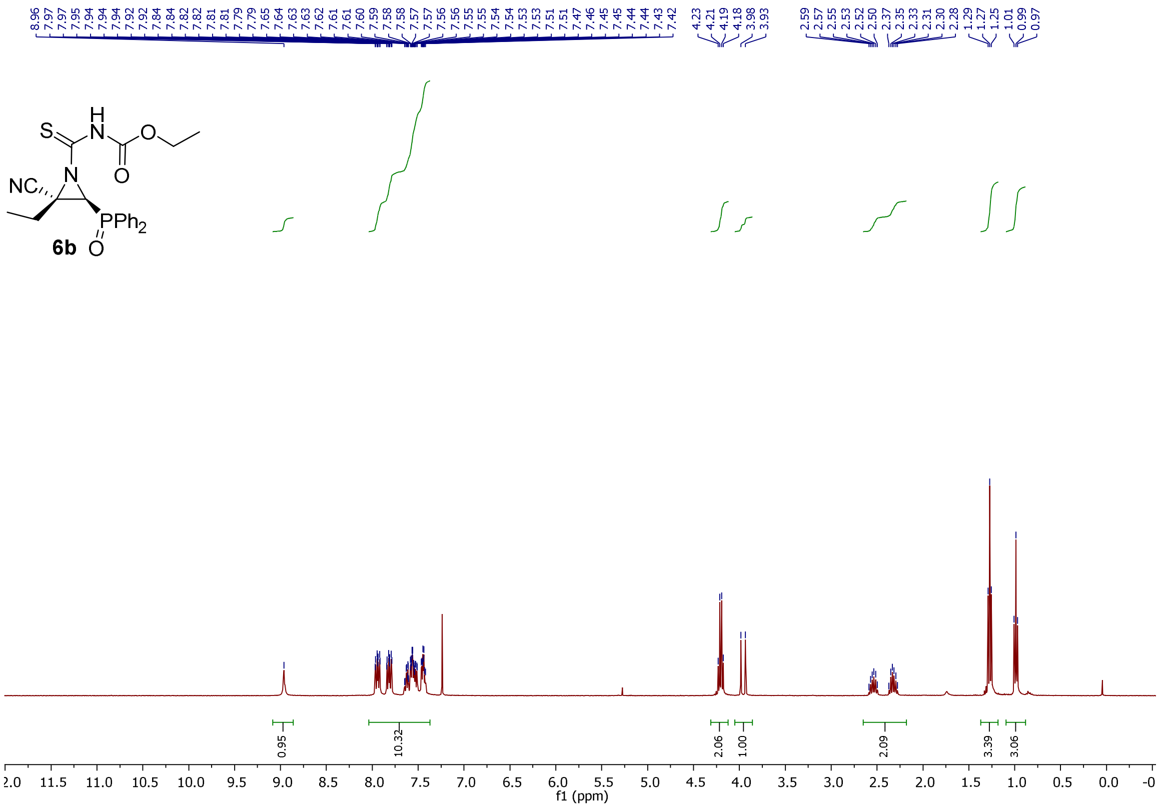

¹³C {¹H} NMR (75 MHz, CDCl₃) of compound **6b**.

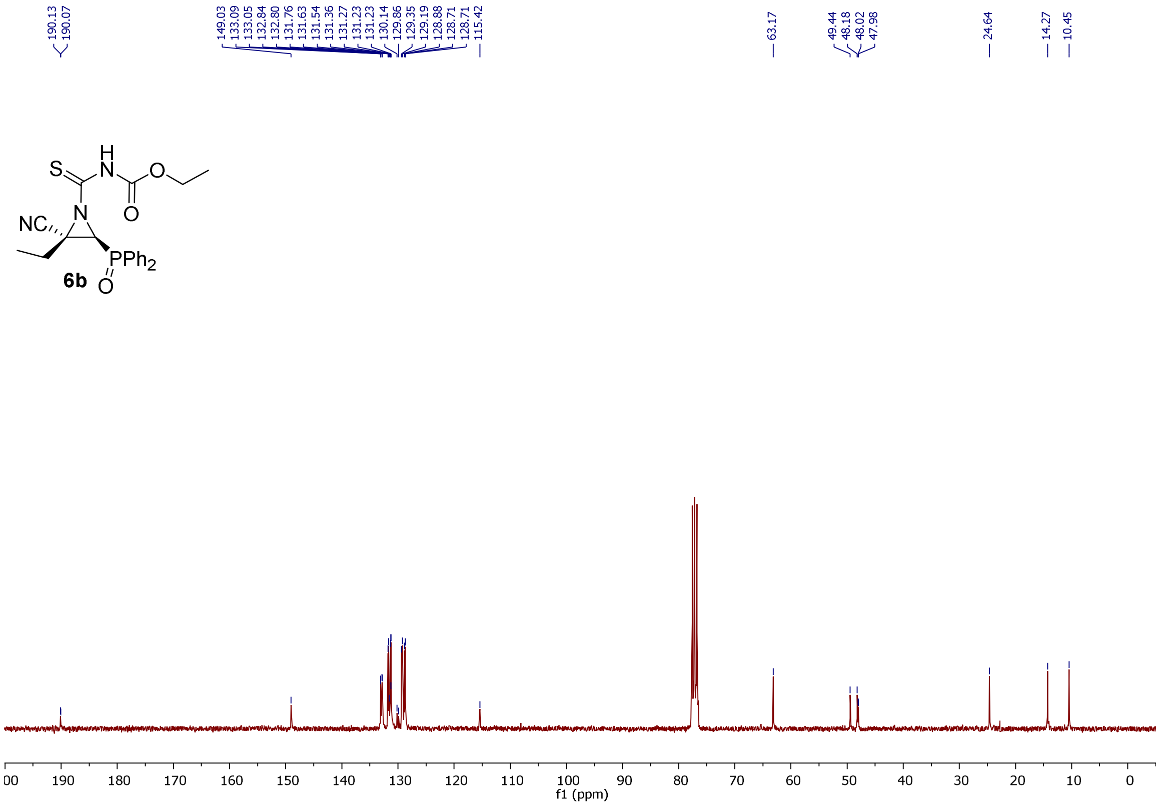

^{1}H NMR (300 MHz, $CDCl_3$) of compound **6c**.

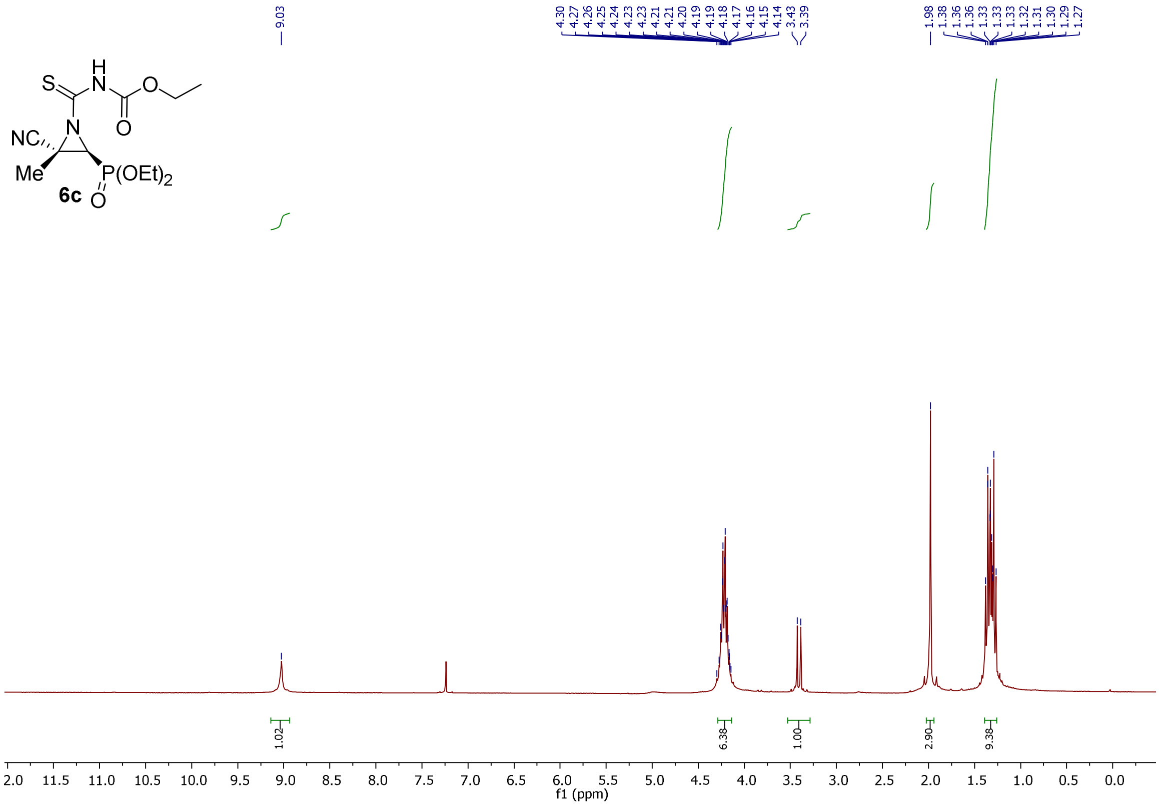

^{13}C {^{1}H} NMR (75 MHz, $CDCl_3$) of compound **6c**.

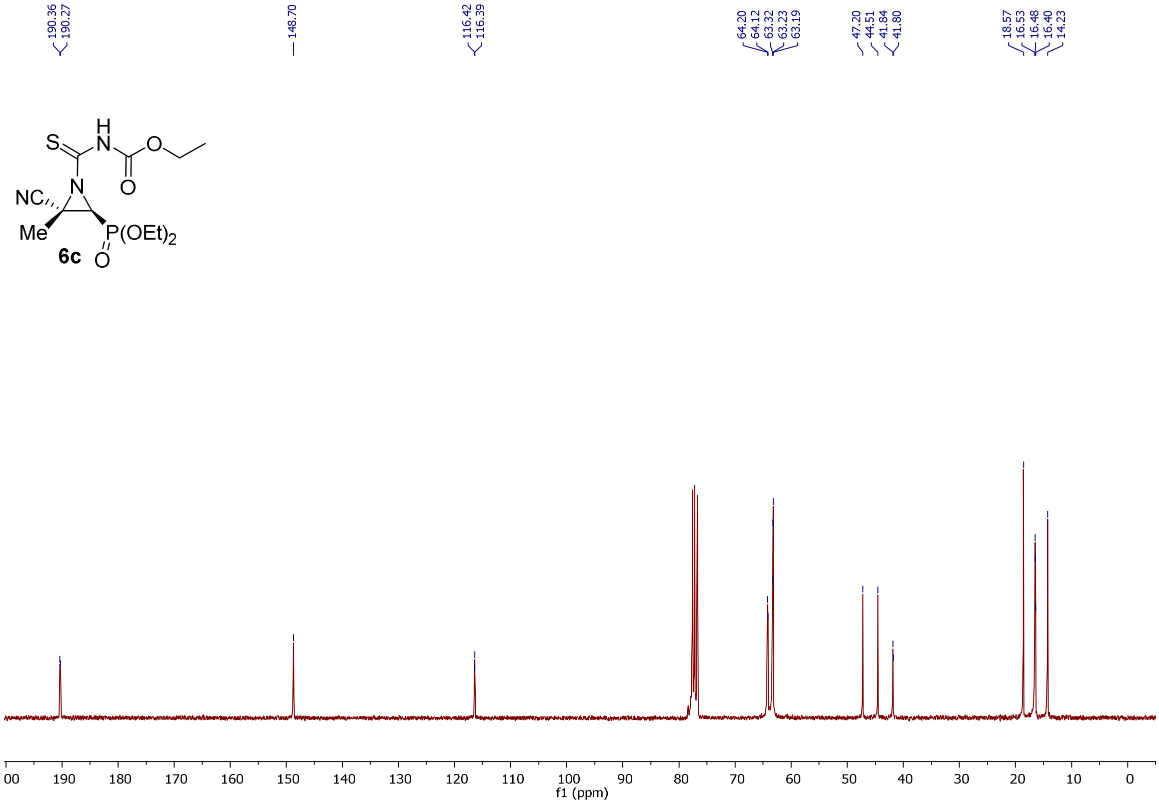

^{1}H NMR (400 MHz, $CDCl_3$) of compound **7a** or **7′a**.

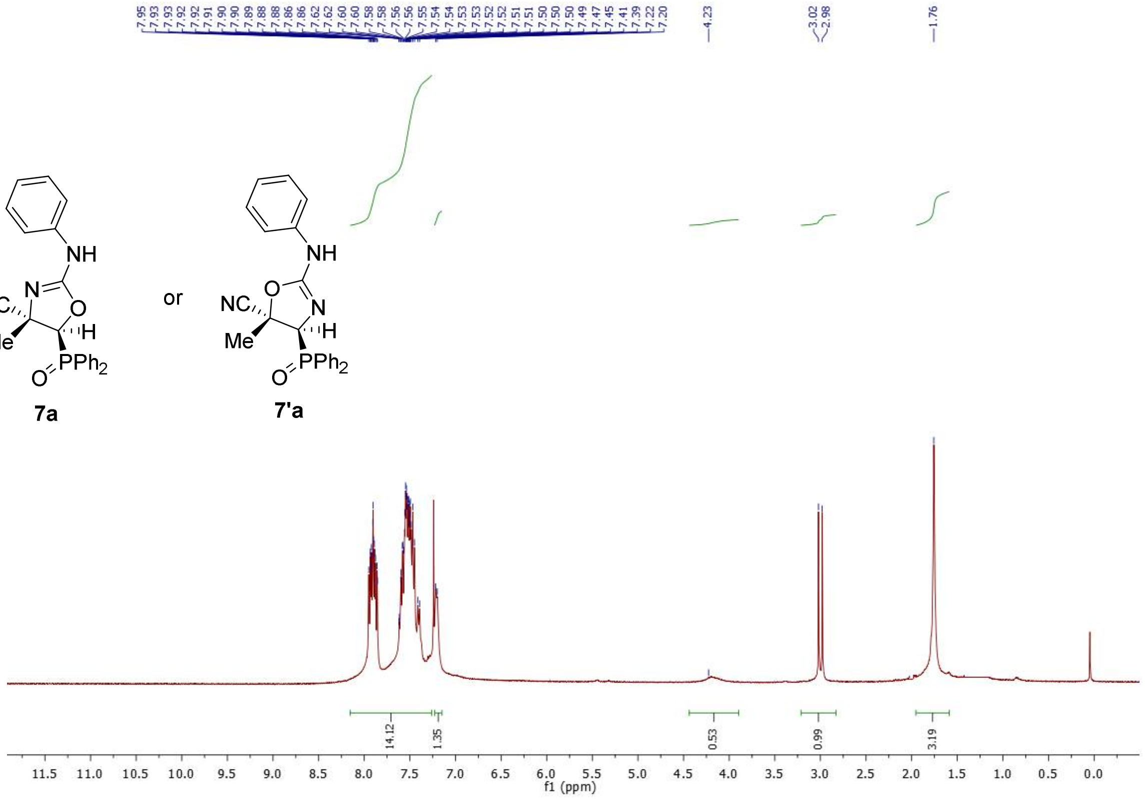

^{13}C {^{1}H} NMR (75 MHz, $CDCl_3$) of compound **7a** or **7′a**.

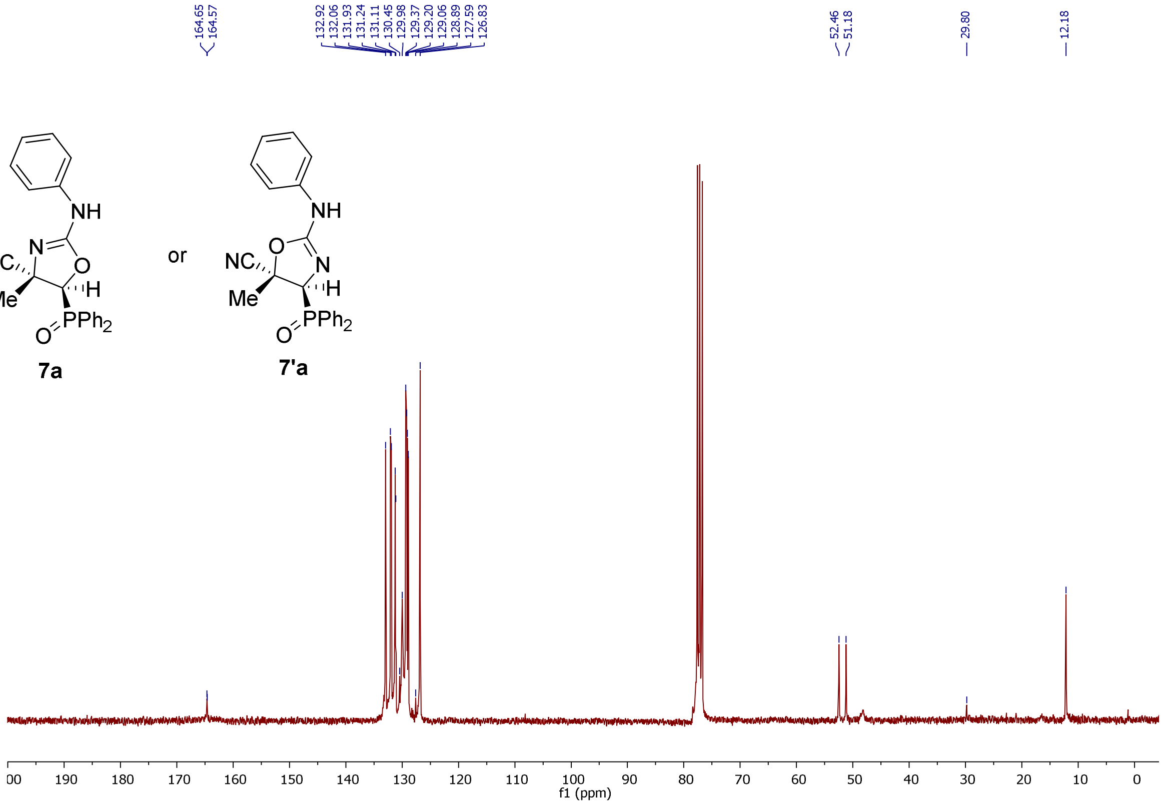

^{1}H NMR (300 MHz, CDCl$_3$) of compound **7b + 7′b**.

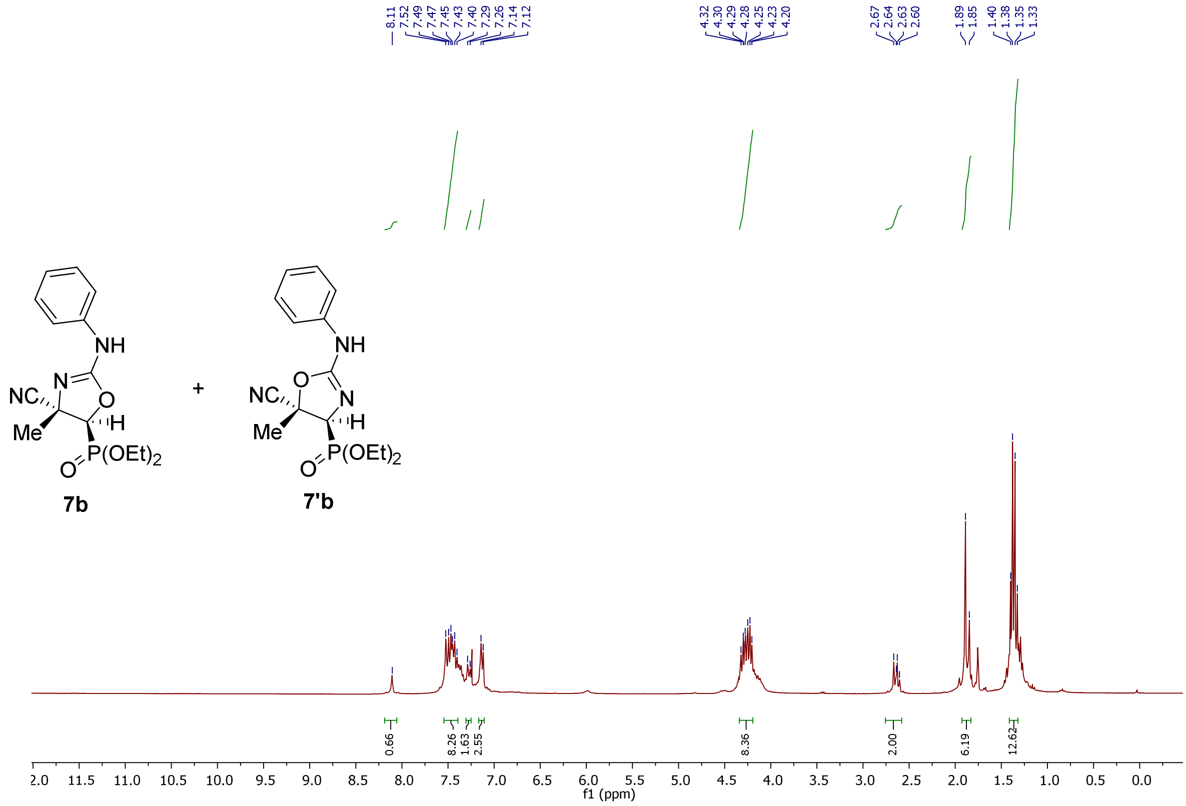

^{13}C {^{1}H} NMR (75 MHz, CDCl$_3$) of compound **7b + 7′b**.

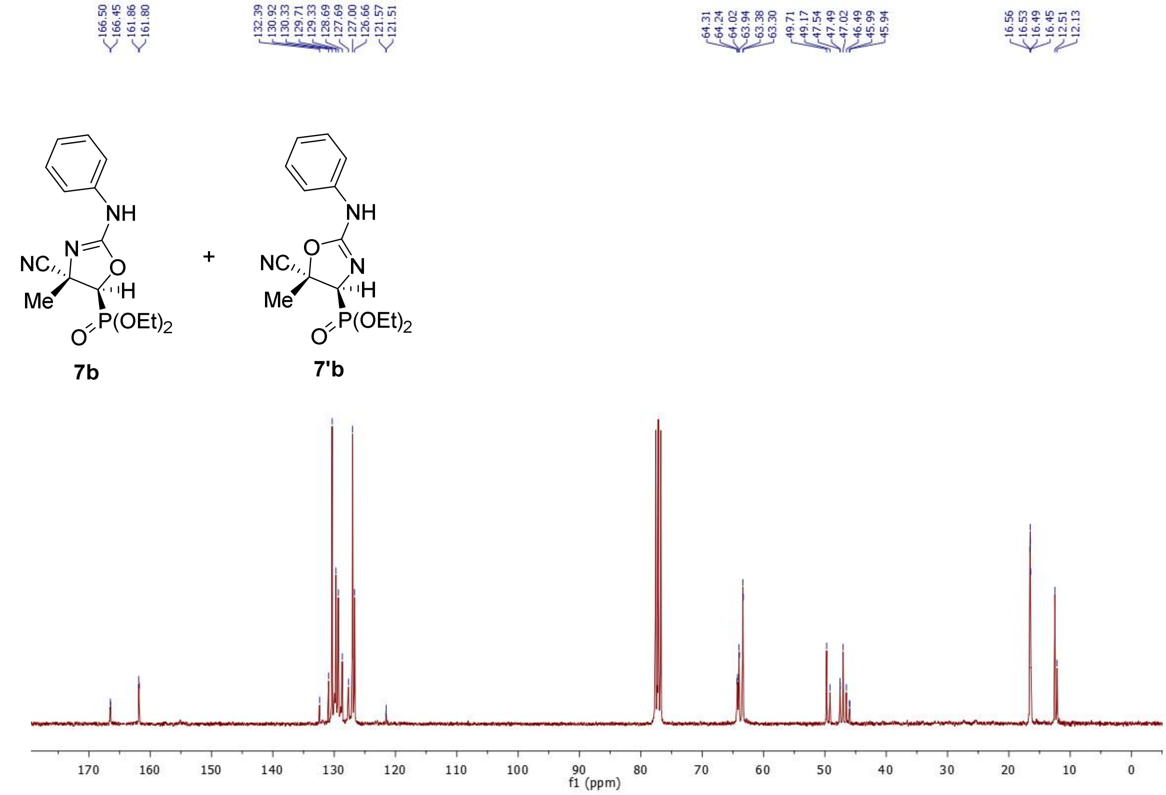

^{1}H NMR (400 MHz, $CDCl_3$) of compound **7c + 7′c**.

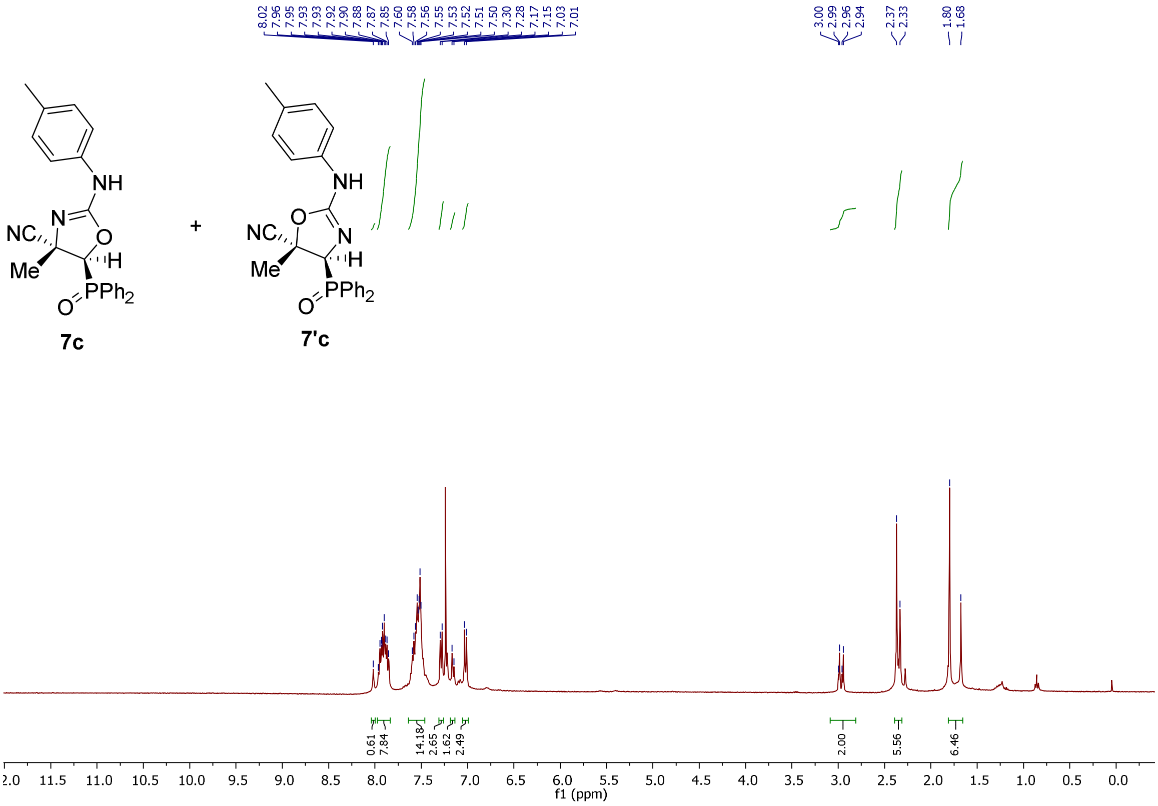

^{13}C {^{1}H} NMR (75 MHz, $CDCl_3$) of compound **7c + 7′c**.

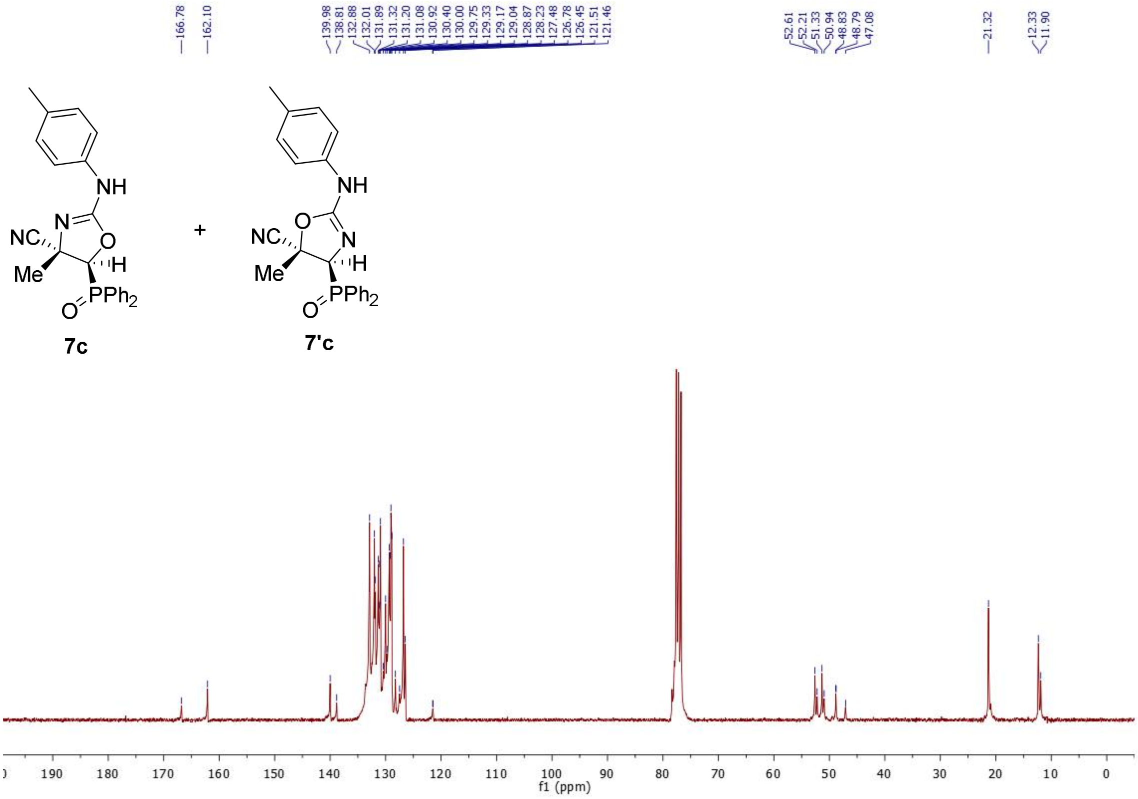

^{1}H NMR (300 MHz, $CDCl_3$) of compound **7d + 7’d**.

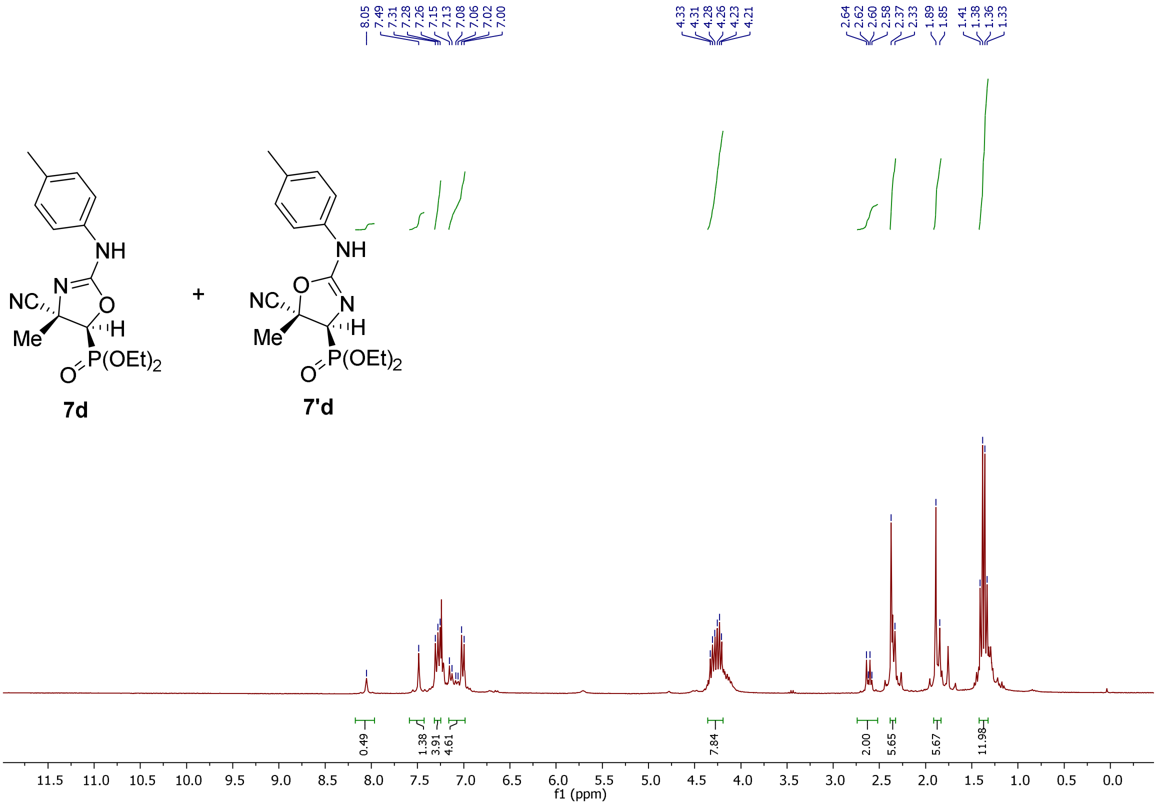

^{13}C {^{1}H} NMR (75 MHz, $CDCl_3$) of compound **7d + 7’d**.

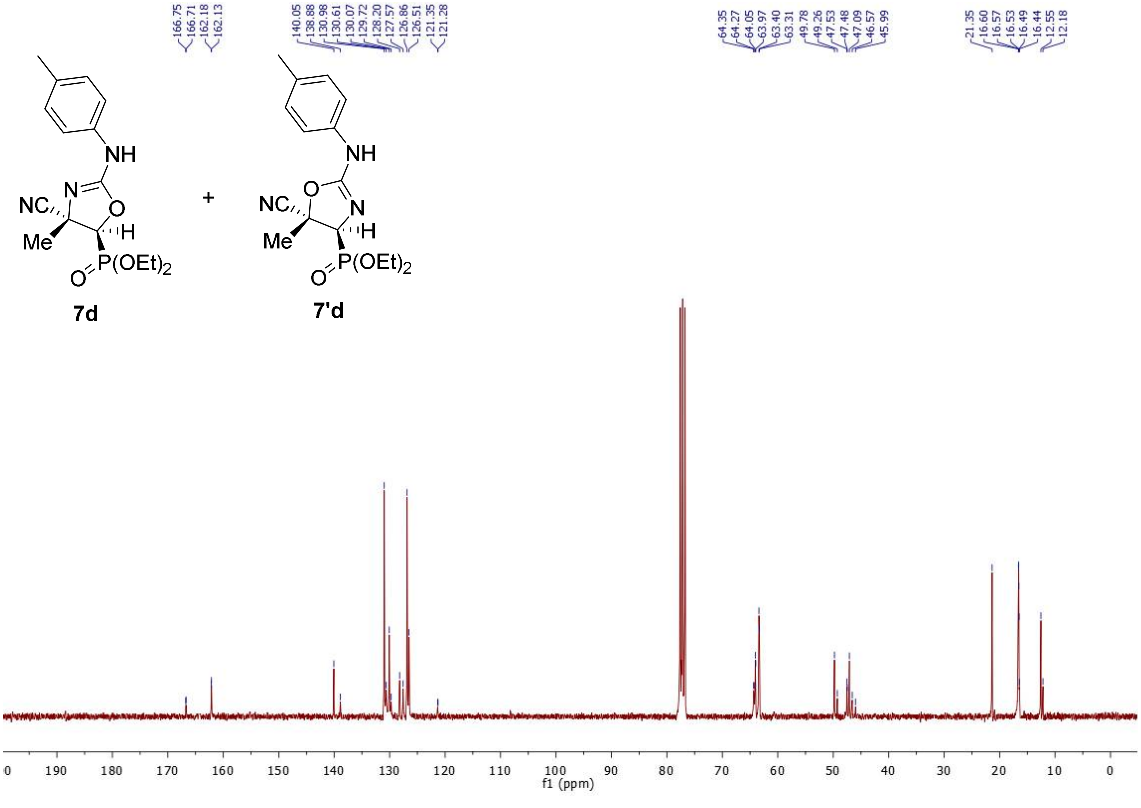

^{1}H NMR (400 MHz, $CDCl_3$) of compound **8a**.

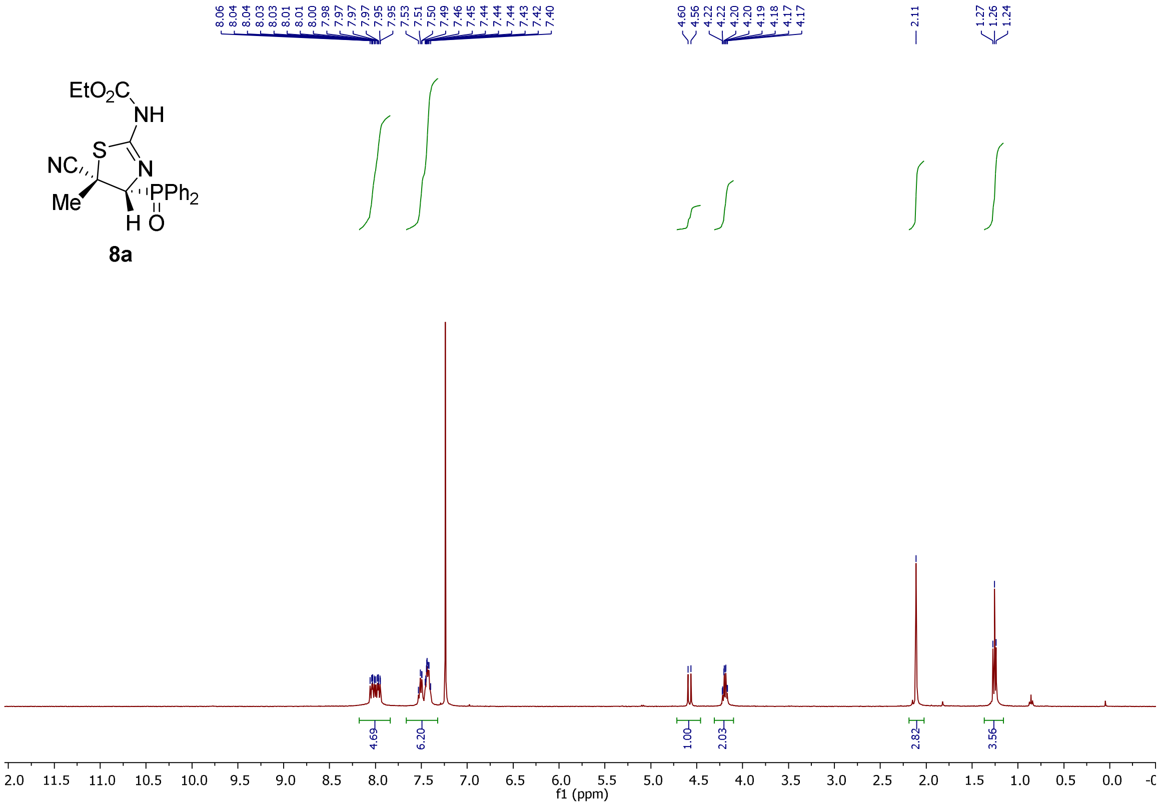

^{13}C {^{1}H} NMR (75 MHz, $CDCl_3$) of compound **8a**.

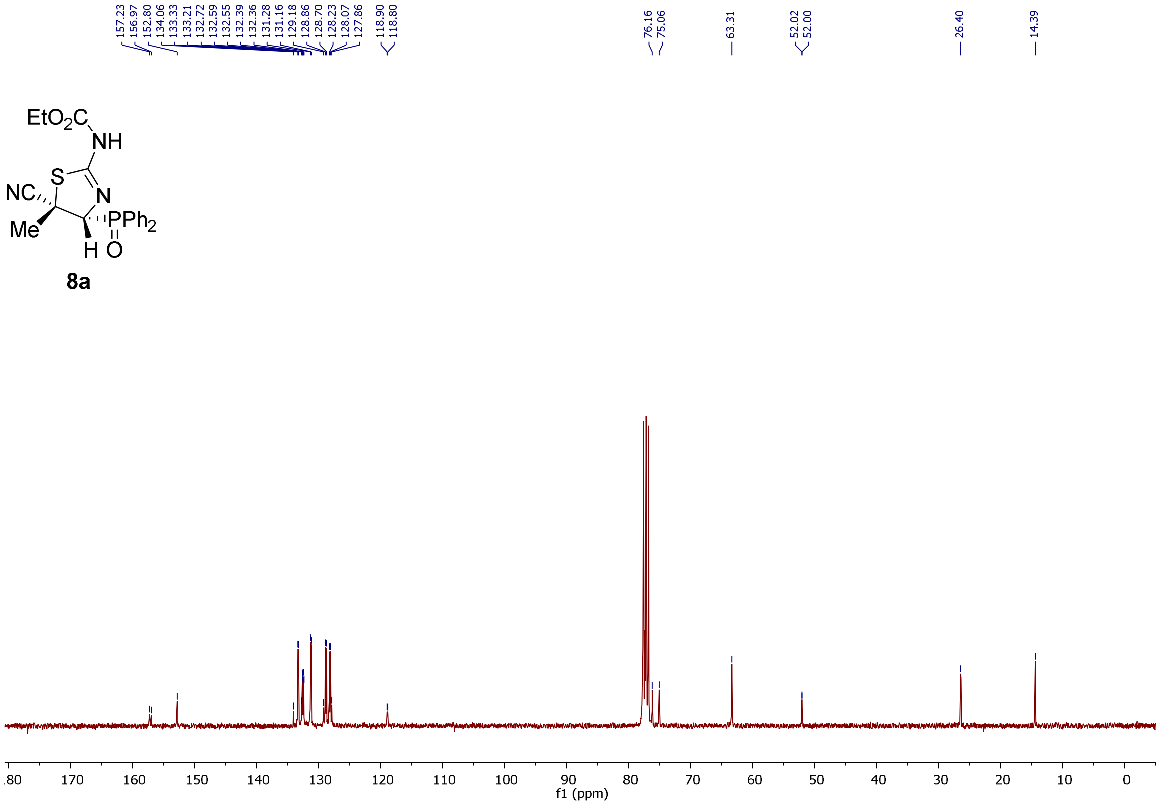

^{1}H NMR (300 MHz, $CDCl_3$) of compound **9a**.

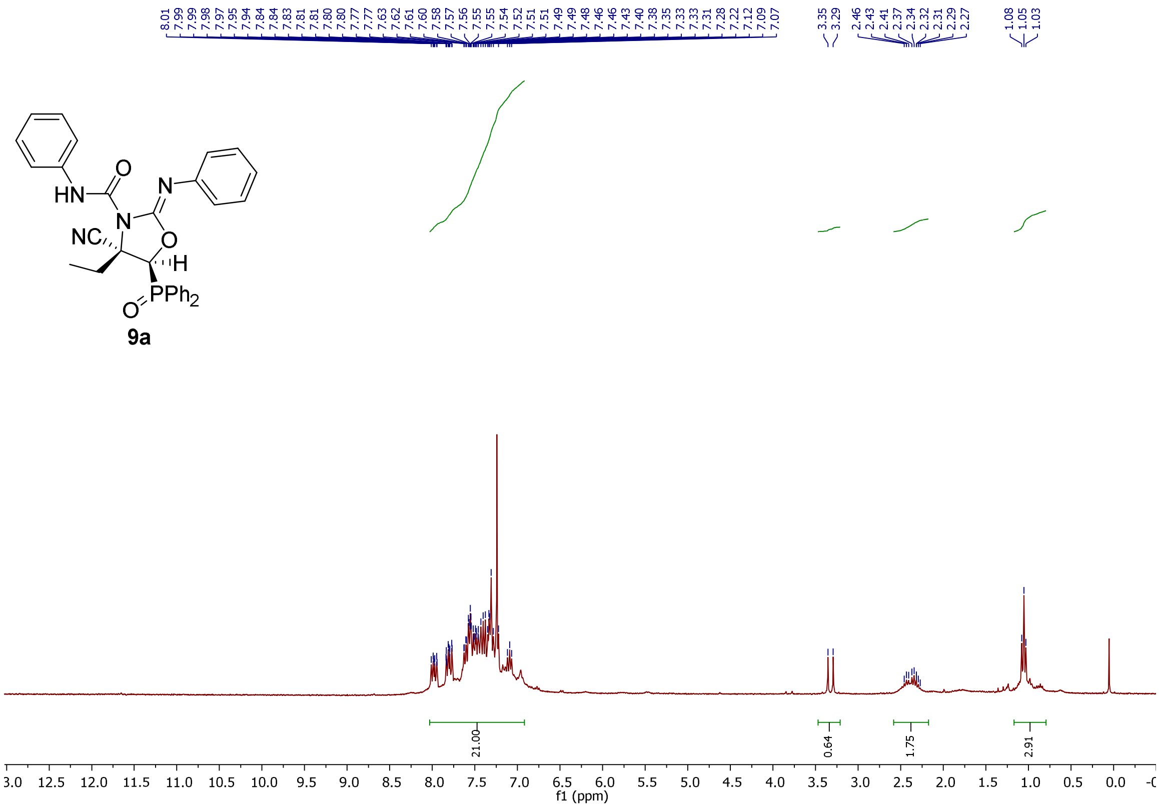

^{13}C {^{1}H} NMR (75 MHz, $CDCl_3$) of compound **9a**.

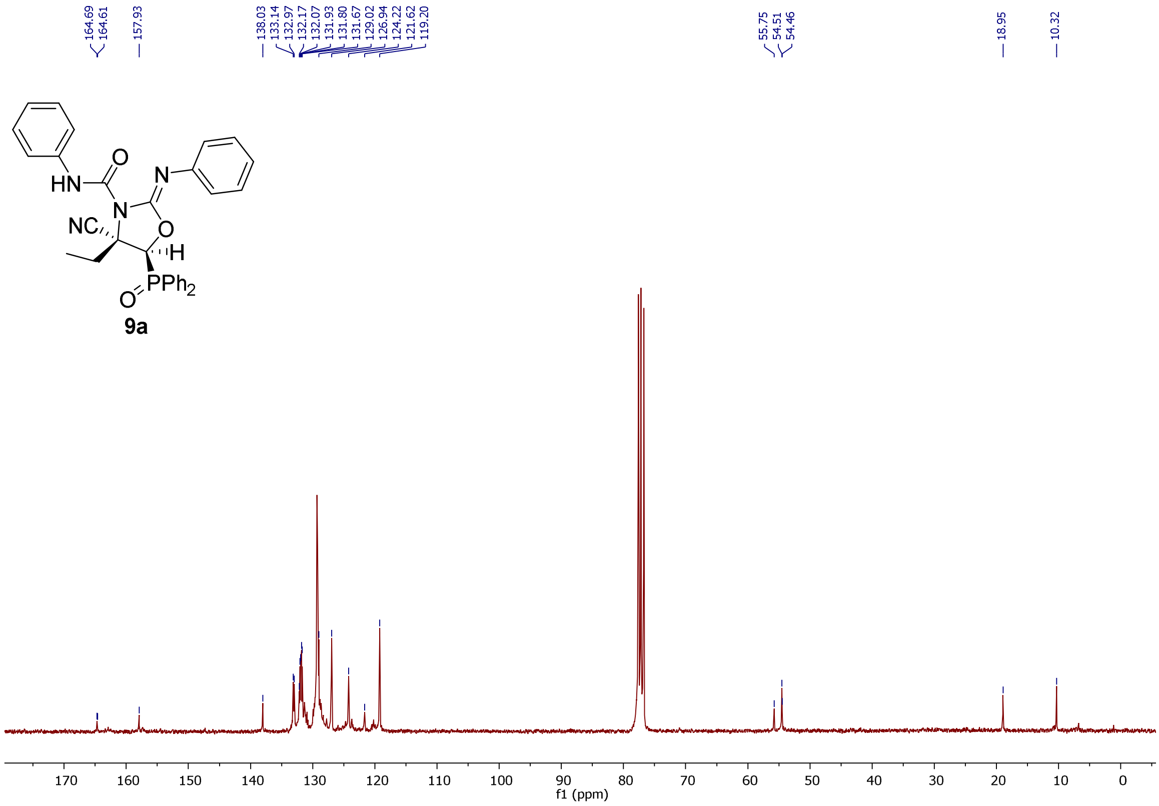

^{1}H NMR (300 MHz, $CDCl_3$) of compound **9b**.

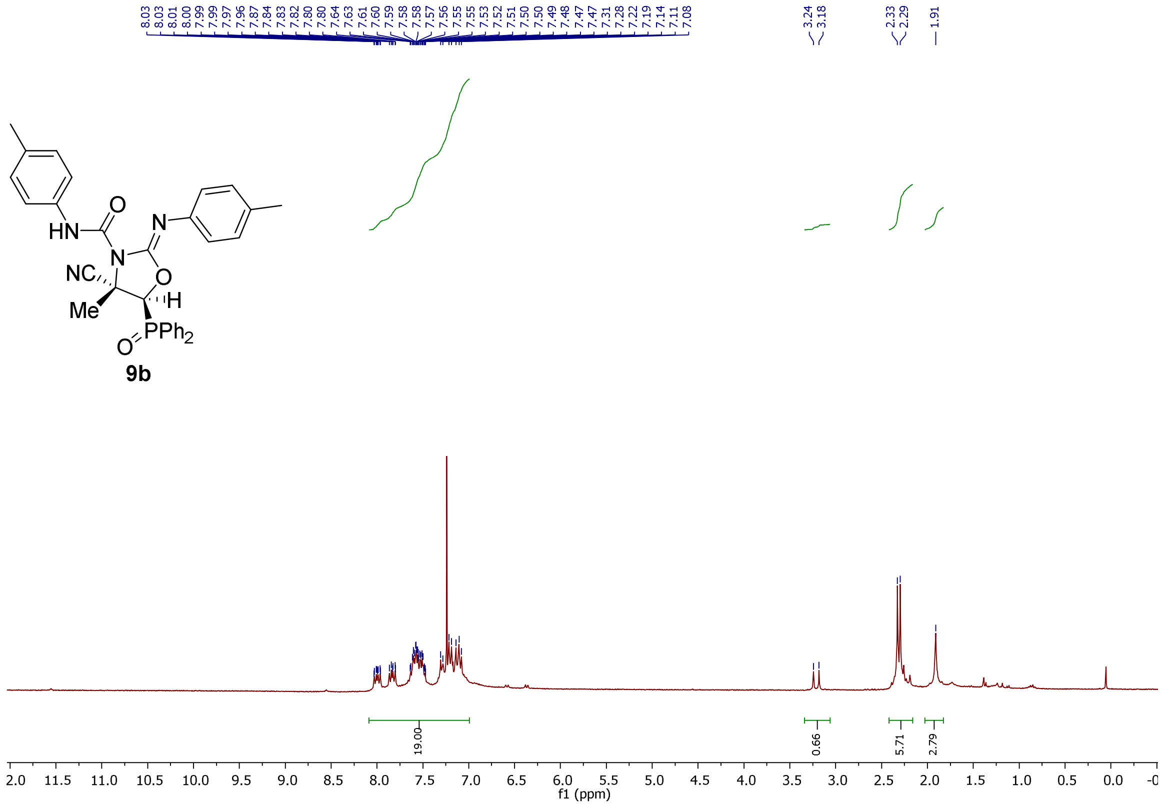

^{13}C {^{1}H} NMR (75 MHz, $CDCl_3$) of compound **9b**.

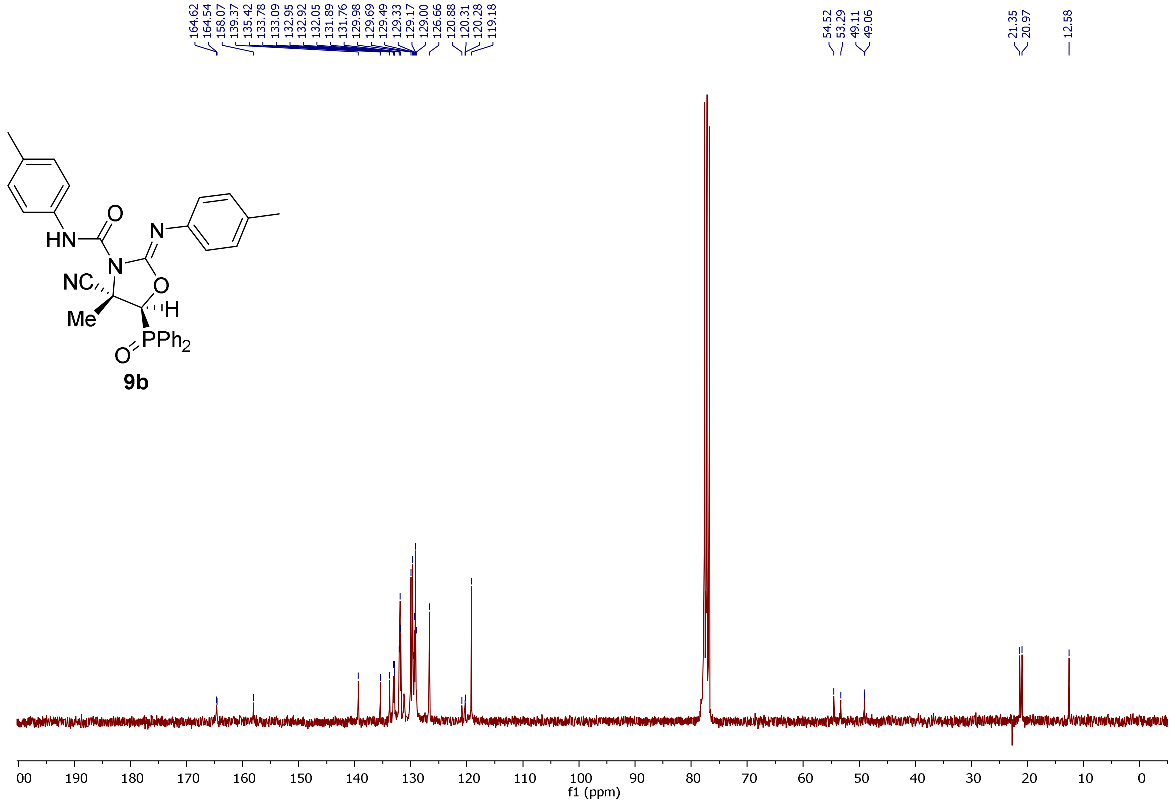

^{1}H NMR (300 MHz, $CDCl_3$) of compound **9c**.

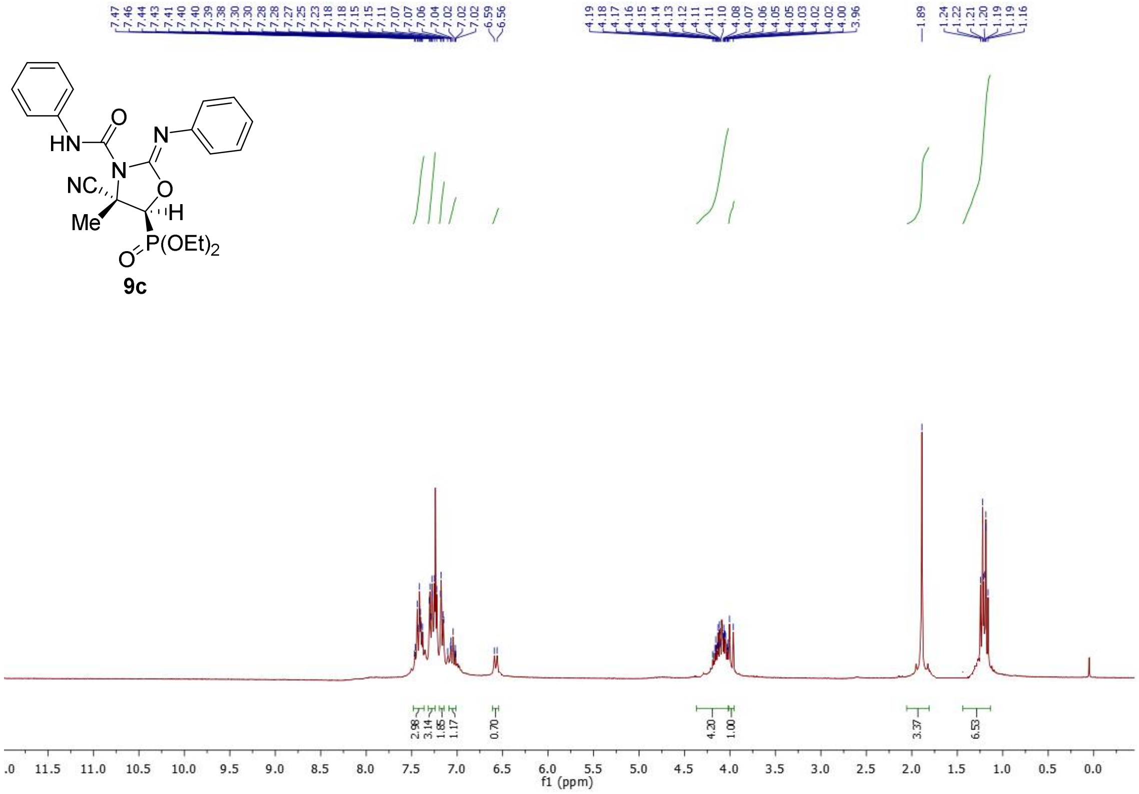

^{13}C {^{1}H} NMR (75 MHz, $CDCl_3$) of compound **9c**.

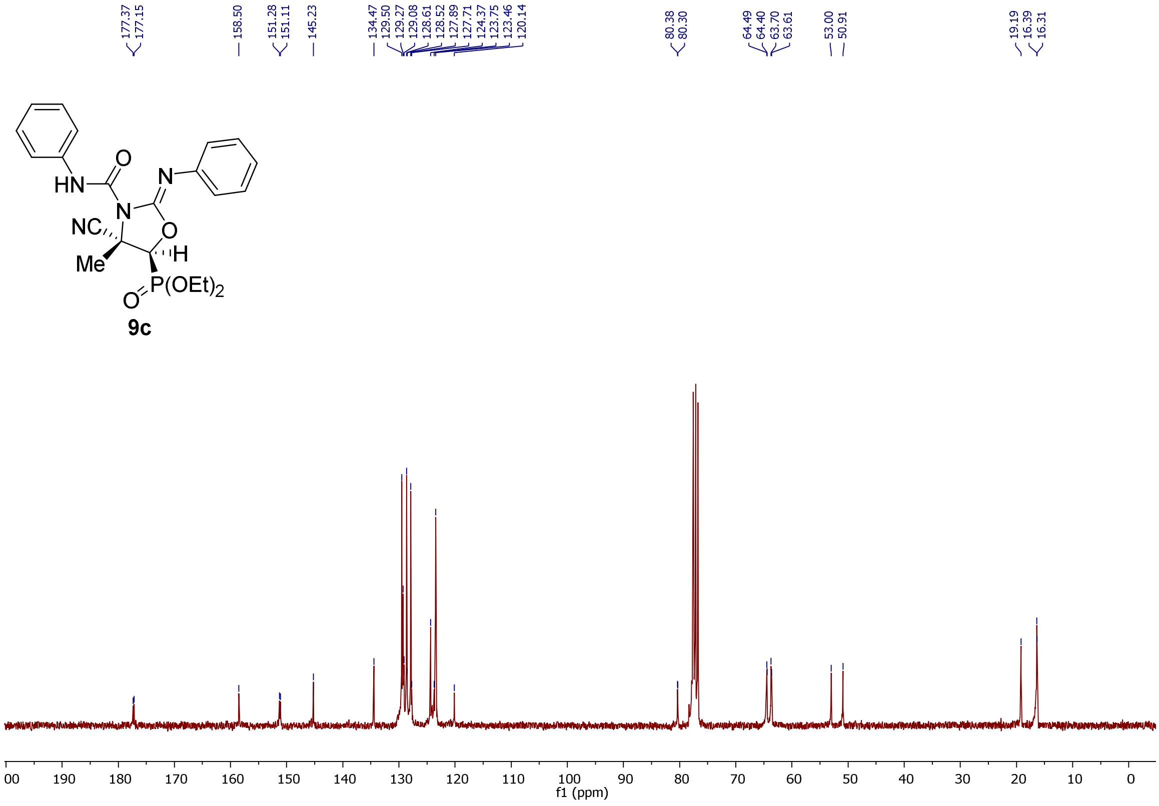

Supplement: Supplementary file 1 [file molecules-26-04265-s001.zip › molecules-1275866-supplementary.pdf]
